# Using a smartwatch and smartphone to assess early Parkinson’s disease in the WATCH-PD study over 12 months

**DOI:** 10.1038/s41531-024-00721-2

**Published:** 2024-06-12

**Authors:** Jamie L. Adams, Tairmae Kangarloo, Yishu Gong, Vahe Khachadourian, Brian Tracey, Dmitri Volfson, Robert D. Latzman, Joshua Cosman, Jeremy Edgerton, David Anderson, Allen Best, Melissa A. Kostrzebski, Peggy Auinger, Peter Wilmot, Yvonne Pohlson, Stella Jensen-Roberts, Martijn L. T. M. Müller, Diane Stephenson, E. Ray Dorsey, the Parkinson Study Group Watch-PD Study Investigators and Collaborators, Jamie L. Adams, Christopher Tarolli, Emma Waddell, Stella Jensen-Roberts, Julia Soto, Penelope Hogarth, Mastura Wahedi, Katrina Wakeman, Alberto J. Espay, Steven A. Gunzler, Camila Kilbane, Meredith Spindler, Matthew J. Barrett, Zoltan Mari, Liliana Dumitrescu, Kara J. Wyant, Kelvin L. Chou, Cynthia Poon, Tanya Simuni, Karen Williams, Nijee Luthra Caroline Tanner, Eda Yilmaz, Jeanne Feuerstein, David Shprecher, Andrew Feigin, Erica Botting

**Affiliations:** 1 Center for Health + Technology, University of Rochester Medical Center, Rochester, NY USA; 2 Department of Neurology, University of Rochester Medical Center, Rochester, NY USA; 3 Takeda Pharmaceuticals, Cambridge, MA USA; 4 AbbVie Pharmaceuticals, North Chicago, IL USA; 5 Biogen Inc., Cambridge, MA USA; 6 Clinical Ink, Horsham, PA USA; 7 Critical Path Institute, Tucson, AZ USA; 8 University of Rochester, Rochester, NY USA; 9 Oregon Health and Science University, Portland, OR USA; 10 University of Cincinnati, Cincinnati, OH, USA and James J. and Joan A Gardner Family Center for Parkinson’s Disease and Movement Disorders, Cincinnati, OH USA; 11 University Hospitals Cleveland Medical Center, Cleveland, OH USA; 12 University of Pennsylvania, Philadelphia, PA USA; 13 Baylor College of Medicine, Houston, TX USA; 14 Virginia Commonwealth University, Richmond, VA USA; 15 Cleveland Clinic Lou Ruvo Center for Brain Health, Las Vegas, NV USA; 16 University of Michigan, Ann Arbor, MI USA; 17 Northwestern University, Evanston, IL USA; 18 University of California, San Francisco, CA USA; 19 Sentara Neurology Specialists, Virginia Beach, VA USA; 20 University of Colorado Denver, Denver, CO USA; 21 Banner Sun Health Research Institute, Phoenix, AZ USA; 22 New York University, New York, NY USA; 23 University of South Florida, Tampa, FL USA

**Keywords:** Parkinson's disease, Parkinson's disease, Outcomes research

## Abstract

Digital measures may provide objective, sensitive, real-world measures of disease progression in Parkinson’s disease (PD). However, multicenter longitudinal assessments of such measures are few. We recently demonstrated that baseline assessments of gait, tremor, finger tapping, and speech from a commercially available smartwatch, smartphone, and research-grade wearable sensors differed significantly between 82 individuals with early, untreated PD and 50 age-matched controls. Here, we evaluated the longitudinal change in these assessments over 12 months in a multicenter observational study using a generalized additive model, which permitted flexible modeling of at-home data. All measurements were included until participants started medications for PD. Over one year, individuals with early PD experienced significant declines in several measures of gait, an increase in the proportion of day with tremor, modest changes in speech, and few changes in psychomotor function. As measured by the smartwatch, the average (SD) arm swing in-clinic decreased from 25.9 (15.3) degrees at baseline to 19.9 degrees (13.7) at month 12 (*P* = 0.004). The proportion of awake time an individual with early PD had tremor increased from 19.3% (18.0%) to 25.6% (21.4%; *P* < 0.001). Activity, as measured by the number of steps taken per day, decreased from 3052 (1306) steps per day to 2331 (2010; *P* = 0.16), but this analysis was restricted to 10 participants due to the exclusion of those that had started PD medications and lost the data. The change of these digital measures over 12 months was generally larger than the corresponding change in individual items on the Movement Disorder Society—Unified Parkinson’s Disease Rating Scale but not greater than the change in the overall scale. Successful implementation of digital measures in future clinical trials will require improvements in study conduct, especially data capture. Nonetheless, gait and tremor measures derived from a commercially available smartwatch and smartphone hold promise for assessing the efficacy of therapeutics in early PD.

## Introduction

Therapeutic progress for Parkinson’s disease (PD) has been slow, which has fueled interest in the development of more objective, precise, sensitive, and frequent measures of the disease that can be assessed in the real-world^1^. Digital tools, including smartwatches^2^, smartphones^3,4^, and wearable sensors^5,6^, offer the potential to provide such assessments. However, few studies^7^ have evaluated multiple such tools in a multicenter study aimed at individuals with early PD. Further, unlike many, our study evaluates broadly adopted and user-friendly devices.

In a multicenter observational study involving 82 individuals with early, untreated PD, we recently demonstrated that a commercially available smartwatch paired with a smartphone research application can assess key motor and non-motor features of the disease, including gait, psychomotor function, tremor, and voice^8^. These assessments differed significantly from those in an age-matched cohort of 50 individuals without PD and often correlated with accelerometers used for research and with traditional measures of the disease, such as the Movement Disorder Society—Unified Parkinson’s Disease Rating Scale (MDS-UPDRS)^8^. Here, we report on how these measures changed over 12 months. These results can inform the design of future clinical trials aimed at the growing population of individuals with PD^9^.

## Results

### Study participants

**Fig. 1:**
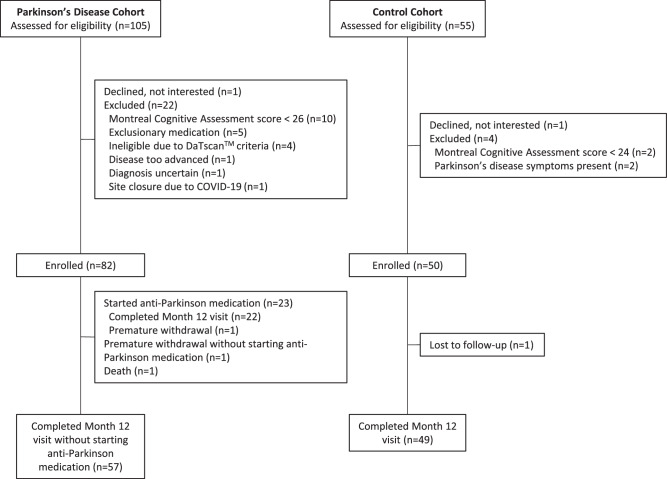
Flow of Participants. Flow of participants in the Watch-PD study over 12 months.

Eighty-two individuals with early, untreated PD and 50 age-matched controls consented to participate in the study at 17 research sites in the United States from June 2019 through December 2020. Of these individuals, 57 participants with PD completed the 12-month study without starting dopaminergic therapy (Fig. 1). Of the 23 that did begin dopaminergic therapy, 2 did so by three months, 11 by 6 months, 5 by nine months, and 5 by twelve months. Forty-nine of the 50 age-matched controls also completed month 12 assessments.

As shown in Table 1, the participants with PD were more likely to be men and were similar to those who enrolled in the Parkinson’s Progression Markers Initiative^10^. Average adherence for the active, at-home tasks did not differ between participants with PD (70.8%) and controls (70.9%; *P* = 0.98). There also was no significant difference in adherence based on gender (*P* = 0.25) or race (*P* = 0.48).

**Table 1:** Characteristics of research participants who enrolled and who completed the month-12 visit

		Enrolled baseline results			Enrolled and completed the month-12 visit month-12 results		
	Characteristic	PD cohort (*n* = 82)	Control cohort (*n* = 50)	*P* value	PD cohort without starting anti-Parkinson medication (*n* = 57)	Control cohort (*n* = 49)	*P* value
Demographic characteristics	Age, y	63.3 (9.4)	60.2 (9.9)	0.07	64.1 (9.4)	61.5 (9.7)	0.17
	Male, *n* (%)	46 (56)	18 (36)	0.03	32 (56)	18 (37)	0.05
	Race, *n* (%)			0.81			0.51
	White	78 (95)	48 (96)		55 (96)	47 (96)	
	Black or African American	0 (0.0)	0 (0)		0 (0.0)	0 (0)	
	Asian	3 (4)	1 (2)		2 (4)	1 (2)	
	Not specified	1 (1)	1 (2)		0 (0.0)	1 (2)	
	Hispanic or Latino, *n* (%)	3 (4)	1 (2)	0.99	2 (4)	1 (2)	0.99
	Education >12 years, *n* (%)	78 (95)	48 (96)	0.99	54 (95)	47 (96)	0.99
Clinical characteristics	Right or mixed handedness, *n* (%)	74 (90)	47 (94)	0.53	51 (89)	46 (94)	0.50
	Parkinson’s disease duration, months	10.0 (7.3)	N/A	N/A	22.1 (7.3)	N/A	N/A
	Hoehn and Yahr, *n* (%)			<0.001			<0.001
	Stage 0	0 (0)	49 (100)		0 (0)	47 (96)	
	Stage 1	19 (23)	0 (0)		7 (12)	1 (2)	
	Stage 2	62 (76)	0 (0)		49 (86)	1 (2)	
	Stage 3–5	1 (1)	0 (0)		1 (2)	0 (0)	
	MDS-UPDRS						
	Total score	35.2 (12.4)	5.9 (5.3)	<0.001	40.5 (14.2)	6.4 (5.0)	<0.001
	Part I	5.5 (3.6)	2.8 (2.6)	<0.001	5.9 (4.0)	3.0 (3.5)	<0.001
	Part II	5.6 (3.8)	0.4 (1.0)	<0.001	7.1 (4.7)	0.4 (1.1)	<0.001
	Part III	24.1 (10.2)	2.7 (3.5)	<0.001	27.4 (11.1)	2.9 (3.3)	<0.001
	Montreal Cognitive Assessment	27.6 (1.4)	28.1 (1.5)	0.04	27.5 (2.3)	28.5 (1.8)	0.02
	Parkinson’s Disease Quality of Life Questionnaire	7.7 (6.7)	N/A	N/A	9.1 (8.3)	N/A	N/A
	Geriatric Depression Scale (short version)	1.6 (1.9)	1.0 (1.2)	0.05	1.8 (1.5)	1.0 (1.4)	0.004
	REM Sleep Behavior Disorder Questionnaire	4.4 (3.1)	2.7 (2.0)	<0.001	4.5 (3.2)	2.5 (2.1)	<0.001
	Epworth Sleepiness Scale	4.9 (3.2)	4.6 (3.7)	0.66	4.8 (2.5)	4.4 (3.4)	0.50
	Scale for Outcomes in Parkinson’s Disease for Autonomic Symptoms	9.1 (5.1)	5.3 (4.2)	<0.001	9.2 (5.4)	5.1 (4.4)	<0.001

*PD* Parkinson’s disease, *N/A* not available, *MDS-UPDRS* Movement Disorder Society—Unified Parkinson’s Disease Rating Scale.

Results are mean (standard deviation) for continuous measures and *n* (%) for categorical measures.

One control cohort participant is missing baseline Hoehn and Yahr and MDS-UPDRS scores and one additional is missing the MDS-UPDRS part III and total scores.

### Gait

As shown in Table 2, the average measures of numerous gait assessments declined significantly among individuals with early PD. These measures included reductions in arm swing, gait speed, step length, and stride length. As measured by the smartwatch, the average (SD) arm swing in the clinic decreased from 25.9 (15.3) degrees at baseline to 19.9 degrees (13.7) at month 12 (*P* = 0.004). For mobile devices, differences were derived from the smartwatch (arm swing) and smartphone (the latter three) based on assessments done in the clinic but not at-home, and largely agree with measures from the research-grade sensors. In some cases, gait measures (e.g., cadence) were more variable when assessed at-home than in the clinic. The test–retest reliability of most gait measures was generally good with intra-class correlation coefficients (ICC) above 0.7 (Supplementary Table 1). Additional differences in the variability and asymmetry of the gait measures were observed as detailed in Supplementary Table 2.

**Table 2:** Change in selected endpoints measured in-clinic in PD over 12 months

*MDS-UPDRS* the Movement Disorder Society-Sponsored Revision of the Unified Parkinson’s Disease Rating Scale, *SDMT* Symbol-Digit Modalities Test, *VSWM* Visuospatial Working Memory Task, *PD* Parkinson’s Disease.

For the individual digital measures, we calculated a standardized change by dividing the mean of the difference between follow-up (month 12) and baseline assessments by the standard deviation of their difference. The magnitude of the standardized change in arm swing, gait speed, step length, and stride length ranged from 0.57 to 0.66 compared to 0.24 for item 2.12 on the MDS-UPDRS (self-reported problems with walking and balance) and 0.06 for item 3.10 on the MDS-UPDRS (rater evaluation of gait).

### Tremor

**Fig. 2:**
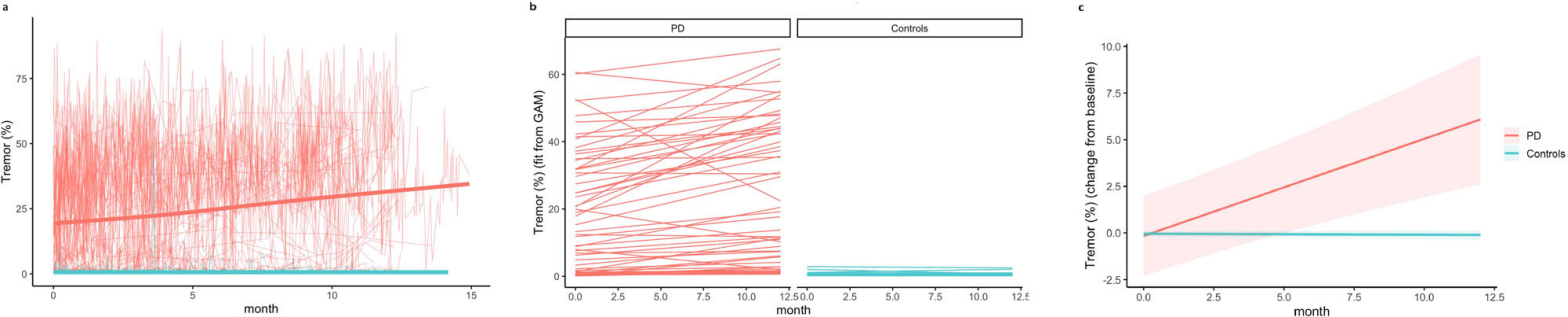
Change over 12 months in proportion of waking hours with tremor as measured by the smartwatch for individuals with Parkinson’s disease versus controls. This figure illustrates the modeling approach. **a** shows the change in tremor for each participant. **b** shows subject-specific line fits across the year, while (**c**) shows predicted change from baseline for each cohort.

As shown in Fig. 2, the average (SD) proportion of time individuals with PD experienced rest tremor at-home increased from 19.3% (18.0%) at baseline to 25.6% (21.4%; *P* < 0.001) at month 12 as assessed by the smartwatch. Among the 57 participants with PD for whom there was sufficient passive data to evaluate, thirteen had tremor for less than 3% of the day, which changed little over one year. Control participants had minimal tremor (less than 1% of the day) that did not change over the study’s duration. The standardized change in the proportion of the day with tremor as assessed by the smartwatch was 0.65 compared to 0.40 for item 2.10 on the MDS-UPDRS (self-reported tremor) or 0.53 for item 3.18 on the MDS-UPDRS (rater evaluation of constancy of tremor).

### Psychomotor

**Fig. 3:**
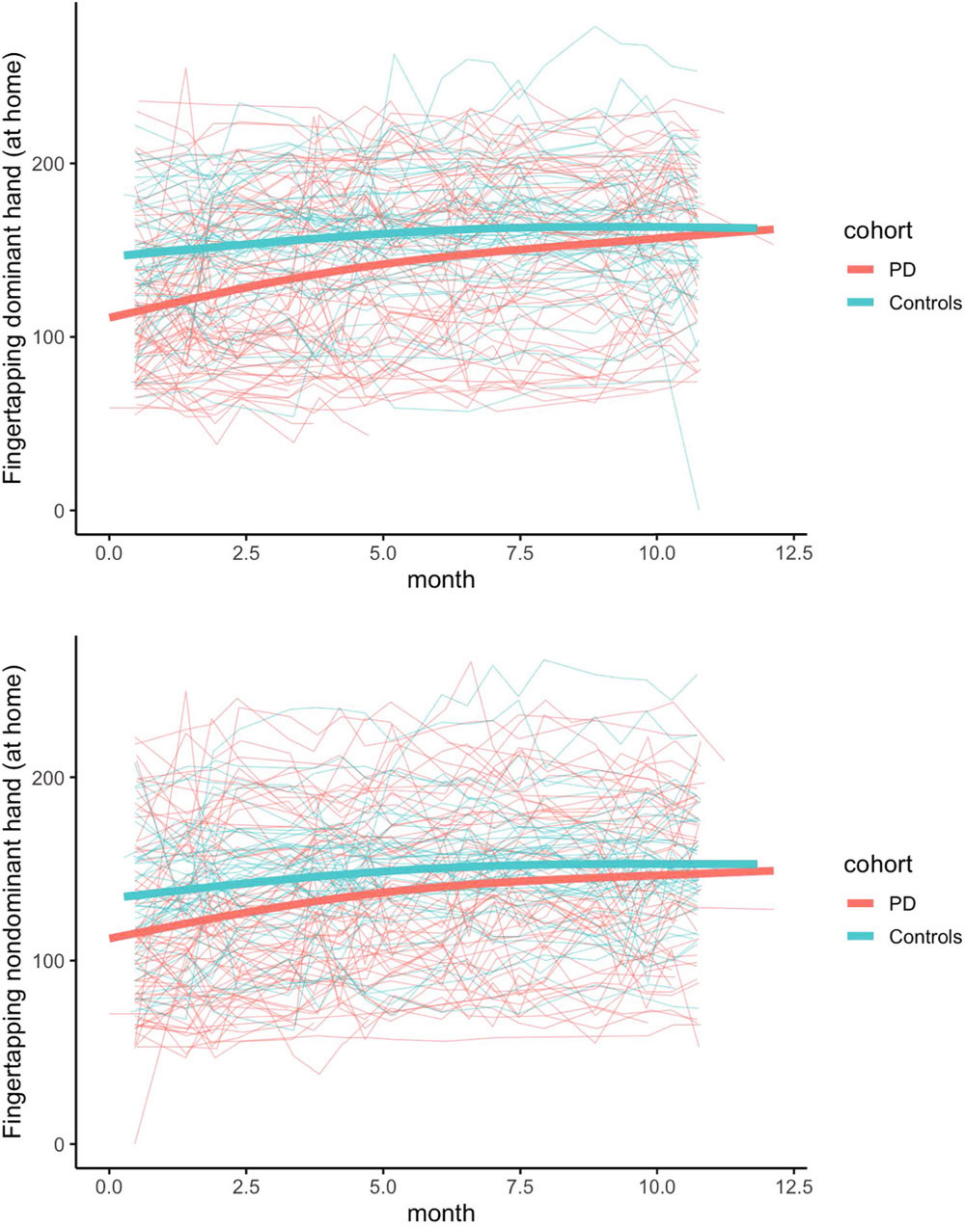
Dominant hand (top) finger tapping at-home and non-dominant hand (bottom) finger tapping at-home.

Notably, several psychomotor metrics showed significant changes over 12 months both for individuals with PD (Table 2) and for controls (Table 3). For example, the average total taps in-clinic and at-home increased significantly in both the dominant and non-dominant hands in both groups (Fig. 3). Furthermore, the average inter-tap interval (time between taps) in both hands decreased significantly in both groups with the exception of the individuals with PD at-home in the dominant hand. The average inter-tap interval for individuals with PD also decreased significantly over 12 months on both the more and less-affected sides. On the more-affected side, the average inter-tap interval decreased at-home from 206.1 ms (65.1 ms) at month 0 to 177.6 ms (92.5 ms) at month 12. On the less-affected side, the average inter-tap interval decreased at-home from 176.9 ms (69.3 ms) at month 0 to 138.9 ms (90.8 ms) at month 12. For the fine motor task, the average number completed significantly increased over 12 months in PD and controls for both hands in-clinic and at-home. The number completed in the fine motor task also increased within PD participants in both the more and less-affected sides. However, in both the finger tapping and fine motor tasks, the magnitude of change was greater for controls than in individuals with PD, suggesting a reduced longitudinal performance in the PD group. Test–retest reliability for the psychomotor tasks ranged from poor (ICC of 0.28 for the inter-tap interval on the non-dominant hand in the clinic) to excellent (ICC of 0.95 for finger tapping total taps at-home on the dominant hand).

**Table 3:** Change in selected endpoints in control participants over 12 months

*CI* confidence interval, *MDS-UPDRS* the Movement Disorder Society-Sponsored Revision of the Unified Parkinson’s Disease Rating Scale, *SDMT* Symbol-Digit Modalities Test, *VSWM* Visuospatial Working Memory Task.

### Cognition

As shown in Supplementary Table 2, the average time to completion on Trails A in people with PD decreased over 12 months in the clinic from 27,843 ms (5745 ms) at baseline to 23,520 ms (6684 ms) at month 12 and at-home from 24,719 ms (5109 ms) at month 0 to 23,091 ms (6599 ms) at month 12. Furthermore, the average time to completion on Trails B also decreased both in the clinic from 24,870 ms (3838 ms) at baseline to 19,656 ms (3913 ms) at month 12 and at-home from 23,245 ms (3789 ms) at month 0 to 19,476 ms (3791 ms) in people with PD. Similar trends were seen for controls in performance on both Trails A and B. The discrepancy between time to complete Trails B and Trails A (Trails B–Trails A) increased, but not significantly, in both groups. The average number correct on the Symbol-Digit Modalities Test in-clinic and at-home increased significantly over the 12 months in both groups. The average percent correct on the visuospatial working memory test also increased significantly over 12 months in both groups in-clinic and at-home (Tables 2 and 3). The test–retest reliability measures for cognition were weak to moderate across tasks, with the lowest ICC at 0.03 for Trails B-A in the clinic and the highest ICC at 0.55 for total time to complete Trails A.

### Speech

The at-home digital speech composite scores also increased significantly from 1.2 (1.9) at baseline to 1.7 (2.0; *P* = 0.03) at month 12 for individuals with PD (Table 2). No significant change was seen in the in-clinic speech composite or in any speech measures for the control cohort. As seen in Supplementary Table 1, test–retest reliability for speech measures was generally lower than for gait measures, with ICCs in the range of 0.6–0.7 for the most reliable measures. As shown in Supplementary Table 2, the composite metric shows improved reliability over several individual speech features. The composite score shows a standardized change in magnitude of 0.25 compared to 0.33 for item 2.01 (self-reported speech) or 0.66 for item 3.01 (rater evaluation of speech).

### Activity

At baseline, individuals with PD (*n* = 45) had a trend toward a lower average (SD) daily step count (3494 (1930) steps daily) compared to control participants (*n* = 15) (4930 (3270) steps daily; *P* = 0.13) as assessed by the smartwatch. After applying the inclusion criteria detailed in the missing data section, we observed that participants with PD wore the smartwatch for 14.4 hours per day on average compared to 13.5 hours per day for controls (*P* = 0.03). To reduce the impact of differences in wear time, we also looked at steps per hour. Individuals with PD (*n* = 45) walked 238 (129) steps per hour at baseline, while control participants (*n* = 15) walked 362 (214) steps per hour (*P* < 0.001). Considering the pandemic’s impact on activity level, interpreting the longitudinal change in the step counts requires caution. At month 12, only 10 individuals with PD who were still off medication had passive data for analysis. Due to the limitation of sample size, we did not apply the model to the step count data. For the 10 individuals with PD who were not taking dopaminergic medication, the mean number of steps taken daily decreased from 3052 (1306) to 2331 (2010) steps (*P* = 0.16), and the number of steps per hour decreased from 198 (82) steps per hour at baseline to 159 (142) steps per hour at month 12 (*P* = 0.29) (Supplementary Table 3).

### Clinical measures

Overall, composite measures of the MDS-UPDRS had the largest standardized change in this observational study (Table 2). For individuals with PD, the standardized change from baseline to month 12 for the MDS-UPDRS part I was 0.30, for part II was 1.16, and for part III was 1.17. The standardized change for the sum of parts I, II, and III for those with Parkinson’s was 1.33.

## Discussion

Over 12 months, digital measures derived from a commercially available smartphone and smartwatch changed significantly in multiple domains, most notably gait and tremor, among individuals with early PD. Combined with the observed differences among individuals with and without PD on numerous measures at baseline, these digital assessments hold promise (Table 4) for helping evaluate the efficacy of future therapies^1,3,7,11^ and monitoring individuals in this population. Use of digital tools to quantify manifestations of Parkinson’s disease has attracted increasing interest over the past 2 decades, and the present results build on many prior studies summarized in review articles^12–15^ by providing 12-month longitudinal follow-up, comparing an early, unmedicated PD population to an age-matched control group, and including digital assessments that span a range of functional domains. Consistent with prior longitudinal reports^6,16,17^ and predictions from cross-sectional studies^18–20^, well-established gait parameters including stride length, gait speed, and arm swing amplitude were among the mobility measures that showed significant longitudinal change specifically in the cohort of people with PD. However, the finding that these parameters did not show significant longitudinal progression when measured from the unsupervised walking test done at-home was unexpected. One possible explanation for this finding is that the in-clinic timed walk protocol required a 10-meter straight path, while the unsupervised walking path would be determined by the participant and may have involved much shorter segments, circular or curving trajectories, and so forth. This highlights one of the challenges with remote digital monitoring: diminished control over the details of task performance. With that in mind, it was encouraging to see that some of the measures showing the largest change over 12 months were task-independent and passively assessed (tremor, steps).

**Table 4:** Summary of findings to date from the Watch-PD study

Domain	Findings	Device	Assessment	Comment
Gait • Arm swing • Gait speed	• Reduced at baseline compared to controls • Decreases significantly over 12 months	Smartwatch and smartphone	Active	Promising measure for assessment in future clinical trials in PD population
Tremor • Proportion of day with tremor	• Increased at baseline compared to controls • Increased significantly over 12 months	Smartwatch	Passive	Promising measure for assessing the efficacy of therapies aimed at reducing tremor
Neuropsychology • Finger tapping	• Reduced at baseline compared to controls but no significant progression over 12 months	Smartphone	Active	Results may have been complicated by learning effects
Cognitive • Trails tests	• Reduced at baseline compared to controls but no significant progression	Smartphone	Active	Other measures of cognition may be more sensitive to change
Speech • Composite score	• Reduced at baseline compared to controls and shows modest progression	Smartphone	Active	Beneficial for differentiating PD from controls
Activity • Steps taken daily	• Reduced at baseline compared to controls and may decrease with time	Smartwatch	Passive	Promising measure but larger datasets required

This study engaged multiple stakeholders, including the pharmaceutical industry, regulators, investigators, and individuals with PD^21^. Consistent with recent initiatives^22^ from the U.S. Food and Drug Administration (FDA), this study included the “voice of the patient”^23^. To that end, we conducted qualitative interviews with participants from this study who rated gait/balance, tremor, and fine motor measures as the most meaningful to them^24,25^.

Gait speed is considered the “functional vital sign”^26^ and is associated with mortality in older adults^27^. It also has been accepted by European regulators as a digital endpoint in Duchenne muscular dystrophy^28^ and has already been the basis for an approved therapy for multiple sclerosis^29^. In PD, gait speed has been shown to be affected early in disease, including during the prodromal period^30^ and to decrease over one year when measured at-home in individuals at different stages of the disease^16^, a finding reinforced in this study.

Activity is a widely used measure of health in everyday life by millions. Activity has also been accepted as a digital endpoint (moderate-to-vigorous physical activity) by the FDA for idiopathic pulmonary fibrosis^31^, and is affected early in the course of PD, including during the prodromal period^32,33^. Tremor, while not a universal symptom in PD, is an important feature to study participants^24^.

The standardized changes observed for several individual digital measures (e.g., arm swing) were modest and not as large as the MDS-UPDRS Part 3 summation itself. In our study, digital measures of gait and tremor appear to be among the most promising as longitudinal progression markers, with standardized changes from digital measures often exceeding the observed changes in the corresponding individual items of the MDS-UPDRS. Importantly, the increase in standardized changes in these measures would translate directly into smaller sample sizes (for example, under the assumption of 40% treatment effect on tremor which has a standardized change of ~0.7, the required sample in each arm is 202). Supplementary Table 4 provides estimated sample size by standardized change and assumed treatment effect for studies powered based on these measures. These digital measures also showed good test–retest reliability (ICCs generally greater than 0.7). In-clinic comparisons with research-grade accelerometers also displayed consistency between the measurement devices here and in our baseline paper^8^.

In contrast to assessments of gait and tremor, performance on measures of psychomotor and cognitive function appeared to improve over 12 months in both PD and control cohorts. Performance among controls generally reached a stable asymptote faster than individuals with PD suggesting that the latter group may need greater exposure to a given task to reach stable performance. In the future, these measures could be used to evaluate changes in individuals, in addition to at the group leveI.

Unlike gait and tremor, the standardized changes in the speech digital measures were smaller than the corresponding MDS-UPDRS individual items. The sensitivity of the digital speech measures can likely be improved by using alternative analytical approaches. For example, Rusz and colleagues^34^ found modest but significant progression in speech in early PD and identified voice onset time (not assessed in this study) as a key measure. In our study, digital speech assessments were also hampered by several data quality issues as previously discussed^8^. Finally, the lower test–retest reliability of speech features (as compared to gait) in our dataset may help explain why progression was significant at-home but not in-clinic. At-home measurements were much more frequent than those in the clinic (and also had less background noise^8^) and thus may have been better able to smooth out measurement noise and generate a higher signal-to-noise ratio. Our overall findings are generally consistent with ref. ^34^; we saw modest overall progression. Both studies also found that among speech measures, decreased pitch range was the strongest differentiator of PD and control cohorts at baseline but did not show progression in the PD group over 1 year.

In addition to individual digital measures, future efforts may seek to develop composite digital measures^4^ that combine assessments of motor, non-motor, and social function, both in the clinic and in the real world. Ideally, such measures would accurately track subject-level progression. Such a measure could complement traditional rating scales by reflecting assessments (e.g., proportion of day with tremor, overall activity) that are likely meaningful, but cannot be tested with a scale administered episodically. These composite measures could also be more sensitive to change than individual measures, just as the summary measures of the MDS-UPDRS can detect change better than individual items.

This study has several limitations. Among them are the COVID-19 pandemic, missing data, adherence, lack of standardization, learning effects, and a limited scope of assessments. The pandemic occurred in the midst of this study leading us to transition some clinic visits to remote ones and potentially reduced physical activity among all participants^35^. The pandemic, though, did highlight the need for real-world measures of disease. Missing data due to software issues limited our ability to collect data for some assessments (e.g., number of steps taken) in many individuals. Generalized Additive Models (GAM) allowed for the fitting of individualized curves for each participant and can handle the uneven time sampling seen with at-home data, but the model can struggle to capture the underlying group patterns when limited data are available. Thus we were not able to apply these models for step count. The modeled results (reported in Supplementary Table 2) differed in their absolute values from empirical ones (reported in Supplementary Table 3), but the overall results were generally consistent. Real-time monitoring of data and adherence, managing software changes, and maintaining close contact with participants could all reduce missing data.

Standardizing the use of digital devices was also difficult. In this study, participants with PD were supposed to wear the smartwatch on the more-affected side; however, some wore the watch on different wrists and were not always consistent. For some measures (e.g., cadence), home assessments were more variable than clinical ones perhaps due to greater variability in the setting and less support from trained staff. Some tasks (e.g., finger tapping) also appeared to be affected by practice effects, as the speed with which they were conducted improved in both groups. Including repeated tests of these tasks at multiple visits (e.g., screening and baseline) might help mitigate these effects. Due to study design, time between visits used for test–retest reliability results was 4–6 weeks, a duration which could reduce precision of this measurement. Future studies may opt to repeat digital assessments during the same visit or at a shorter interval to better evaluate test–retest reliability. Finally, the measures in this study were more focused on motor features while smartphones and smartwatches can also assess valuable non-motor^36^ (e.g., autonomic function^37^, sleep^38^) and social metrics (e.g., time or distance away from home^39^) of PD.

Limitations notwithstanding, in a study designed to replicate the conduct of a multicenter clinical trial in individuals with early, untreated PD, we found numerous valuable digital measures derived from a commercial smartwatch and smartphone. The response of these metrics to medications^4,40^ remains to be established. However, this study bring us closer to having meaningful digital measures for future use in PD clinical trials.

## Methods

### Study design, setting, participants

As described previously, WATCH-PD (Wearable Assessment in The Clinic and at Home in PD, NCT03681015) is a 12-month, multicenter observational study that evaluated the ability of digital devices to assess disease features and progression in persons with early, untreated PD^8^. Participants, recruited from 17 Parkinson Study Group research sites, were evaluated in the clinic and at-home. In-person visits occurred at screening/baseline and then at months 1, 3, 6, 9, and 12. Due to the COVID-19 pandemic, most month-3 visits were converted to remote visits via video or phone, and participants could elect to complete additional visits remotely.

We sought to evaluate a population similar to the Parkinson’s Progression Markers Initiative (PPMI). For those with PD, the principal inclusion criteria were age 30 or greater at diagnosis, disease duration less than two years, and Hoehn & Yahr stage two or less. Exclusion criteria included baseline use of dopaminergic or other PD medications and an alternative Parkinsonian diagnosis. Control participants without PD or other significant neurologic diseases were age-matched to the PD cohort.

### Ethics

The WCG^TM^ Institutional Review Board approved the procedures used in the study, and there was full compliance with human experimentation guidelines. All participants provided written informed consent before study participation.

### Data sources/measurement

As described previously^8^, this study used three devices: research-grade wearable “Opal” sensors (APDM Wearable Technologies, a Clario Company), an Apple Watch 4 or 5, and an iPhone 10 or 11 (Apple, Inc.) running a smartphone application specifically for PD (BrainBaseline™). The smartphone application consisted of cognitive, speech, and psychomotor tasks including Trail Making Test, modified Symbol-Digit Modalities Test, Visuospatial Working Memory Task, phonation, reading, diadochokinetic speech tasks, two-timed fine motor tests, and tremor, gait, and balance tasks.

During in-clinic visits, six research-grade wearable sensors with an accelerometer, gyroscope, and magnetometer were placed on the sternum, lower back, and on each wrist and foot. Smartphone application tasks were conducted at each clinic visit and at-home every 2 weeks on the smartphone. The smartphone was worn in a lumbar sport pouch during gait and balance tests. After each in-person visit, participants wore the smartwatch on their more-affected side and tracked symptoms on the smartphone daily for at least 1 week.

Movement data was collected from the wearable sensors using Mobility Lab software (APDM Wearable Technologies, a Clario Company), and measures were extracted using custom algorithms written in Python (Wilmington, DE). Gait features were extracted from the smartwatch and smartphone using na modified version of GaitPy^41^. The algorithm for extracting arm swing is a refactored version implemented in python based on ref. ^13^. Phonation and reading files were processed using custom Python code with features computed using the Parselmouth interface to Praat and the Librosa library. Common speech endpoints, such as jitter, shimmer, pitch statistics, and Mel Frequency Cepstral Coefficients (MFCC), were computed.

Accelerometry data and tremor scores were collected from the smartwatch via Apple’s Movement Disorders Application Programming Interface during the passive monitoring periods^42^. The Movement Disorders API generates tremor classification scores (none, slight, mild, moderate, strong, or unknown) for each 1-min period, and the fraction of time spent in each category was calculated for each participant^2^.

### Clinical measures

Participants completed traditional rating scales including the MDS-UPDRS Parts I-III, Montreal Cognitive Assessment, Modified Hoehn and Yahr, Geriatric Depression Scale, REM Sleep Behavior Disorder Questionnaire, Epworth Sleepiness Scale, Scale for Outcomes in Parkinson’s Disease for Autonomic Symptoms, and the Parkinson’s Disease Questionnaire-8.

### Study size

The study was powered to detect a mean change over 12 months for a digital endpoint with superior responsiveness to MDS-UPDRS Part III. The mean change in part III from baseline to year one in individuals with early, untreated PD in the PPMI study was 6.9 with a standard deviation of 7.0. Allowing for up to half of participants to begin dopaminergic therapy over 12 months and 15% drop out, the study aimed to recruit at least 75 participants with PD to yield 30 participants completing the study off medication. The study had more than 95% power to detect a true change of 6.9 units using a one-sample t-test and a two-tailed 5% significance.

### Feature extraction

Features (e.g., of gait, speech) were extracted following the same methods as described in the baseline manuscript^8^ with the exception of step count and a composite measure of speech, which were not previously analyzed. Based on a previous study by Rusz and colleagues^34^, a composite speech score was formed by normalizing selected individual features using the normative cohort statistics at baseline (i.e., subtracting off the control baseline mean, dividing by the control baseline standard deviation). The composite was formed using the four speech features (log-transformed mean pause time in the reading task, monopitch features in the reading task, MFCC2 from the phonation task and cepstral peak prominence from the phonation task) that differed between individuals with and without PD at baseline^8^. Step count statistics were acquired using the Forest Oak package implemented in Python^43,44^. To avoid sleep time, only data from the watch between 06:00 and 23:59 local time were processed by the Forest Oak package.

### Statistical methods

At study start, all participants with PD were drug-naïve, and according to pre-specified analysis plans, all measurements were included up to the point where participants started medications. Measurements from times after the start of PD medications were ignored. Statistical analysis was implemented in R version 4.2.2. *P* values < 0.05 were considered statistically significant, and no adjustment for multiple comparisons was made.

For longitudinal modeling, we applied mixed effects modeling to capture individual-specific differences and allowed for flexible evolution of patient progression versus time (avoiding strong constraints, for example, the assumption that progression has a linear slope over time). We conducted our statistical analysis via GAM, executed using the gam function from the mgcv package in the R programming environment^45^. The GAM framework provided a robust way for measuring complex and non-linear interdependencies among variables. Separate models were fitted for the cohort of individuals with PD and controls in order to facilitate understanding of progression in the Parkinson’s-only cohort for planning future clinical trials.

We used the relative measure of change from baseline as our response variable. Once the relative measure was computed, the data point at baseline was removed so that the model was not forced to pass through origin. For predictors, our model included variable value at baseline and time as fixed effects. The time variable is modeled as a smooth function, namely a pchip spline fit with four basis functions to allow estimation of distinct smooth functions^46^. For random effects, we introduced a random intercept and random smooth time trajectories for each participant. These terms accounted for participant-specific variations.

For passive smartwatch data (tremor and step count measures), we weighted measurements based on watch wear time so days with longer wear time received more weight. For model fitting, we employed the Restricted Maximum Likelihood estimation technique. Model selection was performed using Akaike information criterion (AIC) to allow for comparison between models, for example allowing us to compare models with and without participant-level trajectory fitting using AIC, ultimately leading to our present model.

Test–retest reliability was assessed by comparing the first and second clinic visits (separated by 6 weeks) under the assumption that Parkinsonian symptoms would have progressed little over this time frame. For at-home data, we reported three adjacent pairwise comparisons from the first four at-home measures (1st vs. 2nd, 2nd vs. 3rd, 3rd vs. 4th).

### Missing data

In the main analysis, data from participants were considered until they began taking PD medications. Data from premature withdrawals were not included. For clinic measurements, each patient must have contributed at least two data points, while for at-home measurements, each participant had to have at least three data points for inclusion in the analysis. If a participant had missing data for an outcome or as part of a necessary algorithm, that data point was excluded for that analysis. Values of zero (i.e., did not attempt the task) were also excluded. Detailed reasons for missing data are outlined in Supplementary Table 3 of the baseline paper^8^.

For step count data inclusion, we considered a participant’s data if there were a minimum of 9 h of wear time during the waking period (06:00–23:59). This 9-h threshold is based on openly available step count data from the PPMI dataset, where step counts are collected using Verily Study Watches^47^. Analysis of the PPMI dataset reveals a Pearson’s correlation exceeding 0.9 between step counts derived from at least nine randomly selected hours within the wake period (6:00 am–23:59 pm) and the overall step count for the entire waking period.

### Reporting summary

Further information on research design is available in the Nature Research Reporting Summary linked to this article.

### Supplementary information

Supplementary Material

Reporting Summary

## Data Availability

Data are available to members of the Critical Path for Parkinson’s Consortium 3DT Initiative Stage 2. For those who are not a part of 3DT Stage 2, a proposal may be made to the WATCH-PD Steering Committee (via the corresponding author) for de-identified datasets.

## Code availability

Custom Python code used for feature extraction and R code for statistical analysis is available from the authors upon request.
