# Supplementary material for: Using a smartwatch and smartphone to assess early Parkinson’s disease in the WATCH-PD study over 12 months
Source: NPJ Parkinsons Dis. 2024 Jun 12;10:112. doi: 10.1038/s41531-024-00721-2 (PMC11169239; doi:10.1038/s41531-024-00721-2)
Supplement: Supplementary file 1 — Supplementary Material [file 41531_2024_721_MOESM1_ESM.pdf]

Supplementary Table 1: Test-Retest Reliability Comparison of Data Collected via from smartphone/smartwatch/research-grade accelerometer at Baseline and 1st clinic visit, and pairwsise at home (1st vs 2nd, 2nd vs 3rd, 3rd vs 4th when available). ICC, Intraclass Correlation Coefficient; CI, Confidence Interval. MDS-UPDRS, the Movement Disorder Society-Sponsored Revision of the Unified Parkinson's Disease Rating Scale. SDMT, Symbol-Digit Modalities Test. VSWM, Visuospatial Working Memory Task. L, Left. R, Right. GCT, Gait Cycle Time.

**Feature**	**Domain**	**Task**	**Device**	**Location**	**Pair**	**n**	**ICC**	**95% CI**	**Spearman r**
Finemotor dominant hand	Cognitive/Pschomotor	Finemotor	Smartphone	clinic	Baseline vs. 1st clinic visit	115	0.63	(0.5 0.73)	0.53
Finemotor dominant hand	Cognitive/Pschomotor	Finemotor	Smartphone	home	1st vs 2nd at-home measurement	123	0.62	(0.49 0.72)	0.6
Finemotor dominant hand	Cognitive/Pschomotor	Finemotor	Smartphone	home	2nd vs 3rd at-home measurement	120	0.6	(0.48 0.71)	0.59
Finemotor dominant hand	Cognitive/Pschomotor	Finemotor	Smartphone	home	3rd vs 4th at-home measurement	118	0.63	(0.5 0.72)	0.62
Finemotor nondominant hand	Cognitive/Pschomotor	Finemotor	Smartphone	clinic	Baseline vs. 1st clinic visit	115	0.63	(0.51 0.73)	0.58
Finemotor nondominant hand	Cognitive/Pschomotor	Finemotor	Smartphone	home	1st vs 2nd at-home measurement	123	0.64	(0.52 0.73)	0.63
Finemotor nondominant hand	Cognitive/Pschomotor	Finemotor	Smartphone	home	2nd vs 3rd at-home measurement	120	0.62	(0.49 0.72)	0.6
Finemotor nondominant hand	Cognitive/Pschomotor	Finemotor	Smartphone	home	3rd vs 4th at-home measurement	118	0.7	(0.59 0.78)	0.67
Fingertapping dominant hand	Cognitive/Pschomotor	Fingertapping	Smartphone	clinic	Baseline vs. 1st clinic visit	120	0.67	(0.56 0.76)	0.75
Fingertapping dominant hand	Cognitive/Pschomotor	Fingertapping	Smartphone	home	1st vs 2nd at-home measurement	123	0.8	(0.73 0.86)	0.81
Fingertapping dominant hand	Cognitive/Pschomotor	Fingertapping	Smartphone	home	2nd vs 3rd at-home measurement	121	0.85	(0.79 0.89)	0.85
Fingertapping dominant hand	Cognitive/Pschomotor	Fingertapping	Smartphone	home	3rd vs 4th at-home measurement	120	0.95	(0.92 0.96)	0.95
Fingertapping nondominant hand	Cognitive/Pschomotor	Fingertapping	Smartphone	clinic	Baseline vs. 1st clinic visit	120	0.69	(0.58 0.77)	0.83
Fingertapping nondominant hand	Cognitive/Pschomotor	Fingertapping	Smartphone	home	1st vs 2nd at-home measurement	123	0.76	(0.67 0.82)	0.81
Fingertapping nondominant hand	Cognitive/Pschomotor	Fingertapping	Smartphone	home	2nd vs 3rd at-home measurement	121	0.86	(0.81 0.9)	0.89
Fingertapping nondominant hand	Cognitive/Pschomotor	Fingertapping	Smartphone	home	3rd vs 4th at-home measurement	120	0.92	(0.89 0.94)	0.92
Intertap intervals (ms) dominant hand	Cognitive/Pschomotor	Intertap intervals	Smartphone	clinic	Baseline vs. 1st clinic visit	115	0.24	(0.06 0.41)	0.49
Intertap intervals (ms) dominant hand	Cognitive/Pschomotor	Intertap intervals	Smartphone	home	1st vs 2nd at-home measurement	123	0.47	(0.32 0.6)	0.5
Intertap intervals (ms) dominant hand	Cognitive/Pschomotor	Intertap intervals	Smartphone	home	2nd vs 3rd at-home measurement	121	0.65	(0.54 0.74)	0.64
Intertap intervals (ms) dominant hand	Cognitive/Pschomotor	Intertap intervals	Smartphone	home	3rd vs 4th at-home measurement	119	0.54	(0.4 0.66)	0.49
Intertap intervals (ms) nondominant hand	Cognitive/Pschomotor	Intertap intervals	Smartphone	clinic	Baseline vs. 1st clinic visit	116	0.28	(0.1 0.44)	0.43
Intertap intervals (ms) nondominant hand	Cognitive/Pschomotor	Intertap intervals	Smartphone	home	1st vs 2nd at-home measurement	123	0.51	(0.36 0.63)	0.53
Intertap intervals (ms) nondominant hand	Cognitive/Pschomotor	Intertap intervals	Smartphone	home	2nd vs 3rd at-home measurement	121	0.32	(0.15 0.47)	0.28
Intertap intervals (ms) nondominant hand	Cognitive/Pschomotor	Intertap intervals	Smartphone	home	3rd vs 4th at-home measurement	120	0.58	(0.45 0.69)	0.47
Percentage total correct from SDMT	Cognitive/Pschomotor	SDMT	Smartphone	clinic	Baseline vs. 1st clinic visit	119	0.44	(0.28 0.57)	0.49
Percentage total correct from SDMT	Cognitive/Pschomotor	SDMT	Smartphone	home	1st vs 2nd at-home measurement	126	0.57	(0.44 0.67)	0.57
Percentage total correct from SDMT	Cognitive/Pschomotor	SDMT	Smartphone	home	2nd vs 3rd at-home measurement	123	0.53	(0.39 0.65)	0.52
Percentage total correct from SDMT	Cognitive/Pschomotor	SDMT	Smartphone	home	3rd vs 4th at-home measurement	123	0.66	(0.54 0.75)	0.63
Percentage total correct from VSWM	Cognitive/Pschomotor	VSWM	Smartphone	clinic	Baseline vs. 1st clinic visit	118	0.37	(0.2 0.51)	0.36
Percentage total correct from VSWM	Cognitive/Pschomotor	VSWM	Smartphone	home	1st vs 2nd at-home measurement	123	0.34	(0.17 0.49)	0.27
Percentage total correct from VSWM	Cognitive/Pschomotor	VSWM	Smartphone	home	2nd vs 3rd at-home measurement	121	0.35	(0.19 0.5)	0.29
Percentage total correct from VSWM	Cognitive/Pschomotor	VSWM	Smartphone	home	3rd vs 4th at-home measurement	120	0.35	(0.18 0.5)	0.35
Trail making task A time (ms)	Cognitive/Pschomotor	Trails	Smartphone	clinic	Baseline vs. 1st clinic visit	109	0.43	(0.27 0.57)	0.44
Trail making task A time (ms)	Cognitive/Pschomotor	Trails	Smartphone	home	1st vs 2nd at-home measurement	126	0.49	(0.35 0.61)	0.52
Trail making task A time (ms)	Cognitive/Pschomotor	Trails	Smartphone	home	2nd vs 3rd at-home measurement	124	0.48	(0.33 0.6)	0.41
Trail making task A time (ms)	Cognitive/Pschomotor	Trails	Smartphone	home	3rd vs 4th at-home measurement	123	0.55	(0.41 0.66)	0.59
Trail making task B minus trail making task A (ms)	Cognitive/Pschomotor	Trails	Smartphone	clinic	Baseline vs. 1st clinic visit	92	-0.03	(-0.23 0.17)	-0.04
Trail making task B minus trail making task A (ms)	Cognitive/Pschomotor	Trails	Smartphone	home	1st vs 2nd at-home measurement	126	0.14	(-0.03 0.31)	0.15
Trail making task B minus trail making task A (ms)	Cognitive/Pschomotor	Trails	Smartphone	home	2nd vs 3rd at-home measurement	123	0.06	(-0.12 0.23)	0.01
Trail making task B minus trail making task A (ms)	Cognitive/Pschomotor	Trails	Smartphone	home	3rd vs 4th at-home measurement	121	0.11	(-0.07 0.28)	0.08
Trail making task B time (ms)	Cognitive/Pschomotor	Trails	Smartphone	clinic	Baseline vs. 1st clinic visit	105	0.4	(0.22 0.55)	0.46
Trail making task B time (ms)	Cognitive/Pschomotor	Trails	Smartphone	home	1st vs 2nd at-home measurement	126	0.4	(0.24 0.54)	0.43
Trail making task B time (ms)	Cognitive/Pschomotor	Trails	Smartphone	home	2nd vs 3rd at-home measurement	123	0.39	(0.23 0.53)	0.48
Trail making task B time (ms)	Cognitive/Pschomotor	Trails	Smartphone	home	3rd vs 4th at-home measurement	120	0.54	(0.4 0.65)	0.53
Arm range of motion L (degrees) [mean]	Gait	2 minute walk	Research accel.	clinic	Baseline vs. 1st clinic visit	110	0.93	(0.9 0.95)	0.9
Arm range of motion L (degrees) [mean]	Gait	Dual task	Research accel.	clinic	Baseline vs. 1st clinic visit	110	0.95	(0.93 0.97)	0.93
Arm range of motion L (degrees) [std]	Gait	2 minute walk	Research accel.	clinic	Baseline vs. 1st clinic visit	110	0.34	(0.16 0.49)	0.5
Arm range of motion L (degrees) [std]	Gait	Dual task	Research accel.	clinic	Baseline vs. 1st clinic visit	110	0.39	(0.22 0.54)	0.56
Arm range of motion R (degrees) [mean]	Gait	2 minute walk	Research accel.	clinic	Baseline vs. 1st clinic visit	110	0.91	(0.86 0.93)	0.9
Arm range of motion R (degrees) [mean]	Gait	Dual task	Research accel.	clinic	Baseline vs. 1st clinic visit	110	0.93	(0.9 0.95)	0.94
Arm range of motion R (degrees) [std]	Gait	2 minute walk	Research accel.	clinic	Baseline vs. 1st clinic visit	110	0.26	(0.07 0.43)	0.59
Arm range of motion R (degrees) [std]	Gait	Dual task	Research accel.	clinic	Baseline vs. 1st clinic visit	110	0.53	(0.38 0.66)	0.78
Arm swing velocity L (degrees/s) [mean]	Gait	2 minute walk	Research accel.	clinic	Baseline vs. 1st clinic visit	110	0.91	(0.87 0.94)	0.86
Arm swing velocity L (degrees/s) [mean]	Gait	Dual task	Research accel.	clinic	Baseline vs. 1st clinic visit	110	0.93	(0.91 0.95)	0.9
Arm swing velocity L (degrees/s) [std]	Gait	2 minute walk	Research accel.	clinic	Baseline vs. 1st clinic visit	110	0.48	(0.32 0.61)	0.52
Arm swing velocity L (degrees/s) [std]	Gait	Dual task	Research accel.	clinic	Baseline vs. 1st clinic visit	110	0.51	(0.36 0.64)	0.66
Arm swing velocity R (degrees/s) [mean]	Gait	2 minute walk	Research accel.	clinic	Baseline vs. 1st clinic visit	110	0.9	(0.85 0.93)	0.87
Arm swing velocity R (degrees/s) [mean]	Gait	Dual task	Research accel.	clinic	Baseline vs. 1st clinic visit	110	0.91	(0.88 0.94)	0.9
Arm swing velocity R (degrees/s) [std]	Gait	2 minute walk	Research accel.	clinic	Baseline vs. 1st clinic visit	110	0.52	(0.36 0.64)	0.67
Arm swing velocity R (degrees/s) [std]	Gait	Dual task	Research accel.	clinic	Baseline vs. 1st clinic visit	110	0.59	(0.46 0.7)	0.73
Cadence L (steps/min) [mean]	Gait	2 minute walk	Research accel.	clinic	Baseline vs. 1st clinic visit	110	0.89	(0.84 0.92)	0.9
Cadence L (steps/min) [mean]	Gait	Dual task	Research accel.	clinic	Baseline vs. 1st clinic visit	110	0.86	(0.8 0.9)	0.85
Cadence L (steps/min) [std]	Gait	2 minute walk	Research accel.	clinic	Baseline vs. 1st clinic visit	110	0.4	(0.23 0.55)	0.57
Cadence L (steps/min) [std]	Gait	Dual task	Research accel.	clinic	Baseline vs. 1st clinic visit	110	0.49	(0.33 0.62)	0.56
Cadence R (steps/min) [mean]	Gait	2 minute walk	Research accel.	clinic	Baseline vs. 1st clinic visit	110	0.89	(0.84 0.92)	0.89
Cadence R (steps/min) [mean]	Gait	Dual task	Research accel.	clinic	Baseline vs. 1st clinic visit	110	0.86	(0.8 0.9)	0.85

Cadence R (steps/min) [std]	Gait	2 minute walk	Research accel.	clinic	Baseline vs. 1st clinic visit	110	0.45	(0.28 0.59)	0.58
Cadence R (steps/min) [std]	Gait	Dual task	Research accel.	clinic	Baseline vs. 1st clinic visit	110	0.5	(0.34 0.63)	0.52
Circumduction L (cm) [mean]	Gait	2 minute walk	Research accel.	clinic	Baseline vs. 1st clinic visit	110	0.75	(0.65 0.82)	0.76
Circumduction L (cm) [mean]	Gait	Dual task	Research accel.	clinic	Baseline vs. 1st clinic visit	110	0.76	(0.67 0.83)	0.8
Circumduction L (cm) [std]	Gait	2 minute walk	Research accel.	clinic	Baseline vs. 1st clinic visit	110	0.65	(0.52 0.74)	0.65
Circumduction L (cm) [std]	Gait	Dual task	Research accel.	clinic	Baseline vs. 1st clinic visit	110	0.62	(0.49 0.73)	0.67
Circumduction R (cm) [mean]	Gait	2 minute walk	Research accel.	clinic	Baseline vs. 1st clinic visit	110	0.72	(0.61 0.8)	0.75
Circumduction R (cm) [mean]	Gait	Dual task	Research accel.	clinic	Baseline vs. 1st clinic visit	110	0.77	(0.68 0.84)	0.81
Circumduction R (cm) [std]	Gait	2 minute walk	Research accel.	clinic	Baseline vs. 1st clinic visit	110	0.56	(0.42 0.68)	0.63
Circumduction R (cm) [std]	Gait	Dual task	Research accel.	clinic	Baseline vs. 1st clinic visit	110	0.59	(0.45 0.7)	0.66
Coronal range of motion (degrees) [mean]	Gait	2 minute walk	Research accel.	clinic	Baseline vs. 1st clinic visit	110	0.65	(0.53 0.75)	0.66
Coronal range of motion (degrees) [mean]	Gait	2 minute walk	Research accel.	clinic	Baseline vs. 1st clinic visit	110	0.9	(0.86 0.93)	0.88
Coronal range of motion (degrees) [mean]	Gait	Dual task	Research accel.	clinic	Baseline vs. 1st clinic visit	110	0.72	(0.62 0.8)	0.73
Coronal range of motion (degrees) [mean]	Gait	Dual task	Research accel.	clinic	Baseline vs. 1st clinic visit	110	0.91	(0.87 0.94)	0.91
Coronal range of motion (degrees) [std]	Gait	2 minute walk	Research accel.	clinic	Baseline vs. 1st clinic visit	110	0.5	(0.34 0.63)	0.47
Coronal range of motion (degrees) [std]	Gait	2 minute walk	Research accel.	clinic	Baseline vs. 1st clinic visit	110	0.67	(0.55 0.76)	0.7
Coronal range of motion (degrees) [std]	Gait	Dual task	Research accel.	clinic	Baseline vs. 1st clinic visit	110	0.6	(0.46 0.71)	0.57
Coronal range of motion (degrees) [std]	Gait	Dual task	Research accel.	clinic	Baseline vs. 1st clinic visit	110	0.56	(0.41 0.68)	0.7
Cycle duration L (s) [mean]	Gait	2 minute walk	Research accel.	clinic	Baseline vs. 1st clinic visit	110	0.88	(0.83 0.92)	0.89
Cycle duration L (s) [mean]	Gait	Dual task	Research accel.	clinic	Baseline vs. 1st clinic visit	110	0.82	(0.75 0.87)	0.85
Cycle duration L (s) [std]	Gait	2 minute walk	Research accel.	clinic	Baseline vs. 1st clinic visit	110	0.48	(0.32 0.61)	0.49
Cycle duration L (s) [std]	Gait	Dual task	Research accel.	clinic	Baseline vs. 1st clinic visit	110	0.48	(0.33 0.62)	0.55
Cycle duration R (s) [mean]	Gait	2 minute walk	Research accel.	clinic	Baseline vs. 1st clinic visit	110	0.88	(0.83 0.92)	0.89
Cycle duration R (s) [mean]	Gait	Dual task	Research accel.	clinic	Baseline vs. 1st clinic visit	110	0.82	(0.74 0.87)	0.84
Cycle duration R (s) [std]	Gait	2 minute walk	Research accel.	clinic	Baseline vs. 1st clinic visit	110	0.61	(0.48 0.72)	0.61
Cycle duration R (s) [std]	Gait	Dual task	Research accel.	clinic	Baseline vs. 1st clinic visit	110	0.53	(0.38 0.65)	0.53
DouBaselinee support L (%GCT) [mean]	Gait	2 minute walk	Research accel.	clinic	Baseline vs. 1st clinic visit	110	0.77	(0.67 0.83)	0.77
DouBaselinee support L (%GCT) [mean]	Gait	Dual task	Research accel.	clinic	Baseline vs. 1st clinic visit	110	0.73	(0.63 0.81)	0.78
DouBaselinee support L (%GCT) [std]	Gait	2 minute walk	Research accel.	clinic	Baseline vs. 1st clinic visit	110	0.31	(0.13 0.47)	0.46
DouBaselinee support L (%GCT) [std]	Gait	Dual task	Research accel.	clinic	Baseline vs. 1st clinic visit	110	0.52	(0.37 0.65)	0.49
DouBaselinee support R (%GCT) [mean]	Gait	2 minute walk	Research accel.	clinic	Baseline vs. 1st clinic visit	110	0.77	(0.67 0.83)	0.77
DouBaselinee support R (%GCT) [mean]	Gait	Dual task	Research accel.	clinic	Baseline vs. 1st clinic visit	110	0.73	(0.63 0.81)	0.79
DouBaselinee support R (%GCT) [std]	Gait	2 minute walk	Research accel.	clinic	Baseline vs. 1st clinic visit	110	0.4	(0.23 0.55)	0.38
DouBaselinee support R (%GCT) [std]	Gait	Dual task	Research accel.	clinic	Baseline vs. 1st clinic visit	110	0.6	(0.46 0.71)	0.55
Elevation at midswing L (cm) [mean]	Gait	2 minute walk	Research accel.	clinic	Baseline vs. 1st clinic visit	110	0.78	(0.69 0.84)	0.75
Elevation at midswing L (cm) [mean]	Gait	Dual task	Research accel.	clinic	Baseline vs. 1st clinic visit	110	0.84	(0.77 0.89)	0.8
Elevation at midswing L (cm) [std]	Gait	2 minute walk	Research accel.	clinic	Baseline vs. 1st clinic visit	110	0.71	(0.6 0.79)	0.71
Elevation at midswing L (cm) [std]	Gait	Dual task	Research accel.	clinic	Baseline vs. 1st clinic visit	110	0.61	(0.47 0.71)	0.7
Elevation at midswing R (cm) [mean]	Gait	2 minute walk	Research accel.	clinic	Baseline vs. 1st clinic visit	110	0.81	(0.73 0.87)	0.79
Elevation at midswing R (cm) [mean]	Gait	Dual task	Research accel.	clinic	Baseline vs. 1st clinic visit	110	0.83	(0.76 0.88)	0.79
Elevation at midswing R (cm) [std]	Gait	2 minute walk	Research accel.	clinic	Baseline vs. 1st clinic visit	110	0.61	(0.48 0.72)	0.65
Elevation at midswing R (cm) [std]	Gait	Dual task	Research accel.	clinic	Baseline vs. 1st clinic visit	110	0.57	(0.43 0.69)	0.58
Foot strike angle L (degrees) [mean]	Gait	2 minute walk	Research accel.	clinic	Baseline vs. 1st clinic visit	110	0.83	(0.76 0.88)	0.82
Foot strike angle L (degrees) [mean]	Gait	Dual task	Research accel.	clinic	Baseline vs. 1st clinic visit	110	0.87	(0.82 0.91)	0.83
Foot strike angle L (degrees) [std]	Gait	2 minute walk	Research accel.	clinic	Baseline vs. 1st clinic visit	110	0.46	(0.3 0.6)	0.49
Foot strike angle L (degrees) [std]	Gait	Dual task	Research accel.	clinic	Baseline vs. 1st clinic visit	110	0.45	(0.29 0.59)	0.49
Foot strike angle R (degrees) [mean]	Gait	2 minute walk	Research accel.	clinic	Baseline vs. 1st clinic visit	110	0.82	(0.74 0.87)	0.84
Foot strike angle R (degrees) [mean]	Gait	Dual task	Research accel.	clinic	Baseline vs. 1st clinic visit	110	0.87	(0.82 0.91)	0.86
Foot strike angle R (degrees) [std]	Gait	2 minute walk	Research accel.	clinic	Baseline vs. 1st clinic visit	110	0.48	(0.32 0.61)	0.5
Foot strike angle R (degrees) [std]	Gait	Dual task	Research accel.	clinic	Baseline vs. 1st clinic visit	110	0.28	(0.1 0.45)	0.63
Lateral step variability L (cm)	Gait	2 minute walk	Research accel.	clinic	Baseline vs. 1st clinic visit	110	0.64	(0.52 0.74)	0.63
Lateral step variability L (cm)	Gait	Dual task	Research accel.	clinic	Baseline vs. 1st clinic visit	110	0.7	(0.59 0.78)	0.69
Lateral step variability R (cm)	Gait	2 minute walk	Research accel.	clinic	Baseline vs. 1st clinic visit	110	0.64	(0.51 0.74)	0.63
Lateral step variability R (cm)	Gait	Dual task	Research accel.	clinic	Baseline vs. 1st clinic visit	110	0.59	(0.45 0.7)	0.62
Sagittal range of motion (degrees) [mean]	Gait	2 minute walk	Research accel.	clinic	Baseline vs. 1st clinic visit	110	0.4	(0.23 0.55)	0.4
Sagittal range of motion (degrees) [mean]	Gait	2 minute walk	Research accel.	clinic	Baseline vs. 1st clinic visit	110	0.63	(0.51 0.74)	0.72
Sagittal range of motion (degrees) [mean]	Gait	Dual task	Research accel.	clinic	Baseline vs. 1st clinic visit	110	0.49	(0.33 0.62)	0.54
Sagittal range of motion (degrees) [mean]	Gait	Dual task	Research accel.	clinic	Baseline vs. 1st clinic visit	110	0.7	(0.59 0.79)	0.76
Sagittal range of motion (degrees) [std]	Gait	2 minute walk	Research accel.	clinic	Baseline vs. 1st clinic visit	110	0.26	(0.07 0.43)	0.34
Sagittal range of motion (degrees) [std]	Gait	2 minute walk	Research accel.	clinic	Baseline vs. 1st clinic visit	110	0.2	(0.01 0.37)	0.47
Sagittal range of motion (degrees) [std]	Gait	Dual task	Research accel.	clinic	Baseline vs. 1st clinic visit	110	0.3	(0.12 0.46)	0.37
Sagittal range of motion (degrees) [std]	Gait	Dual task	Research accel.	clinic	Baseline vs. 1st clinic visit	110	0.22	(0.03 0.39)	0.54
Single limb support L (%GCT) [mean]	Gait	2 minute walk	Research accel.	clinic	Baseline vs. 1st clinic visit	110	0.72	(0.61 0.8)	0.76
Single limb support L (%GCT) [mean]	Gait	Dual task	Research accel.	clinic	Baseline vs. 1st clinic visit	110	0.78	(0.7 0.85)	0.79
Single limb support L (%GCT) [std]	Gait	2 minute walk	Research accel.	clinic	Baseline vs. 1st clinic visit	110	0.52	(0.36 0.64)	0.58
Single limb support L (%GCT) [std]	Gait	Dual task	Research accel.	clinic	Baseline vs. 1st clinic visit	110	0.69	(0.58 0.78)	0.65
Single limb support R (%GCT) [mean]	Gait	2 minute walk	Research accel.	clinic	Baseline vs. 1st clinic visit	110	0.79	(0.71 0.86)	0.76
Single limb support R (%GCT) [mean]	Gait	Dual task	Research accel.	clinic	Baseline vs. 1st clinic visit	110	0.63	(0.5 0.73)	0.77
Single limb support R (%GCT) [std]	Gait	2 minute walk	Research accel.	clinic	Baseline vs. 1st clinic visit	110	0.34	(0.16 0.49)	0.5
Single limb support R (%GCT) [std]	Gait	Dual task	Research accel.	clinic	Baseline vs. 1st clinic visit	110	0.59	(0.45 0.7)	0.54
Speed L (m/s) [mean]	Gait	2 minute walk	Research accel.	clinic	Baseline vs. 1st clinic visit	110	0.88	(0.82 0.91)	0.86
Speed L (m/s) [mean]	Gait	Dual task	Research accel.	clinic	Baseline vs. 1st clinic visit	110	0.88	(0.83 0.92)	0.86

Speed L (m/s) [std]	Gait	2 minute walk	Research accel.	clinic	Baseline vs. 1st clinic visit	110	0.28	(0.1 0.44)	0.41
Speed L (m/s) [std]	Gait	Dual task	Research accel.	clinic	Baseline vs. 1st clinic visit	110	0.46	(0.3 0.6)	0.51
Speed R (m/s) [mean]	Gait	2 minute walk	Research accel.	clinic	Baseline vs. 1st clinic visit	110	0.88	(0.84 0.92)	0.87
Speed R (m/s) [mean]	Gait	Dual task	Research accel.	clinic	Baseline vs. 1st clinic visit	110	0.88	(0.83 0.92)	0.86
Speed R (m/s) [std]	Gait	2 minute walk	Research accel.	clinic	Baseline vs. 1st clinic visit	110	0.45	(0.29 0.59)	0.48
Speed R (m/s) [std]	Gait	Dual task	Research accel.	clinic	Baseline vs. 1st clinic visit	110	0.51	(0.36 0.64)	0.5
Stance L (%GCT) [mean]	Gait	2 minute walk	Research accel.	clinic	Baseline vs. 1st clinic visit	110	0.79	(0.71 0.85)	0.77
Stance L (%GCT) [mean]	Gait	Dual task	Research accel.	clinic	Baseline vs. 1st clinic visit	110	0.64	(0.51 0.74)	0.78
Stance L (%GCT) [std]	Gait	2 minute walk	Research accel.	clinic	Baseline vs. 1st clinic visit	110	0.38	(0.21 0.53)	0.58
Stance L (%GCT) [std]	Gait	Dual task	Research accel.	clinic	Baseline vs. 1st clinic visit	110	0.56	(0.42 0.68)	0.55
Stance R (%GCT) [mean]	Gait	2 minute walk	Research accel.	clinic	Baseline vs. 1st clinic visit	110	0.72	(0.62 0.8)	0.76
Stance R (%GCT) [mean]	Gait	Dual task	Research accel.	clinic	Baseline vs. 1st clinic visit	110	0.78	(0.7 0.85)	0.79
Stance R (%GCT) [std]	Gait	2 minute walk	Research accel.	clinic	Baseline vs. 1st clinic visit	110	0.44	(0.27 0.58)	0.61
Stance R (%GCT) [std]	Gait	Dual task	Research accel.	clinic	Baseline vs. 1st clinic visit	110	0.66	(0.54 0.76)	0.61
Step duration L (s) [mean]	Gait	2 minute walk	Research accel.	clinic	Baseline vs. 1st clinic visit	110	0.87	(0.82 0.91)	0.89
Step duration L (s) [mean]	Gait	Dual task	Research accel.	clinic	Baseline vs. 1st clinic visit	110	0.75	(0.66 0.82)	0.84
Step duration L (s) [std]	Gait	2 minute walk	Research accel.	clinic	Baseline vs. 1st clinic visit	110	0.49	(0.33 0.62)	0.48
Step duration L (s) [std]	Gait	Dual task	Research accel.	clinic	Baseline vs. 1st clinic visit	110	0.51	(0.35 0.63)	0.52
Step duration R (s) [mean]	Gait	2 minute walk	Research accel.	clinic	Baseline vs. 1st clinic visit	110	0.87	(0.81 0.91)	0.88
Step duration R (s) [mean]	Gait	Dual task	Research accel.	clinic	Baseline vs. 1st clinic visit	110	0.83	(0.76 0.88)	0.85
Step duration R (s) [std]	Gait	2 minute walk	Research accel.	clinic	Baseline vs. 1st clinic visit	110	0.3	(0.12 0.46)	0.34
Step duration R (s) [std]	Gait	Dual task	Research accel.	clinic	Baseline vs. 1st clinic visit	110	0.53	(0.39 0.66)	0.47
Stride length L (m) [mean]	Gait	2 minute walk	Research accel.	clinic	Baseline vs. 1st clinic visit	110	0.91	(0.87 0.94)	0.92
Stride length L (m) [mean]	Gait	Dual task	Research accel.	clinic	Baseline vs. 1st clinic visit	110	0.92	(0.88 0.94)	0.91
Stride length L (m) [std]	Gait	2 minute walk	Research accel.	clinic	Baseline vs. 1st clinic visit	110	0.19	(0. 0.36)	0.4
Stride length L (m) [std]	Gait	Dual task	Research accel.	clinic	Baseline vs. 1st clinic visit	110	0.49	(0.33 0.62)	0.43
Stride length R (m) [mean]	Gait	2 minute walk	Research accel.	clinic	Baseline vs. 1st clinic visit	110	0.92	(0.88 0.94)	0.93
Stride length R (m) [mean]	Gait	Dual task	Research accel.	clinic	Baseline vs. 1st clinic visit	110	0.92	(0.88 0.94)	0.91
Stride length R (m) [std]	Gait	2 minute walk	Research accel.	clinic	Baseline vs. 1st clinic visit	110	0.33	(0.15 0.48)	0.36
Stride length R (m) [std]	Gait	Dual task	Research accel.	clinic	Baseline vs. 1st clinic visit	110	0.52	(0.36 0.64)	0.44
Swing L (%GCT) [mean]	Gait	2 minute walk	Research accel.	clinic	Baseline vs. 1st clinic visit	110	0.79	(0.71 0.85)	0.77
Swing L (%GCT) [mean]	Gait	Dual task	Research accel.	clinic	Baseline vs. 1st clinic visit	110	0.64	(0.51 0.74)	0.78
Swing L (%GCT) [std]	Gait	2 minute walk	Research accel.	clinic	Baseline vs. 1st clinic visit	110	0.38	(0.21 0.53)	0.58
Swing L (%GCT) [std]	Gait	Dual task	Research accel.	clinic	Baseline vs. 1st clinic visit	110	0.56	(0.42 0.68)	0.55
Swing R (%GCT) [mean]	Gait	2 minute walk	Research accel.	clinic	Baseline vs. 1st clinic visit	110	0.72	(0.62 0.8)	0.76
Swing R (%GCT) [mean]	Gait	Dual task	Research accel.	clinic	Baseline vs. 1st clinic visit	110	0.78	(0.7 0.85)	0.79
Swing R (%GCT) [std]	Gait	2 minute walk	Research accel.	clinic	Baseline vs. 1st clinic visit	110	0.44	(0.27 0.58)	0.61
Swing R (%GCT) [std]	Gait	Dual task	Research accel.	clinic	Baseline vs. 1st clinic visit	110	0.66	(0.54 0.76)	0.61
Terminal douBaselinee support L (%GCT) [mean]	Gait	2 minute walk	Research accel.	clinic	Baseline vs. 1st clinic visit	110	0.76	(0.66 0.83)	0.76
Terminal douBaselinee support L (%GCT) [mean]	Gait	Dual task	Research accel.	clinic	Baseline vs. 1st clinic visit	110	0.41	(0.24 0.55)	0.75
Terminal douBaselinee support L (%GCT) [std]	Gait	2 minute walk	Research accel.	clinic	Baseline vs. 1st clinic visit	110	0.49	(0.33 0.62)	0.53
Terminal douBaselinee support L (%GCT) [std]	Gait	Dual task	Research accel.	clinic	Baseline vs. 1st clinic visit	110	0.57	(0.43 0.68)	0.6
Terminal douBaselinee support R (%GCT) [mean]	Gait	2 minute walk	Research accel.	clinic	Baseline vs. 1st clinic visit	110	0.76	(0.66 0.83)	0.73
Terminal douBaselinee support R (%GCT) [mean]	Gait	Dual task	Research accel.	clinic	Baseline vs. 1st clinic visit	110	0.81	(0.74 0.87)	0.81
Terminal douBaselinee support R (%GCT) [std]	Gait	2 minute walk	Research accel.	clinic	Baseline vs. 1st clinic visit	110	0.45	(0.29 0.59)	0.46
Terminal douBaselinee support R (%GCT) [std]	Gait	Dual task	Research accel.	clinic	Baseline vs. 1st clinic visit	110	0.6	(0.47 0.71)	0.53
Toe off angle L (degrees) [mean]	Gait	2 minute walk	Research accel.	clinic	Baseline vs. 1st clinic visit	110	0.76	(0.66 0.83)	0.75
Toe off angle L (degrees) [mean]	Gait	Dual task	Research accel.	clinic	Baseline vs. 1st clinic visit	110	0.79	(0.71 0.85)	0.79
Toe off angle L (degrees) [std]	Gait	2 minute walk	Research accel.	clinic	Baseline vs. 1st clinic visit	110	0.52	(0.37 0.64)	0.66
Toe off angle L (degrees) [std]	Gait	Dual task	Research accel.	clinic	Baseline vs. 1st clinic visit	110	0.66	(0.54 0.76)	0.53
Toe off angle R (degrees) [mean]	Gait	2 minute walk	Research accel.	clinic	Baseline vs. 1st clinic visit	110	0.8	(0.72 0.86)	0.83
Toe off angle R (degrees) [mean]	Gait	Dual task	Research accel.	clinic	Baseline vs. 1st clinic visit	110	0.84	(0.77 0.89)	0.82
Toe off angle R (degrees) [std]	Gait	2 minute walk	Research accel.	clinic	Baseline vs. 1st clinic visit	110	0.46	(0.3 0.6)	0.56
Toe off angle R (degrees) [std]	Gait	Dual task	Research accel.	clinic	Baseline vs. 1st clinic visit	110	0.22	(0.03 0.39)	0.48
Toe out angle L (degrees) [mean]	Gait	2 minute walk	Research accel.	clinic	Baseline vs. 1st clinic visit	110	0.76	(0.66 0.83)	0.72
Toe out angle L (degrees) [mean]	Gait	Dual task	Research accel.	clinic	Baseline vs. 1st clinic visit	110	0.74	(0.64 0.82)	0.7
Toe out angle L (degrees) [std]	Gait	2 minute walk	Research accel.	clinic	Baseline vs. 1st clinic visit	110	0.56	(0.42 0.68)	0.6
Toe out angle L (degrees) [std]	Gait	Dual task	Research accel.	clinic	Baseline vs. 1st clinic visit	110	0.72	(0.62 0.8)	0.7
Toe out angle R (degrees) [mean]	Gait	2 minute walk	Research accel.	clinic	Baseline vs. 1st clinic visit	110	0.52	(0.37 0.64)	0.58
Toe out angle R (degrees) [mean]	Gait	Dual task	Research accel.	clinic	Baseline vs. 1st clinic visit	110	0.53	(0.38 0.65)	0.61
Toe out angle R (degrees) [std]	Gait	2 minute walk	Research accel.	clinic	Baseline vs. 1st clinic visit	110	0.59	(0.45 0.7)	0.63
Toe out angle R (degrees) [std]	Gait	Dual task	Research accel.	clinic	Baseline vs. 1st clinic visit	110	0.67	(0.55 0.76)	0.62
Transverse range of motion (degrees) [mean]	Gait	2 minute walk	Research accel.	clinic	Baseline vs. 1st clinic visit	110	0.72	(0.61 0.8)	0.69
Transverse range of motion (degrees) [mean]	Gait	2 minute walk	Research accel.	clinic	Baseline vs. 1st clinic visit	110	0.77	(0.69 0.84)	0.87
Transverse range of motion (degrees) [mean]	Gait	Dual task	Research accel.	clinic	Baseline vs. 1st clinic visit	110	0.76	(0.67 0.83)	0.75
Transverse range of motion (degrees) [mean]	Gait	Dual task	Research accel.	clinic	Baseline vs. 1st clinic visit	110	0.89	(0.85 0.93)	0.92
Transverse range of motion (degrees) [std]	Gait	2 minute walk	Research accel.	clinic	Baseline vs. 1st clinic visit	110	0.69	(0.58 0.78)	0.7
Transverse range of motion (degrees) [std]	Gait	2 minute walk	Research accel.	clinic	Baseline vs. 1st clinic visit	110	0.45	(0.29 0.59)	0.68
Transverse range of motion (degrees) [std]	Gait	Dual task	Research accel.	clinic	Baseline vs. 1st clinic visit	110	0.42	(0.26 0.57)	0.49
Transverse range of motion (degrees) [std]	Gait	Dual task	Research accel.	clinic	Baseline vs. 1st clinic visit	110	0.52	(0.36 0.64)	0.78
Cadence (steps/min) [mean]	Gait	1 minute walk	Smartphone	clinic	Baseline vs. 1st clinic visit	72	0.85	(0.78 0.91)	0.82
Cadence (steps/min) [mean]	Gait	1 minute walk	Smartphone	home	1st vs 2nd at-home measurement	122	0.84	(0.78 0.88)	0.85

Cadence (steps/min) [mean]	Gait	1 minute walk	Smartphone	home	2nd vs 3rd at-home measurement	117	0.82	(0.75 0.87)	0.84
Cadence (steps/min) [mean]	Gait	1 minute walk	Smartphone	home	3rd vs 4th at-home measurement	113	0.86	(0.8 0.9)	0.8
Cadence (steps/min) [std]	Gait	1 minute walk	Smartphone	clinic	Baseline vs. 1st clinic visit	70	0.09	(-0.15 0.32)	0.22
Cadence (steps/min) [std]	Gait	1 minute walk	Smartphone	home	1st vs 2nd at-home measurement	120	0.39	(0.23 0.53)	0.37
Cadence (steps/min) [std]	Gait	1 minute walk	Smartphone	home	2nd vs 3rd at-home measurement	117	0.3	(0.13 0.46)	0.31
Cadence (steps/min) [std]	Gait	1 minute walk	Smartphone	home	3rd vs 4th at-home measurement	109	0.4	(0.23 0.54)	0.34
DouBaselinee support (s) [mean]	Gait	1 minute walk	Smartphone	clinic	Baseline vs. 1st clinic visit	72	0.85	(0.78 0.91)	0.82
DouBaselinee support (s) [mean]	Gait	1 minute walk	Smartphone	home	1st vs 2nd at-home measurement	122	0.83	(0.77 0.88)	0.85
DouBaselinee support (s) [mean]	Gait	1 minute walk	Smartphone	home	2nd vs 3rd at-home measurement	117	0.84	(0.78 0.89)	0.85
DouBaselinee support (s) [mean]	Gait	1 minute walk	Smartphone	home	3rd vs 4th at-home measurement	113	0.87	(0.81 0.91)	0.83
DouBaselinee support (s) [std]	Gait	1 minute walk	Smartphone	clinic	Baseline vs. 1st clinic visit	68	0.28	(0.05 0.49)	0.29
DouBaselinee support (s) [std]	Gait	1 minute walk	Smartphone	home	1st vs 2nd at-home measurement	122	0.29	(0.12 0.45)	0.29
DouBaselinee support (s) [std]	Gait	1 minute walk	Smartphone	home	2nd vs 3rd at-home measurement	117	0.11	(-0.08 0.28)	0.08
DouBaselinee support (s) [std]	Gait	1 minute walk	Smartphone	home	3rd vs 4th at-home measurement	109	0.38	(0.21 0.53)	0.42
DouBaselinee support (s) asymmetry	Gait	1 minute walk	Smartphone	clinic	Baseline vs. 1st clinic visit	67	0.08	(-0.16 0.31)	-0.02
DouBaselinee support (s) asymmetry	Gait	1 minute walk	Smartphone	home	1st vs 2nd at-home measurement	121	0.37	(0.21 0.51)	0.41
DouBaselinee support (s) asymmetry	Gait	1 minute walk	Smartphone	home	2nd vs 3rd at-home measurement	115	0.43	(0.27 0.57)	0.5
DouBaselinee support (s) asymmetry	Gait	1 minute walk	Smartphone	home	3rd vs 4th at-home measurement	107	0.57	(0.42 0.68)	0.58
Gait speed (m/s) [mean]	Gait	1 minute walk	Smartphone	clinic	Baseline vs. 1st clinic visit	71	0.76	(0.65 0.85)	0.65
Gait speed (m/s) [mean]	Gait	1 minute walk	Smartphone	home	1st vs 2nd at-home measurement	121	0.77	(0.69 0.83)	0.76
Gait speed (m/s) [mean]	Gait	1 minute walk	Smartphone	home	2nd vs 3rd at-home measurement	116	0.78	(0.7 0.85)	0.77
Gait speed (m/s) [mean]	Gait	1 minute walk	Smartphone	home	3rd vs 4th at-home measurement	112	0.71	(0.6 0.79)	0.71
Gait speed (m/s) [std]	Gait	1 minute walk	Smartphone	clinic	Baseline vs. 1st clinic visit	70	0.15	(-0.08 0.37)	0.25
Gait speed (m/s) [std]	Gait	1 minute walk	Smartphone	home	1st vs 2nd at-home measurement	121	0.09	(-0.08 0.27)	0.06
Gait speed (m/s) [std]	Gait	1 minute walk	Smartphone	home	2nd vs 3rd at-home measurement	116	0.22	(0.04 0.39)	0.27
Gait speed (m/s) [std]	Gait	1 minute walk	Smartphone	home	3rd vs 4th at-home measurement	109	0.34	(0.17 0.5)	0.31
Initial douBaselinee support (s) [mean]	Gait	1 minute walk	Smartphone	clinic	Baseline vs. 1st clinic visit	72	0.82	(0.72 0.88)	0.77
Initial douBaselinee support (s) [mean]	Gait	1 minute walk	Smartphone	home	1st vs 2nd at-home measurement	122	0.78	(0.7 0.84)	0.82
Initial douBaselinee support (s) [mean]	Gait	1 minute walk	Smartphone	home	2nd vs 3rd at-home measurement	117	0.82	(0.74 0.87)	0.84
Initial douBaselinee support (s) [mean]	Gait	1 minute walk	Smartphone	home	3rd vs 4th at-home measurement	113	0.83	(0.76 0.88)	0.79
Initial douBaselinee support (s) [std]	Gait	1 minute walk	Smartphone	clinic	Baseline vs. 1st clinic visit	67	0.12	(-0.12 0.35)	0.2
Initial douBaselinee support (s) [std]	Gait	1 minute walk	Smartphone	home	1st vs 2nd at-home measurement	120	0.42	(0.26 0.56)	0.42
Initial douBaselinee support (s) [std]	Gait	1 minute walk	Smartphone	home	2nd vs 3rd at-home measurement	115	0.45	(0.29 0.58)	0.39
Initial douBaselinee support (s) [std]	Gait	1 minute walk	Smartphone	home	3rd vs 4th at-home measurement	107	0.51	(0.36 0.64)	0.5
Initial douBaselinee support (s) asymmetry	Gait	1 minute walk	Smartphone	clinic	Baseline vs. 1st clinic visit	68	0.73	(0.6 0.82)	0.53
Initial douBaselinee support (s) asymmetry	Gait	1 minute walk	Smartphone	home	1st vs 2nd at-home measurement	120	0.34	(0.17 0.49)	0.37
Initial douBaselinee support (s) asymmetry	Gait	1 minute walk	Smartphone	home	2nd vs 3rd at-home measurement	114	0.43	(0.27 0.57)	0.37
Initial douBaselinee support (s) asymmetry	Gait	1 minute walk	Smartphone	home	3rd vs 4th at-home measurement	107	0.47	(0.31 0.6)	0.38
Single limb support (s) [mean]	Gait	1 minute walk	Smartphone	clinic	Baseline vs. 1st clinic visit	72	0.82	(0.73 0.88)	0.77
Single limb support (s) [mean]	Gait	1 minute walk	Smartphone	home	1st vs 2nd at-home measurement	122	0.8	(0.73 0.86)	0.83
Single limb support (s) [mean]	Gait	1 minute walk	Smartphone	home	2nd vs 3rd at-home measurement	117	0.84	(0.77 0.88)	0.84
Single limb support (s) [mean]	Gait	1 minute walk	Smartphone	home	3rd vs 4th at-home measurement	113	0.81	(0.74 0.87)	0.79
Single limb support (s) [std]	Gait	1 minute walk	Smartphone	clinic	Baseline vs. 1st clinic visit	64	0.23	(-0.02 0.45)	0.34
Single limb support (s) [std]	Gait	1 minute walk	Smartphone	home	1st vs 2nd at-home measurement	120	0.3	(0.13 0.45)	0.36
Single limb support (s) [std]	Gait	1 minute walk	Smartphone	home	2nd vs 3rd at-home measurement	117	0.2	(0.02 0.37)	0.24
Single limb support (s) [std]	Gait	1 minute walk	Smartphone	home	3rd vs 4th at-home measurement	109	0.32	(0.14 0.48)	0.4
Single limb support (s) asymmetry	Gait	1 minute walk	Smartphone	clinic	Baseline vs. 1st clinic visit	71	0.38	(0.17 0.57)	0.37
Single limb support (s) asymmetry	Gait	1 minute walk	Smartphone	home	1st vs 2nd at-home measurement	121	0.51	(0.36 0.63)	0.51
Single limb support (s) asymmetry	Gait	1 minute walk	Smartphone	home	2nd vs 3rd at-home measurement	117	0.25	(0.07 0.41)	0.37
Single limb support (s) asymmetry	Gait	1 minute walk	Smartphone	home	3rd vs 4th at-home measurement	108	0.44	(0.28 0.58)	0.43
Stance (%GCT) asymmetry	Gait	1 minute walk	Smartphone	clinic	Baseline vs. 1st clinic visit	72	0.42	(0.22 0.6)	0.42
Stance (%GCT) asymmetry	Gait	1 minute walk	Smartphone	home	1st vs 2nd at-home measurement	121	0.56	(0.43 0.67)	0.48
Stance (%GCT) asymmetry	Gait	1 minute walk	Smartphone	home	2nd vs 3rd at-home measurement	117	0.37	(0.2 0.52)	0.39
Stance (%GCT) asymmetry	Gait	1 minute walk	Smartphone	home	3rd vs 4th at-home measurement	111	0.42	(0.25 0.56)	0.45
Stance (s) [mean]	Gait	1 minute walk	Smartphone	clinic	Baseline vs. 1st clinic visit	72	0.86	(0.78 0.91)	0.82
Stance (s) [mean]	Gait	1 minute walk	Smartphone	home	1st vs 2nd at-home measurement	122	0.82	(0.75 0.87)	0.85
Stance (s) [mean]	Gait	1 minute walk	Smartphone	home	2nd vs 3rd at-home measurement	117	0.85	(0.79 0.89)	0.86
Stance (s) [mean]	Gait	1 minute walk	Smartphone	home	3rd vs 4th at-home measurement	113	0.85	(0.79 0.89)	0.81
Stance (s) [std]	Gait	1 minute walk	Smartphone	clinic	Baseline vs. 1st clinic visit	70	0.16	(-0.07 0.38)	0.25
Stance (s) [std]	Gait	1 minute walk	Smartphone	home	1st vs 2nd at-home measurement	121	0.32	(0.15 0.47)	0.39
Stance (s) [std]	Gait	1 minute walk	Smartphone	home	2nd vs 3rd at-home measurement	118	0.3	(0.13 0.46)	0.29
Stance (s) [std]	Gait	1 minute walk	Smartphone	home	3rd vs 4th at-home measurement	112	0.29	(0.11 0.45)	0.35
Stance (s) asymmetry	Gait	1 minute walk	Smartphone	clinic	Baseline vs. 1st clinic visit	71	0.36	(0.14 0.55)	0.39
Stance (s) asymmetry	Gait	1 minute walk	Smartphone	home	1st vs 2nd at-home measurement	119	0.39	(0.23 0.54)	0.42
Stance (s) asymmetry	Gait	1 minute walk	Smartphone	home	2nd vs 3rd at-home measurement	117	0.27	(0.1 0.43)	0.36
Stance (s) asymmetry	Gait	1 minute walk	Smartphone	home	3rd vs 4th at-home measurement	108	0.46	(0.3 0.6)	0.46
Step duration (s) [mean]	Gait	1 minute walk	Smartphone	clinic	Baseline vs. 1st clinic visit	72	0.85	(0.77 0.9)	0.8
Step duration (s) [mean]	Gait	1 minute walk	Smartphone	home	1st vs 2nd at-home measurement	122	0.81	(0.73 0.86)	0.84
Step duration (s) [mean]	Gait	1 minute walk	Smartphone	home	2nd vs 3rd at-home measurement	117	0.84	(0.77 0.88)	0.84
Step duration (s) [mean]	Gait	1 minute walk	Smartphone	home	3rd vs 4th at-home measurement	113	0.81	(0.74 0.87)	0.78
Step duration (s) [std]	Gait	1 minute walk	Smartphone	clinic	Baseline vs. 1st clinic visit	67	0.17	(-0.07 0.4)	0.29
Step duration (s) [std]	Gait	1 minute walk	Smartphone	home	1st vs 2nd at-home measurement	121	0.41	(0.25 0.55)	0.43

Step duration (s) [std]	Gait	1 minute walk	Smartphone	home	2nd vs 3rd at-home measurement	116	0.31	(0.14 0.47)	0.34
Step duration (s) [std]	Gait	1 minute walk	Smartphone	home	3rd vs 4th at-home measurement	111	0.36	(0.18 0.51)	0.43
Step duration (s) asymmetry	Gait	1 minute walk	Smartphone	clinic	Baseline vs. 1st clinic visit	72	0.43	(0.22 0.6)	0.37
Step duration (s) asymmetry	Gait	1 minute walk	Smartphone	home	1st vs 2nd at-home measurement	121	0.47	(0.32 0.59)	0.41
Step duration (s) asymmetry	Gait	1 minute walk	Smartphone	home	2nd vs 3rd at-home measurement	117	0.41	(0.24 0.55)	0.43
Step duration (s) asymmetry	Gait	1 minute walk	Smartphone	home	3rd vs 4th at-home measurement	109	0.46	(0.3 0.6)	0.42
Step length (m) [mean]	Gait	1 minute walk	Smartphone	clinic	Baseline vs. 1st clinic visit	74	0.67	(0.52 0.78)	0.69
Step length (m) [mean]	Gait	1 minute walk	Smartphone	home	1st vs 2nd at-home measurement	121	0.7	(0.59 0.78)	0.72
Step length (m) [mean]	Gait	1 minute walk	Smartphone	home	2nd vs 3rd at-home measurement	117	0.74	(0.64 0.81)	0.75
Step length (m) [mean]	Gait	1 minute walk	Smartphone	home	3rd vs 4th at-home measurement	112	0.73	(0.63 0.81)	0.74
Step length (m) [std]	Gait	1 minute walk	Smartphone	clinic	Baseline vs. 1st clinic visit	73	0.24	(0.01 0.44)	0.32
Step length (m) [std]	Gait	1 minute walk	Smartphone	home	1st vs 2nd at-home measurement	120	0.44	(0.29 0.58)	0.4
Step length (m) [std]	Gait	1 minute walk	Smartphone	home	2nd vs 3rd at-home measurement	113	0.38	(0.21 0.53)	0.33
Step length (m) [std]	Gait	1 minute walk	Smartphone	home	3rd vs 4th at-home measurement	108	0.53	(0.38 0.65)	0.51
Step length (m) asymmetry	Gait	1 minute walk	Smartphone	clinic	Baseline vs. 1st clinic visit	73	0.4	(0.19 0.57)	0.51
Step length (m) asymmetry	Gait	1 minute walk	Smartphone	home	1st vs 2nd at-home measurement	120	0.38	(0.22 0.53)	0.39
Step length (m) asymmetry	Gait	1 minute walk	Smartphone	home	2nd vs 3rd at-home measurement	113	0.52	(0.38 0.65)	0.48
Step length (m) asymmetry	Gait	1 minute walk	Smartphone	home	3rd vs 4th at-home measurement	107	0.39	(0.22 0.54)	0.32
Stride duration (s) [mean]	Gait	1 minute walk	Smartphone	clinic	Baseline vs. 1st clinic visit	72	0.86	(0.79 0.91)	0.82
Stride duration (s) [mean]	Gait	1 minute walk	Smartphone	home	1st vs 2nd at-home measurement	122	0.83	(0.77 0.88)	0.86
Stride duration (s) [mean]	Gait	1 minute walk	Smartphone	home	2nd vs 3rd at-home measurement	117	0.83	(0.76 0.88)	0.85
Stride duration (s) [mean]	Gait	1 minute walk	Smartphone	home	3rd vs 4th at-home measurement	113	0.83	(0.77 0.88)	0.8
Stride duration (s) [std]	Gait	1 minute walk	Smartphone	clinic	Baseline vs. 1st clinic visit	67	0.08	(-0.16 0.31)	0.18
Stride duration (s) [std]	Gait	1 minute walk	Smartphone	home	1st vs 2nd at-home measurement	120	0.4	(0.24 0.54)	0.37
Stride duration (s) [std]	Gait	1 minute walk	Smartphone	home	2nd vs 3rd at-home measurement	117	0.35	(0.18 0.5)	0.36
Stride duration (s) [std]	Gait	1 minute walk	Smartphone	home	3rd vs 4th at-home measurement	109	0.37	(0.2 0.52)	0.39
Stride duration (s) asymmetry	Gait	1 minute walk	Smartphone	clinic	Baseline vs. 1st clinic visit	71	-0.18	(-0.39 0.06)	-0.12
Stride duration (s) asymmetry	Gait	1 minute walk	Smartphone	home	1st vs 2nd at-home measurement	120	0.36	(0.2 0.51)	0.39
Stride duration (s) asymmetry	Gait	1 minute walk	Smartphone	home	2nd vs 3rd at-home measurement	116	0.38	(0.21 0.52)	0.38
Stride duration (s) asymmetry	Gait	1 minute walk	Smartphone	home	3rd vs 4th at-home measurement	108	0.51	(0.36 0.64)	0.6
Stride length (m) [mean]	Gait	1 minute walk	Smartphone	clinic	Baseline vs. 1st clinic visit	74	0.68	(0.53 0.78)	0.68
Stride length (m) [mean]	Gait	1 minute walk	Smartphone	home	1st vs 2nd at-home measurement	121	0.71	(0.6 0.79)	0.73
Stride length (m) [mean]	Gait	1 minute walk	Smartphone	home	2nd vs 3rd at-home measurement	117	0.73	(0.64 0.81)	0.74
Stride length (m) [mean]	Gait	1 minute walk	Smartphone	home	3rd vs 4th at-home measurement	112	0.75	(0.65 0.82)	0.76
Stride length (m) [std]	Gait	1 minute walk	Smartphone	clinic	Baseline vs. 1st clinic visit	69	0.16	(-0.08 0.38)	0.23
Stride length (m) [std]	Gait	1 minute walk	Smartphone	home	1st vs 2nd at-home measurement	121	0.14	(-0.03 0.31)	0.1
Stride length (m) [std]	Gait	1 minute walk	Smartphone	home	2nd vs 3rd at-home measurement	116	0.3	(0.12 0.45)	0.27
Stride length (m) [std]	Gait	1 minute walk	Smartphone	home	3rd vs 4th at-home measurement	110	0.18	(-0. 0.36)	0.22
Stride length (m) asymmetry	Gait	1 minute walk	Smartphone	clinic	Baseline vs. 1st clinic visit	71	-0.08	(-0.3 0.16)	0.09
Stride length (m) asymmetry	Gait	1 minute walk	Smartphone	home	1st vs 2nd at-home measurement	120	0.24	(0.06 0.4)	0.3
Stride length (m) asymmetry	Gait	1 minute walk	Smartphone	home	2nd vs 3rd at-home measurement	115	0.32	(0.15 0.47)	0.38
Stride length (m) asymmetry	Gait	1 minute walk	Smartphone	home	3rd vs 4th at-home measurement	110	0.51	(0.35 0.63)	0.53
Swing (s) [mean]	Gait	1 minute walk	Smartphone	clinic	Baseline vs. 1st clinic visit	72	0.84	(0.76 0.9)	0.8
Swing (s) [mean]	Gait	1 minute walk	Smartphone	home	1st vs 2nd at-home measurement	122	0.82	(0.76 0.87)	0.86
Swing (s) [mean]	Gait	1 minute walk	Smartphone	home	2nd vs 3rd at-home measurement	117	0.84	(0.78 0.89)	0.85
Swing (s) [mean]	Gait	1 minute walk	Smartphone	home	3rd vs 4th at-home measurement	113	0.82	(0.75 0.87)	0.81
Swing (s) [std]	Gait	1 minute walk	Smartphone	clinic	Baseline vs. 1st clinic visit	68	0.36	(0.13 0.55)	0.46
Swing (s) [std]	Gait	1 minute walk	Smartphone	home	1st vs 2nd at-home measurement	121	0.35	(0.19 0.5)	0.45
Swing (s) [std]	Gait	1 minute walk	Smartphone	home	2nd vs 3rd at-home measurement	117	0.28	(0.1 0.44)	0.38
Swing (s) [std]	Gait	1 minute walk	Smartphone	home	3rd vs 4th at-home measurement	109	0.3	(0.12 0.46)	0.37
Swing (s) asymmetry	Gait	1 minute walk	Smartphone	clinic	Baseline vs. 1st clinic visit	71	0.28	(0.06 0.48)	0.31
Swing (s) asymmetry	Gait	1 minute walk	Smartphone	home	1st vs 2nd at-home measurement	121	0.44	(0.29 0.58)	0.41
Swing (s) asymmetry	Gait	1 minute walk	Smartphone	home	2nd vs 3rd at-home measurement	117	0.32	(0.15 0.47)	0.39
Swing (s) asymmetry	Gait	1 minute walk	Smartphone	home	3rd vs 4th at-home measurement	111	0.42	(0.25 0.56)	0.44
Terminal douBaselinee support (s) [mean]	Gait	1 minute walk	Smartphone	clinic	Baseline vs. 1st clinic visit	72	0.84	(0.76 0.9)	0.82
Terminal douBaselinee support (s) [mean]	Gait	1 minute walk	Smartphone	home	1st vs 2nd at-home measurement	122	0.83	(0.76 0.88)	0.85
Terminal douBaselinee support (s) [mean]	Gait	1 minute walk	Smartphone	home	2nd vs 3rd at-home measurement	117	0.85	(0.79 0.89)	0.85
Terminal douBaselinee support (s) [mean]	Gait	1 minute walk	Smartphone	home	3rd vs 4th at-home measurement	112	0.86	(0.81 0.9)	0.83
Terminal douBaselinee support (s) [std]	Gait	1 minute walk	Smartphone	clinic	Baseline vs. 1st clinic visit	67	0.55	(0.35 0.69)	0.61
Terminal douBaselinee support (s) [std]	Gait	1 minute walk	Smartphone	home	1st vs 2nd at-home measurement	118	0.47	(0.32 0.6)	0.44
Terminal douBaselinee support (s) [std]	Gait	1 minute walk	Smartphone	home	2nd vs 3rd at-home measurement	114	0.39	(0.22 0.53)	0.31
Terminal douBaselinee support (s) [std]	Gait	1 minute walk	Smartphone	home	3rd vs 4th at-home measurement	106	0.58	(0.44 0.69)	0.56
Terminal douBaselinee support (s) asymmetry	Gait	1 minute walk	Smartphone	clinic	Baseline vs. 1st clinic visit	67	0.72	(0.58 0.82)	0.59
Terminal douBaselinee support (s) asymmetry	Gait	1 minute walk	Smartphone	home	1st vs 2nd at-home measurement	117	0.46	(0.3 0.59)	0.45
Terminal douBaselinee support (s) asymmetry	Gait	1 minute walk	Smartphone	home	2nd vs 3rd at-home measurement	113	0.31	(0.13 0.46)	0.24
Terminal douBaselinee support (s) asymmetry	Gait	1 minute walk	Smartphone	home	3rd vs 4th at-home measurement	106	0.52	(0.37 0.65)	0.48
Turn duration (s) [mean]	Gait	1 minute walk	Smartphone	clinic	Baseline vs. 1st clinic visit	113	0.5	(0.34 0.62)	0.55
Turn duration (s) [mean]	Gait	1 minute walk	Smartphone	home	1st vs 2nd at-home measurement	126	0.43	(0.28 0.56)	0.47
Turn duration (s) [mean]	Gait	1 minute walk	Smartphone	home	2nd vs 3rd at-home measurement	120	0.43	(0.27 0.56)	0.42
Turn duration (s) [mean]	Gait	1 minute walk	Smartphone	home	3rd vs 4th at-home measurement	118	0.51	(0.36 0.63)	0.46
Arm swing (degrees) [mean]	Gait	1 minute walk	Smartwatch	clinic	Baseline vs. 1st clinic visit	74	0.84	(0.76 0.9)	0.81
Arm swing (degrees) [mean]	Gait	1 minute walk	Smartwatch	home	1st vs 2nd at-home measurement	122	0.77	(0.68 0.83)	0.81

Arm swing (degrees) [mean]	Gait	1 minute walk	Smartwatch	home	2nd vs 3rd at-home measurement	118	0.78	(0.7 0.85)	0.82
Arm swing (degrees) [mean]	Gait	1 minute walk	Smartwatch	home	3rd vs 4th at-home measurement	113	0.92	(0.89 0.95)	0.93
Arm swing (degrees) [std]	Gait	1 minute walk	Smartwatch	clinic	Baseline vs. 1st clinic visit	68	0.4	(0.19 0.59)	0.44
Arm swing (degrees) [std]	Gait	1 minute walk	Smartwatch	home	1st vs 2nd at-home measurement	121	0.32	(0.15 0.47)	0.33
Arm swing (degrees) [std]	Gait	1 minute walk	Smartwatch	home	2nd vs 3rd at-home measurement	117	0.41	(0.25 0.55)	0.45
Arm swing (degrees) [std]	Gait	1 minute walk	Smartwatch	home	3rd vs 4th at-home measurement	112	0.52	(0.37 0.64)	0.49
Cepstral peak prominence, zscored	Speech	Phonation	Smartphone	clinic	Baseline vs. 1st clinic visit	68	0.57	[0.38 0.71]	0.6
Cepstral peak prominence, zscored	Speech	Phonation	Smartphone	home	1st vs 2nd at-home measurement	122	0.64	[0.52 0.74]	0.62
Cepstral peak prominence, zscored	Speech	Phonation	Smartphone	home	2nd vs 3rd at-home measurement	122	0.63	[0.51 0.73]	0.65
Cepstral peak prominence, zscored	Speech	Phonation	Smartphone	home	3rd vs 4th at-home measurement	119	0.73	[0.63 0.8]	0.75
Log(pause time), zscored	Speech	Phonation	Smartphone	clinic	Baseline vs. 1st clinic visit	68	0.34	[0.12 0.54]	0.36
Log(pause time), zscored	Speech	Phonation	Smartphone	home	1st vs 2nd at-home measurement	122	0.5	[0.35 0.62]	0.48
Log(pause time), zscored	Speech	Phonation	Smartphone	home	2nd vs 3rd at-home measurement	122	0.54	[0.4 0.66]	0.54
Log(pause time), zscored	Speech	Phonation	Smartphone	home	3rd vs 4th at-home measurement	119	0.5	[0.36 0.63]	0.46
Mfcc2, zscored	Speech	Phonation	Smartphone	clinic	Baseline vs. 1st clinic visit	68	0.38	[0.16 0.57]	0.38
Mfcc2, zscored	Speech	Phonation	Smartphone	home	1st vs 2nd at-home measurement	121	0.48	[0.33 0.6]	0.47
Mfcc2, zscored	Speech	Phonation	Smartphone	home	2nd vs 3rd at-home measurement	120	0.6	[0.48 0.71]	0.59
Mfcc2, zscored	Speech	Phonation	Smartphone	home	3rd vs 4th at-home measurement	118	0.53	[0.39 0.65]	0.47
Monopitch (semitone range), zscored	Speech	Reading	Smartphone	clinic	Baseline vs. 1st clinic visit	68	0.64	[0.48 0.76]	0.64
Monopitch (semitone range), zscored	Speech	Reading	Smartphone	home	1st vs 2nd at-home measurement	122	0.67	[0.56 0.75]	0.67
Monopitch (semitone range), zscored	Speech	Reading	Smartphone	home	2nd vs 3rd at-home measurement	122	0.66	[0.55 0.75]	0.66
Monopitch (semitone range), zscored	Speech	Reading	Smartphone	home	3rd vs 4th at-home measurement	119	0.67	[0.55 0.76]	0.63
Speech composite	Speech	Phonation, reading	Smartphone	clinic	Baseline vs. 1st clinic visit	68	0.61	[0.43 0.74]	0.57
Speech composite	Speech	Phonation, reading	Smartphone	home	1st vs 2nd at-home measurement	122	0.63	[0.51 0.73]	0.57
Speech composite	Speech	Phonation, reading	Smartphone	home	2nd vs 3rd at-home measurement	122	0.63	[0.51 0.73]	0.59
Speech composite	Speech	Phonation, reading	Smartphone	home	3rd vs 4th at-home measurement	119	0.66	[0.55 0.75]	0.65
Angle (degrees) [mean]	Turn	2 minute walk	Research accel.	clinic	Baseline vs. 1st clinic visit	110	0.33	(0.15 0.48)	0.42
Angle (degrees) [mean]	Turn	Dual task	Research accel.	clinic	Baseline vs. 1st clinic visit	110	0.63	(0.5 0.73)	0.49
Angle (degrees) [std]	Turn	2 minute walk	Research accel.	clinic	Baseline vs. 1st clinic visit	110	0.06	(-0.13 0.24)	0.34
Angle (degrees) [std]	Turn	Dual task	Research accel.	clinic	Baseline vs. 1st clinic visit	110	0.44	(0.27 0.58)	0.48
Duration (s) [mean]	Turn	2 minute walk	Research accel.	clinic	Baseline vs. 1st clinic visit	110	0.7	(0.59 0.78)	0.71
Duration (s) [mean]	Turn	Dual task	Research accel.	clinic	Baseline vs. 1st clinic visit	110	0.78	(0.7 0.85)	0.76
Duration (s) [std]	Turn	2 minute walk	Research accel.	clinic	Baseline vs. 1st clinic visit	110	0.38	(0.21 0.53)	0.43
Duration (s) [std]	Turn	Dual task	Research accel.	clinic	Baseline vs. 1st clinic visit	110	0.43	(0.26 0.57)	0.4
Steps in turn (#) [mean]	Turn	2 minute walk	Research accel.	clinic	Baseline vs. 1st clinic visit	110	0.58	(0.45 0.69)	0.56
Steps in turn (#) [mean]	Turn	Dual task	Research accel.	clinic	Baseline vs. 1st clinic visit	110	0.74	(0.65 0.82)	0.74
Steps in turn (#) [std]	Turn	2 minute walk	Research accel.	clinic	Baseline vs. 1st clinic visit	110	0.12	(-0.07 0.3)	0.15
Steps in turn (#) [std]	Turn	Dual task	Research accel.	clinic	Baseline vs. 1st clinic visit	110	0.15	(-0.03 0.33)	0.16
Turn velocity (degrees/s) [mean]	Turn	2 minute walk	Research accel.	clinic	Baseline vs. 1st clinic visit	110	0.83	(0.76 0.88)	0.83
Turn velocity (degrees/s) [mean]	Turn	Dual task	Research accel.	clinic	Baseline vs. 1st clinic visit	110	0.86	(0.8 0.9)	0.85
Turn velocity (degrees/s) [std]	Turn	2 minute walk	Research accel.	clinic	Baseline vs. 1st clinic visit	110	0.36	(0.18 0.51)	0.38
Turn velocity (degrees/s) [std]	Turn	Dual task	Research accel.	clinic	Baseline vs. 1st clinic visit	110	0.35	(0.17 0.5)	0.28
Turn duration (s) [std]	Turn	1 minute walk	Smartphone	clinic	Baseline vs. 1st clinic visit	108	0.21	(0.02 0.38)	0.1
Turn duration (s) [std]	Turn	1 minute walk	Smartphone	home	1st vs 2nd at-home measurement	125	0.28	(0.11 0.43)	0.29
Turn duration (s) [std]	Turn	1 minute walk	Smartphone	home	2nd vs 3rd at-home measurement	119	0.31	(0.14 0.46)	0.36
Turn duration (s) [std]	Turn	1 minute walk	Smartphone	home	3rd vs 4th at-home measurement	116	0.5	(0.35 0.63)	0.47

Supplementary Table 2: Model-Fitted Analysis Results Utilizing Generalized Additive Model (GAM). Std. Diff., Standardized Difference; CI, Confidence Interval. MDS-UPDRS, the Movement Disorder Society-Sponsored Revision of the Unified Parkinson's Disease Rating Scale. SDMT, Symbol-Digit Modalities Test. VSWM, Visuospatial Working Memory Task. GCT, Gait Cycle Time. PD, Parkinson's Disease.

**Feature**	**Domain**	**Task**	**Device**	**Location**	**Cohort**	**n**	**Month 0 (Mean & SD)**	**Month 12 (Mean & SD)**	**Std. Diff**	**Std. Diff 95% CI**	**Pvalues**
Finemotor dominant hand	Psychomotor	Finemotor	Smartphone	clinic	Controls	48	5.1 (1.0)	6.0 (1.2)	0.7	(0.4, 1.1)	<0.001
Finemotor dominant hand	Psychomotor	Finemotor	Smartphone	clinic	PD	78	3.9 (1.2)	4.3 (1.6)	0.3	(-0.04, 0.6)	0.09
Finemotor dominant hand	Psychomotor	Finemotor	Smartphone	home	Controls	46	5.3 (0.9)	6.0 (1.2)	0.5	(0.3, 0.8)	<0.001
Finemotor dominant hand	Psychomotor	Finemotor	Smartphone	home	PD	76	3.9 (1.2)	4.3 (1.6)	0.3	(-0.2, 0.7)	0.21
Finemotor less affected hand	Psychomotor	Finemotor	Smartphone	clinic	PD	78	4.0 (1.3)	4.5 (1.5)	0.4	(0.1, 0.7)	0.005
Finemotor less affected hand	Psychomotor	Finemotor	Smartphone	home	PD	76	4.0 (1.2)	5.0 (1.5)	0.8	(0.3, 1.2)	<0.001
Finemotor more affected side	Psychomotor	Finemotor	Smartphone	clinic	PD	78	3.6 (1.1)	3.6 (1.3)	0.04	(-0.3, 0.4)	0.83
Finemotor more affected side	Psychomotor	Finemotor	Smartphone	home	PD	76	3.7 (1.1)	3.3 (1.2)	-0.3	(-0.5, 0.02)	0.07
Finemotor nondominant hand	Psychomotor	Finemotor	Smartphone	clinic	Controls	48	4.5 (1.1)	5.5 (1.2)	0.9	(0.5, 1.3)	<0.001
Finemotor nondominant hand	Psychomotor	Finemotor	Smartphone	clinic	PD	78	3.8 (1.2)	3.9 (1.3)	0.1	(-0.2, 0.4)	0.39
Finemotor nondominant hand	Psychomotor	Finemotor	Smartphone	home	Controls	46	4.7 (1.1)	5.2 (1.2)	0.4	(0.09, 0.6)	0.008
Finemotor nondominant hand	Psychomotor	Finemotor	Smartphone	home	PD	76	3.9 (1.1)	3.9 (1.3)	0.01	(-0.3, 0.3)	0.93
Fingertapping dominant hand	Psychomotor	Fingertapping	Smartphone	clinic	Controls	48	135.9 (28.8)	160.7 (36.6)	1	(0.5, 1.4)	<0.001
Fingertapping dominant hand	Psychomotor	Fingertapping	Smartphone	clinic	PD	78	107.7 (36.6)	144.3 (52.2)	1.3	(0.8, 1.8)	<0.001
Fingertapping dominant hand	Psychomotor	Fingertapping	Smartphone	home	Controls	46	145.4 (34.7)	156.3 (38.4)	0.6	(-0.006, 1.1)	0.05
Fingertapping dominant hand	Psychomotor	Fingertapping	Smartphone	home	PD	76	116.6 (41.5)	141.2 (54.1)	1	(0.5, 1.5)	<0.001
Fingertapping less affected hand	Psychomotor	Fingertapping	Smartphone	clinic	PD	78	115.1 (38.3)	142.2 (48.7)	1	(0.5, 1.4)	<0.001
Fingertapping less affected hand	Psychomotor	Fingertapping	Smartphone	home	PD	76	122.2 (43.9)	146.5 (52.9)	1.1	(0.6, 1.6)	<0.001
Fingertapping more affected hand	Psychomotor	Fingertapping	Smartphone	clinic	PD	78	102.0 (34.7)	135.8 (52.7)	1.2	(0.7, 1.8)	<0.001
Fingertapping more affected hand	Psychomotor	Fingertapping	Smartphone	home	PD	76	111.0 (40.9)	132.7 (52.2)	0.8	(0.4, 1.3)	<0.001
Fingertapping nondominant hand	Psychomotor	Fingertapping	Smartphone	clinic	Controls	48	126.9 (28.6)	151.3 (35.6)	1.1	(0.6, 1.7)	<0.001
Fingertapping nondominant hand	Psychomotor	Fingertapping	Smartphone	clinic	PD	78	109.3 (37.2)	134.4 (48.5)	0.9	(0.5, 1.4)	<0.001
Fingertapping nondominant hand	Psychomotor	Fingertapping	Smartphone	home	Controls	46	130.8 (32.2)	149.1 (36.8)	1.2	(0.5, 1.8)	<0.001
Fingertapping nondominant hand	Psychomotor	Fingertapping	Smartphone	home	PD	76	116.5 (43.6)	137.9 (51.3)	0.9	(0.4, 1.4)	<0.001
Intertap intervals (ms) dominant hand	Psychomotor	Intertap intervals	Smartphone	clinic	Controls	48	161.4 (19.3)	130.9 (37.2)	-0.4	(-0.7, -0.05)	0.02
Intertap intervals (ms) dominant hand	Psychomotor	Intertap intervals	Smartphone	clinic	PD	78	206.0 (54.9)	154.0 (86.4)	-0.5	(-0.9, -0.2)	0.005
Intertap intervals (ms) dominant hand	Psychomotor	Intertap intervals	Smartphone	home	Controls	46	140.7 (36.7)	110.9 (42.5)	-0.5	(-0.7, -0.2)	<0.001
Intertap intervals (ms) dominant hand	Psychomotor	Intertap intervals	Smartphone	home	PD	76	178.1 (63.3)	152.1 (96.0)	-0.3	(-0.6, 0.1)	0.15
Intertap intervals (ms) less affected side	Psychomotor	Intertap intervals	Smartphone	clinic	PD	78	203.1 (71.2)	147.6 (98.8)	-0.6	(-1.0, -0.2)	0.002
Intertap intervals (ms) less affected side	Psychomotor	Intertap intervals	Smartphone	home	PD	76	176.9 (69.3)	138.9 (90.8)	-0.4	(-0.7, -0.1)	0.005
Intertap intervals (ms) more affected side	Psychomotor	Intertap intervals	Smartphone	clinic	PD	76	218.0 (53.3)	160.7 (80.3)	-0.6	(-0.9, -0.2)	0.002
Intertap intervals (ms) more affected side	Psychomotor	Intertap intervals	Smartphone	home	PD	76	206.1 (65.1)	177.6 (92.5)	-0.3	(-0.6, 0.09)	0.14
Intertap intervals (ms) nondominant hand	Psychomotor	Intertap intervals	Smartphone	clinic	Controls	48	146.7 (30.8)	124.5 (54.3)	-0.3	(-0.6, 0.04)	0.08
Intertap intervals (ms) nondominant hand	Psychomotor	Intertap intervals	Smartphone	clinic	PD	77	221.6 (64.7)	157.5 (83.5)	-0.6	(-0.9, -0.3)	<0.001
Intertap intervals (ms) nondominant hand	Psychomotor	Intertap intervals	Smartphone	home	Controls	46	147.5 (40.1)	116.4 (50.5)	-0.4	(-0.7, -0.1)	0.003
Intertap intervals (ms) nondominant hand	Psychomotor	Intertap intervals	Smartphone	home	PD	76	205.4 (76.3)	163.7 (92.5)	-0.4	(-0.7, -0.1)	0.007
Percentage total correct from SDMT	Cognitive	SDMT	Smartphone	clinic	Controls	49	21.7 (3.7)	25.4 (4.0)	0.8	(0.5, 1.1)	<0.001
Percentage total correct from SDMT	Cognitive	SDMT	Smartphone	clinic	PD	80	19.7 (4.7)	21.2 (5.5)	0.3	(0, 0.6)	0.05
Percentage total correct from SDMT	Cognitive	SDMT	Smartphone	home	Controls	47	22.5 (4.0)	26.4 (4.1)	0.8	(0.4, 1.2)	<0.001
Percentage total correct from SDMT	Cognitive	SDMT	Smartphone	home	PD	78	20.5 (4.4)	22.2 (4.9)	0.3	(0.05, 0.6)	0.02
Percentage total correct from VSWM	Cognitive	VSWM	Smartphone	clinic	Controls	48	74.4 (6.9)	80.6 (8.3)	0.5	(0.2, 0.9)	<0.001
Percentage total correct from VSWM	Cognitive	VSWM	Smartphone	clinic	PD	78	74.3 (6.6)	77.9 (8.0)	0.3	(0.02, 0.6)	0.04
Percentage total correct from VSWM	Cognitive	VSWM	Smartphone	home	Controls	46	77.5 (6.7)	81.5 (8.5)	0.3	(0.09, 0.6)	0.007
Percentage total correct from VSWM	Cognitive	VSWM	Smartphone	home	PD	76	73.9 (5.6)	80.7 (6.5)	0.5	(0.3, 0.7)	<0.001
Trail making task A time (ms)	Cognitive	Trails	Smartphone	clinic	Controls	49	23912.0 (5277.0)	18461.0 (4901.0)	-0.7	(-1.1, -0.4)	<0.001
Trail making task A time (ms)	Cognitive	Trails	Smartphone	clinic	PD	80	27843.0 (5745.0)	23520.0 (6684.0)	-0.5	(-0.8, -0.2)	<0.001
Trail making task A time (ms)	Cognitive	Trails	Smartphone	home	Controls	47	22717.0 (4123.0)	18239.0 (4120.0)	-0.7	(-1.0, -0.4)	<0.001
Trail making task A time (ms)	Cognitive	Trails	Smartphone	home	PD	78	24719.0 (5109.0)	23091.0 (6599.0)	-0.2	(-0.5, 0.04)	0.09
Trail making task B minus trail making task A (ms)	Cognitive	Trails	Smartphone	clinic	Controls	49	296.0 (1526.0)	1862.0 (1526.0)	0.1	(-0.1, 0.4)	0.24
Trail making task B minus trail making task A (ms)	Cognitive	Trails	Smartphone	clinic	PD	79	550.0 (3085.0)	3826.0 (3169.0)	0.3	(0.06, 0.6)	0.02
Trail making task B minus trail making task A (ms)	Cognitive	Trails	Smartphone	home	Controls	47	538.0 (862.0)	1522.0 (861.0)	0.1	(-0.09, 0.3)	0.27
Trail making task B minus trail making task A (ms)	Cognitive	Trails	Smartphone	home	PD	78	660.0 (778.0)	1208.0 (781.0)	0.06	(-0.1, 0.2)	0.53
Trail making task B time (ms)	Cognitive	Trails	Smartphone	clinic	Controls	49	24870.0 (3838.0)	19656.0 (3913.0)	-0.6	(-0.9, -0.3)	<0.001
Trail making task B time (ms)	Cognitive	Trails	Smartphone	clinic	PD	80	28195.0 (6705.0)	27397.0 (8730.0)	-0.09	(-0.4, 0.3)	0.61
Trail making task B time (ms)	Cognitive	Trails	Smartphone	home	Controls	47	23245.0 (3789.0)	19476.0 (3791.0)	-0.6	(-0.8, -0.3)	<0.001
Trail making task B time (ms)	Cognitive	Trails	Smartphone	home	PD	78	27227.0 (5401.0)	24785.0 (6398.0)	-0.3	(-0.6, -0.06)	0.02
Arm swing (deg) average of both arms	Gait	2 minute walk	Research accel.	clinic	Controls	48	54.9 (15.2)	53.6 (17.2)	-0.2	(-0.6, 0.3)	0.46
Arm swing (deg) average of both arms	Gait	2 minute walk	Research accel.	clinic	PD	78	33.5 (16.3)	28.5 (16.6)	-0.8	(-1.1, -0.5)	<0.001
Arm swing (deg) average of both arms	Gait	Dual task	Research accel.	clinic	Controls	48	54.2 (15.2)	55.0 (18.3)	0.09	(-0.4, 0.6)	0.72
Arm swing (deg) average of both arms	Gait	Dual task	Research accel.	clinic	PD	77	32.3 (17.0)	27.5 (17.2)	-0.9	(-1.2, -0.5)	<0.001
Arm swing (deg) less affected arm	Gait	2 minute walk	Research accel.	clinic	PD	78	24.6 (16.9)	20.5 (16.4)	-0.6	(-0.9, -0.2)	<0.001
Arm swing (deg) less affected arm	Gait	Dual task	Research accel.	clinic	PD	77	21.8 (16.8)	17.7 (16.3)	-0.6	(-0.9, -0.3)	<0.001
Arm swing (deg) more affected arm	Gait	2 minute walk	Research accel.	clinic	PD	78	42.4 (20.3)	36.5 (20.9)	-0.7	(-1.0, -0.3)	<0.001
Arm swing (deg) more affected arm	Gait	Dual task	Research accel.	clinic	PD	77	42.4 (22.4)	36.9 (23.0)	-0.7	(-1.1, -0.4)	<0.001
Arm swing (deg) watch arm	Gait	1 minute walk	Smartwatch	clinic	Controls	45	47.5 (17.2)	47.3 (17.8)	-0.01	(-0.3, 0.3)	0.94

Arm swing (deg) watch arm	Gait	1 minute walk	Smartwatch	clinic	PD	66	25.9 (15.3)	19.9 (13.7)	-0.6	(-1.0, -0.2)	0.004
Arm swing (deg) watch arm	Gait	1 minute walk	Smartwatch	home	Controls	47	44.5 (13.9)	44.7 (20.1)	0.01	(-0.5, 0.5)	0.96
Arm swing (deg) watch arm	Gait	1 minute walk	Smartwatch	home	PD	71	23.0 (12.9)	20.6 (12.5)	-0.3	(-0.6, 0.03)	0.07
Arm swing velocity(deg/s) average of both arms	Gait	2 minute walk	Research accel.	clinic	Controls	48	233.7 (58.0)	227.7 (66.2)	-0.2	(-0.5, 0.2)	0.38
Arm swing velocity(deg/s) average of both arms	Gait	2 minute walk	Research accel.	clinic	PD	78	169.5 (76.5)	157.9 (77.0)	-0.4	(-0.7, -0.09)	0.01
Arm swing velocity(deg/s) average of both arms	Gait	Dual task	Research accel.	clinic	Controls	48	236.5 (59.4)	234.7 (71.0)	-0.05	(-0.5, 0.4)	0.84
Arm swing velocity(deg/s) average of both arms	Gait	Dual task	Research accel.	clinic	PD	77	168.4 (80.2)	157.9 (83.6)	-0.4	(-0.7, -0.02)	0.04
Arm swing velocity(deg/s) less affected arm	Gait	2 minute walk	Research accel.	clinic	PD	78	157.5 (85.8)	149.6 (85.5)	-0.2	(-0.6, 0.1)	0.23
Arm swing velocity(deg/s) less affected arm	Gait	Dual task	Research accel.	clinic	PD	77	151.8 (86.5)	150.4 (89.9)	-0.03	(-0.3, 0.3)	0.84
Arm swing velocity(deg/s) more affected arm	Gait	2 minute walk	Research accel.	clinic	PD	78	181.4 (86.6)	166.2 (87.6)	-0.5	(-0.8, -0.1)	0.004
Arm swing velocity(deg/s) more affected arm	Gait	Dual task	Research accel.	clinic	PD	77	185.1 (95.6)	165.3 (97.9)	-0.7	(-1.0, -0.3)	<0.001
Cadence (steps/min)	Gait	1 minute walk	Smartphone	clinic	Controls	45	113.4 (8.5)	113.6 (8.8)	0.03	(-0.3, 0.3)	0.87
Cadence (steps/min)	Gait	1 minute walk	Smartphone	clinic	PD	65	108.5 (7.9)	107.9 (7.9)	-0.08	(-0.4, 0.3)	0.67
Cadence (steps/min)	Gait	1 minute walk	Smartphone	home	Controls	47	111.3 (9.7)	110.1 (10.1)	-0.2	(-0.5, 0.2)	0.31
Cadence (steps/min)	Gait	1 minute walk	Smartphone	home	PD	71	107.1 (8.7)	106.2 (9.0)	-0.1	(-0.5, 0.2)	0.43
Cadence (steps/min) average of both legs	Gait	2 minute walk	Research accel.	clinic	Controls	48	113.6 (9.3)	113.1 (8.9)	-0.1	(-0.5, 0.3)	0.55
Cadence (steps/min) average of both legs	Gait	2 minute walk	Research accel.	clinic	PD	78	108.8 (7.2)	107.4 (7.7)	-0.4	(-0.7, 0.02)	0.06
Cadence (steps/min) average of both legs	Gait	Dual task	Research accel.	clinic	Controls	48	109.0 (9.7)	109.3 (9.5)	0.07	(-0.3, 0.5)	0.74
Cadence (steps/min) average of both legs	Gait	Dual task	Research accel.	clinic	PD	77	104.2 (8.1)	104.3 (8.3)	0.02	(-0.3, 0.3)	0.88
Circumduction (cm) average of both legs	Gait	2 minute walk	Research accel.	clinic	Controls	48	3.3 (1.1)	3.4 (1.1)	0.09	(-0.3, 0.4)	0.61
Circumduction (cm) average of both legs	Gait	2 minute walk	Research accel.	clinic	PD	78	3.4 (1.2)	3.4 (1.2)	-0.1	(-0.4, 0.2)	0.47
Circumduction (cm) average of both legs	Gait	Dual task	Research accel.	clinic	Controls	48	3.4 (1.2)	3.4 (1.3)	0.006	(-0.3, 0.3)	0.97
Circumduction (cm) average of both legs	Gait	Dual task	Research accel.	clinic	PD	77	3.4 (1.2)	3.3 (1.2)	-0.09	(-0.4, 0.2)	0.51
Coronal range of motion (deg)	Gait	2 minute walk	Research accel.	clinic	Controls	48	6.7 (1.8)	6.5 (2.0)	-0.1	(-0.4, 0.2)	0.47
Coronal range of motion (deg)	Gait	2 minute walk	Research accel.	clinic	PD	78	6.1 (1.7)	6.2 (1.9)	0.02	(-0.3, 0.3)	0.89
Double support (%GCT) average of both legs	Gait	2 minute walk	Research accel.	clinic	Controls	48	21.4 (3.1)	21.1 (3.6)	-0.1	(-0.5, 0.3)	0.5
Double support (%GCT) average of both legs	Gait	2 minute walk	Research accel.	clinic	PD	78	21.5 (2.8)	22.0 (3.0)	0.3	(-0.1, 0.6)	0.2
Double support (%GCT) average of both legs	Gait	Dual task	Research accel.	clinic	Controls	48	22.6 (3.3)	22.4 (3.8)	-0.1	(-0.6, 0.3)	0.61
Double support (%GCT) average of both legs	Gait	Dual task	Research accel.	clinic	PD	77	22.9 (3.3)	23.0 (3.5)	0.01	(-0.3, 0.4)	0.94
Double support (s)	Gait	1 minute walk	Smartphone	clinic	Controls	44	0.3 (0.02)	0.3 (0.02)	-0.2	(-0.6, 0.1)	0.2
Double support (s)	Gait	1 minute walk	Smartphone	clinic	PD	65	0.3 (0.02)	0.3 (0.02)	-0.002	(-0.5, 0.5)	0.99
Double support (s)	Gait	1 minute walk	Smartphone	home	Controls	47	0.3 (0.03)	0.3 (0.03)	0.3	(-0.2, 0.7)	0.21
Double support (s)	Gait	1 minute walk	Smartphone	home	PD	71	0.3 (0.02)	0.3 (0.03)	0.1	(-0.2, 0.4)	0.4
Double support (s) asymetry	Gait	1 minute walk	Smartphone	clinic	Controls	43	1.1 (0.3)	0.9 (0.2)	-0.2	(-0.5, 0.1)	0.26
Double support (s) asymetry	Gait	1 minute walk	Smartphone	clinic	PD	64	1.0 (0.4)	1.1 (0.5)	0.09	(-0.2, 0.4)	0.59
Double support (s) asymetry	Gait	1 minute walk	Smartphone	home	Controls	47	1.5 (0.5)	1.2 (0.5)	-0.3	(-0.5, -0.007)	0.04
Double support (s) asymetry	Gait	1 minute walk	Smartphone	home	PD	69	1.8 (0.6)	1.8 (0.8)	-0.05	(-0.4, 0.3)	0.77
Elevation at midswing (cm) average of both arms	Gait	2 minute walk	Research accel.	clinic	Controls	48	1.4 (0.5)	1.4 (0.7)	-0.007	(-0.4, 0.4)	0.97
Elevation at midswing (cm) average of both arms	Gait	2 minute walk	Research accel.	clinic	PD	78	1.5 (0.5)	1.6 (0.6)	0.2	(-0.1, 0.5)	0.21
Elevation at midswing (cm) average of both arms	Gait	Dual task	Research accel.	clinic	Controls	48	1.3 (0.6)	1.2 (0.7)	-0.05	(-0.5, 0.4)	0.83
Elevation at midswing (cm) average of both arms	Gait	Dual task	Research accel.	clinic	PD	77	1.4 (0.6)	1.5 (0.6)	0.3	(-0.01, 0.6)	0.06
Foot strike angle (deg) average of both legs	Gait	2 minute walk	Research accel.	clinic	Controls	47	25.6 (3.7)	25.1 (3.7)	-0.1	(-0.5, 0.2)	0.36
Foot strike angle (deg) average of both legs	Gait	2 minute walk	Research accel.	clinic	PD	78	22.8 (4.5)	21.0 (4.7)	-0.8	(-1.1, -0.4)	<0.001
Gait cycle duration (s)	Gait	2 minute walk	Research accel.	clinic	Controls	48	1.1 (0.1)	1.1 (0.09)	0	(-0.5, 0.5)	1
Gait cycle duration (s)	Gait	2 minute walk	Research accel.	clinic	PD	78	1.1 (0.07)	1.1 (0.08)	0.3	(-0.01, 0.7)	0.06
Gait cycle duration (s)	Gait	Dual task	Research accel.	clinic	Controls	48	1.1 (0.1)	1.1 (0.1)	-0.1	(-0.5, 0.3)	0.52
Gait cycle duration (s)	Gait	Dual task	Research accel.	clinic	PD	77	1.2 (0.09)	1.2 (0.09)	-0.03	(-0.3, 0.2)	0.83
Gait speed (m/s)	Gait	1 minute walk	Smartphone	clinic	Controls	44	1.1 (0.2)	1.1 (0.2)	0.1	(-0.2, 0.4)	0.5
Gait speed (m/s)	Gait	1 minute walk	Smartphone	clinic	PD	67	1.0 (0.1)	0.9 (0.2)	-0.6	(-1.0, -0.1)	0.01
Gait speed (m/s)	Gait	1 minute walk	Smartphone	home	Controls	46	1.0 (0.1)	1.0 (0.1)	-0.1	(-0.5, 0.2)	0.41
Gait speed (m/s)	Gait	1 minute walk	Smartphone	home	PD	71	0.9 (0.1)	0.9 (0.2)	-0.1	(-0.4, 0.2)	0.5
Gait speed (m/s) average of both legs	Gait	2 minute walk	Research accel.	clinic	Controls	48	1.2 (0.2)	1.2 (0.1)	-0.2	(-0.6, 0.3)	0.48
Gait speed (m/s) average of both legs	Gait	2 minute walk	Research accel.	clinic	PD	78	1.1 (0.1)	1.0 (0.2)	-0.7	(-1.1, -0.3)	<0.001
Gait speed (m/s) average of both legs	Gait	Dual task	Research accel.	clinic	Controls	48	1.1 (0.2)	1.1 (0.2)	-0.04	(-0.5, 0.4)	0.86
Gait speed (m/s) average of both legs	Gait	Dual task	Research accel.	clinic	PD	77	1.0 (0.2)	1.0 (0.2)	-0.3	(-0.7, 0.06)	0.1
Initial double support (s)	Gait	1 minute walk	Smartphone	clinic	Controls	45	0.1 (0.01)	0.1 (0.01)	-0.2	(-0.5, 0.1)	0.27
Initial double support (s)	Gait	1 minute walk	Smartphone	clinic	PD	65	0.1 (0.01)	0.1 (0.01)	-0.08	(-0.6, 0.4)	0.73
Initial double support (s)	Gait	1 minute walk	Smartphone	home	Controls	47	0.1 (0.01)	0.1 (0.01)	0.2	(-0.3, 0.6)	0.44
Initial double support (s)	Gait	1 minute walk	Smartphone	home	PD	71	0.1 (0.01)	0.1 (0.01)	0.2	(-0.1, 0.5)	0.22
Initial double support (s) asymetry	Gait	1 minute walk	Smartphone	clinic	Controls	42	5.8 (2.2)	5.5 (2.3)	-0.06	(-0.4, 0.3)	0.7
Initial double support (s) asymetry	Gait	1 minute walk	Smartphone	clinic	PD	66	6.2 (3.3)	7.9 (4.9)	0.4	(-0.08, 0.9)	0.1
Initial double support (s) asymetry	Gait	1 minute walk	Smartphone	home	Controls	47	6.6 (2.0)	6.5 (3.0)	-0.02	(-0.3, 0.3)	0.92
Initial double support (s) asymetry	Gait	1 minute walk	Smartphone	home	PD	70	8.8 (3.5)	8.3 (4.1)	-0.09	(-0.4, 0.2)	0.55
Rater evaluation of gait	Gait	item 3.10	Clinical rating	clinic	Controls	48	0.04 (0.2)	0.07 (0.3)	0.4	(-0.2, 1.0)	0.25
Rater evaluation of gait	Gait	item 3.10	Clinical rating	clinic	PD	78	0.7 (0.4)	0.7 (0.4)	0.06	(-0.2, 0.3)	0.65
Self-reported walking and balance	Gait	item 2.12	Self reported evalution	clinic	Controls	48	0.02 (0.02)	0.04 (0.06)	0.08	(-0.2, 0.3)	0.56
Self-reported walking and balance	Gait	item 2.12	Self reported evalution	clinic	PD	78	0.3 (0.3)	0.5 (0.5)	0.4	(0.04, 0.8)	0.03
Single limb support (%GCT) average of both legs	Gait	2 minute walk	Research accel.	clinic	Controls	48	39.3 (1.6)	39.4 (1.8)	0.1	(-0.2, 0.5)	0.48
Single limb support (%GCT) average of both legs	Gait	2 minute walk	Research accel.	clinic	PD	78	39.2 (1.4)	39.0 (1.5)	-0.3	(-0.7, 0.1)	0.18
Single limb support (%GCT) average of both legs	Gait	Dual task	Research accel.	clinic	Controls	48	24.8 (4.3)	24.0 (4.2)	-0.3	(-0.6, 0.08)	0.13
Single limb support (%GCT) average of both legs	Gait	Dual task	Research accel.	clinic	PD	77	21.1 (5.2)	19.7 (5.4)	-0.6	(-1.0, -0.2)	0.003
Single limb support (%GCT) average of both legs	Gait	Dual task	Research accel.	clinic	Controls	48	38.7 (1.7)	38.8 (1.9)	0.1	(-0.3, 0.6)	0.59

Single limb support (%GCT) average of both legs	Gait	Dual task	Research accel.	clinic	PD	77	38.5 (1.6)	38.5 (1.7)	-0.03	(-0.4, 0.3)	0.88
Single limb support (s)	Gait	1 minute walk	Smartphone	clinic	Controls	44	0.4 (0.03)	0.4 (0.03)	-0.09	(-0.5, 0.3)	0.64
Single limb support (s)	Gait	1 minute walk	Smartphone	clinic	PD	65	0.4 (0.03)	0.4 (0.03)	-0.01	(-0.4, 0.4)	0.95
Single limb support (s)	Gait	1 minute walk	Smartphone	clinic	Controls	43	4.0 (2.4)	5.2 (2.8)	0.4	(-0.05, 0.8)	0.08
Single limb support (s)	Gait	1 minute walk	Smartphone	clinic	PD	67	4.5 (1.6)	5.7 (2.2)	0.3	(-0.04, 0.7)	0.08
Single limb support (s)	Gait	1 minute walk	Smartphone	clinic	Controls	43	0.6 (0.001)	0.6 (0.001)	-0.4	(-0.7, -0.07)	0.02
Single limb support (s)	Gait	1 minute walk	Smartphone	home	Controls	47	0.4 (0.04)	0.4 (0.04)	0.2	(-0.2, 0.6)	0.26
Single limb support (s)	Gait	1 minute walk	Smartphone	home	PD	71	0.4 (0.03)	0.4 (0.04)	-0.03	(-0.4, 0.3)	0.86
Single limb support (s)	Gait	1 minute walk	Smartphone	home	Controls	47	6.0 (2.8)	6.3 (3.9)	0.05	(-0.3, 0.4)	0.77
Single limb support (s)	Gait	1 minute walk	Smartphone	home	PD	71	6.8 (2.4)	6.9 (2.9)	0.02	(-0.2, 0.3)	0.87
Stance (%GCT) average of both legs	Gait	2 minute walk	Research accel.	clinic	Controls	48	60.7 (1.5)	60.5 (1.8)	-0.1	(-0.5, 0.3)	0.53
Stance (%GCT) average of both legs	Gait	2 minute walk	Research accel.	clinic	PD	78	60.7 (1.4)	60.9 (1.5)	0.2	(-0.1, 0.6)	0.22
Stance (%GCT) average of both legs	Gait	Dual task	Research accel.	clinic	Controls	48	61.3 (1.7)	61.2 (1.9)	-0.1	(-0.6, 0.4)	0.64
Stance (%GCT) average of both legs	Gait	Dual task	Research accel.	clinic	PD	77	61.5 (1.6)	61.5 (1.7)	0.002	(-0.4, 0.4)	0.99
Stance (s)	Gait	1 minute walk	Smartphone	clinic	Controls	45	0.7 (0.05)	0.7 (0.05)	-0.2	(-0.6, 0.2)	0.29
Stance (s)	Gait	1 minute walk	Smartphone	clinic	PD	65	0.7 (0.05)	0.7 (0.05)	-0.01	(-0.5, 0.4)	0.95
Stance (s)	Gait	1 minute walk	Smartphone	home	Controls	47	0.7 (0.06)	0.7 (0.06)	0.2	(-0.2, 0.6)	0.35
Stance (s)	Gait	1 minute walk	Smartphone	home	PD	71	0.7 (0.06)	0.7 (0.06)	0.2	(-0.1, 0.5)	0.27
Stance (s) asymmetry	Gait	1 minute walk	Smartphone	clinic	Controls	43	2.7 (1.4)	3.2 (1.6)	0.3	(-0.1, 0.6)	0.16
Stance (s) asymmetry	Gait	1 minute walk	Smartphone	clinic	PD	66	2.6 (0.9)	3.7 (1.3)	0.5	(0.1, 0.8)	0.01
Stance (s) asymmetry	Gait	1 minute walk	Smartphone	home	Controls	47	3.8 (1.7)	3.7 (2.3)	-0.02	(-0.4, 0.3)	0.91
Stance (s) asymmetry	Gait	1 minute walk	Smartphone	home	PD	71	4.5 (1.4)	4.8 (1.5)	0.07	(-0.2, 0.3)	0.53
Step duration (s)	Gait	1 minute walk	Smartphone	clinic	Controls	44	0.5 (0.04)	0.5 (0.04)	-0.2	(-0.5, 0.2)	0.32
Step duration (s)	Gait	1 minute walk	Smartphone	clinic	PD	65	0.6 (0.04)	0.6 (0.04)	0.009	(-0.4, 0.4)	0.97
Step duration (s)	Gait	1 minute walk	Smartphone	home	Controls	47	0.5 (0.05)	0.6 (0.05)	0.2	(-0.2, 0.6)	0.33
Step duration (s)	Gait	1 minute walk	Smartphone	home	PD	71	0.6 (0.04)	0.6 (0.05)	0.09	(-0.2, 0.4)	0.55
Step duration (s) asymmetry	Gait	1 minute walk	Smartphone	clinic	Controls	43	3.8 (1.7)	4.4 (2.2)	0.2	(-0.2, 0.6)	0.29
Step duration (s) asymmetry	Gait	1 minute walk	Smartphone	clinic	PD	65	4.0 (1.5)	5.7 (2.9)	0.6	(0.09, 1.0)	0.02
Step duration (s) asymmetry	Gait	1 minute walk	Smartphone	home	Controls	47	5.3 (2.6)	5.1 (2.9)	-0.05	(-0.4, 0.2)	0.74
Step duration (s) asymmetry	Gait	1 minute walk	Smartphone	home	PD	71	6.9 (2.3)	6.9 (2.6)	-0.001	(-0.2, 0.2)	1
Step duration (s) average of both legs	Gait	2 minute walk	Research accel.	clinic	Controls	48	0.5 (0.05)	0.5 (0.04)	0.01	(-0.5, 0.5)	0.95
Step duration (s) average of both legs	Gait	2 minute walk	Research accel.	clinic	PD	78	0.6 (0.04)	0.6 (0.04)	0.3	(-0.009, 0.7)	0.05
Step duration (s) average of both legs	Gait	Dual task	Research accel.	clinic	Controls	48	0.6 (0.05)	0.6 (0.05)	-0.1	(-0.5, 0.3)	0.49
Step duration (s) average of both legs	Gait	Dual task	Research accel.	clinic	PD	77	0.6 (0.05)	0.6 (0.05)	-0.03	(-0.3, 0.2)	0.85
Step length (m)	Gait	1 minute walk	Smartphone	clinic	Controls	44	0.6 (0.08)	0.6 (0.08)	0.2	(-0.08, 0.5)	0.15
Step length (m)	Gait	1 minute walk	Smartphone	clinic	PD	67	0.6 (0.06)	0.5 (0.09)	-0.6	(-1.1, -0.2)	0.008
Step length (m)	Gait	1 minute walk	Smartphone	home	Controls	46	0.5 (0.06)	0.5 (0.06)	-0.05	(-0.4, 0.3)	0.74
Step length (m)	Gait	1 minute walk	Smartphone	home	PD	71	0.5 (0.06)	0.5 (0.07)	0.04	(-0.3, 0.3)	0.77
Step length (m) asymmetry	Gait	1 minute walk	Smartphone	clinic	Controls	43	5.9 (1.8)	5.7 (2.6)	-0.04	(-0.4, 0.3)	0.81
Step length (m) asymmetry	Gait	1 minute walk	Smartphone	clinic	PD	66	6.9 (4.1)	9.1 (5.4)	0.4	(-0.07, 0.9)	0.09
Step length (m) asymmetry	Gait	1 minute walk	Smartphone	home	Controls	46	7.9 (3.2)	7.6 (3.7)	-0.05	(-0.4, 0.3)	0.73
Step length (m) asymmetry	Gait	1 minute walk	Smartphone	home	PD	69	9.1 (3.0)	10.2 (4.5)	0.2	(-0.1, 0.5)	0.21
Stride duration	Gait	1 minute walk	Smartphone	clinic	Controls	45	1.1 (0.08)	1.1 (0.08)	-0.1	(-0.4, 0.2)	0.52
Stride duration	Gait	1 minute walk	Smartphone	clinic	PD	65	1.1 (0.08)	1.1 (0.08)	0.003	(-0.4, 0.5)	0.99
Stride duration	Gait	1 minute walk	Smartphone	home	Controls	47	1.1 (0.09)	1.1 (0.1)	0.2	(-0.2, 0.5)	0.31
Stride duration	Gait	1 minute walk	Smartphone	home	PD	71	1.1 (0.09)	1.1 (0.09)	0.08	(-0.2, 0.4)	0.62
Stride duration asymmetry	Gait	1 minute walk	Smartphone	clinic	Controls	43	0.9 (0.3)	0.8 (0.4)	-0.1	(-0.5, 0.2)	0.46
Stride duration asymmetry	Gait	1 minute walk	Smartphone	clinic	PD	66	0.7 (0.2)	1.2 (0.9)	0.8	(0.2, 1.4)	0.008
Stride duration asymmetry	Gait	1 minute walk	Smartphone	home	Controls	47	1.0 (0.4)	0.8 (0.4)	-0.2	(-0.5, 0.1)	0.24
Stride duration asymmetry	Gait	1 minute walk	Smartphone	home	PD	70	1.3 (0.4)	1.2 (0.5)	-0.07	(-0.3, 0.2)	0.61
Stride length (m)	Gait	1 minute walk	Smartphone	clinic	Controls	44	1.1 (0.1)	1.2 (0.1)	0.2	(-0.09, 0.5)	0.16
Stride length (m)	Gait	1 minute walk	Smartphone	clinic	PD	67	1.1 (0.1)	1.0 (0.2)	-0.7	(-1.1, -0.2)	0.007
Stride length (m)	Gait	1 minute walk	Smartphone	home	Controls	46	1.1 (0.1)	1.0 (0.1)	-0.06	(-0.4, 0.3)	0.72
Stride length (m)	Gait	1 minute walk	Smartphone	home	PD	71	1.0 (0.1)	1.0 (0.1)	0.05	(-0.2, 0.3)	0.73
Stride length (m) asymmetry	Gait	1 minute walk	Smartphone	clinic	Controls	42	1.2 (0.2)	0.9 (0.2)	-0.2	(-0.5, 0.03)	0.08
Stride length (m) asymmetry	Gait	1 minute walk	Smartphone	clinic	PD	65	1.1 (0.4)	1.3 (0.4)	0.1	(-0.1, 0.4)	0.3
Stride length (m) asymmetry	Gait	1 minute walk	Smartphone	home	Controls	46	1.8 (0.7)	1.5 (0.8)	-0.2	(-0.5, 0.1)	0.2
Stride length (m) asymmetry	Gait	1 minute walk	Smartphone	home	PD	70	2.3 (0.6)	2.0 (1.0)	-0.2	(-0.5, 0.2)	0.27
Stride length (m) average of both legs	Gait	2 minute walk	Research accel.	clinic	Controls	48	1.2 (0.1)	1.2 (0.1)	-0.09	(-0.5, 0.3)	0.65
Stride length (m) average of both legs	Gait	2 minute walk	Research accel.	clinic	PD	78	1.2 (0.1)	1.2 (0.1)	-0.8	(-1.2, -0.4)	<0.001
Stride length (m) average of both legs	Gait	Dual task	Research accel.	clinic	Controls	48	1.2 (0.1)	1.2 (0.1)	-0.04	(-0.5, 0.4)	0.85
Stride length (m) average of both legs	Gait	Dual task	Research accel.	clinic	PD	77	1.1 (0.1)	1.1 (0.1)	-0.5	(-0.8, -0.1)	0.008
Swing (%GCT) average of both legs	Gait	2 minute walk	Research accel.	clinic	Controls	48	39.3 (1.5)	39.5 (1.8)	0.1	(-0.3, 0.5)	0.53
Swing (%GCT) average of both legs	Gait	2 minute walk	Research accel.	clinic	PD	78	39.3 (1.4)	39.1 (1.5)	-0.2	(-0.6, 0.1)	0.22
Swing (%GCT) average of both legs	Gait	Dual task	Research accel.	clinic	Controls	48	38.7 (1.7)	38.8 (1.9)	0.1	(-0.4, 0.6)	0.64
Swing (%GCT) average of both legs	Gait	Dual task	Research accel.	clinic	PD	77	38.5 (1.6)	38.5 (1.7)	-0.002	(-0.4, 0.4)	0.99
Swing (s)	Gait	1 minute walk	Smartphone	clinic	Controls	44	0.4 (0.03)	0.4 (0.03)	-0.05	(-0.4, 0.3)	0.76
Swing (s)	Gait	1 minute walk	Smartphone	clinic	PD	65	0.4 (0.03)	0.4 (0.03)	0.01	(-0.4, 0.5)	0.96
Swing (s)	Gait	1 minute walk	Smartphone	home	Controls	47	0.4 (0.03)	0.4 (0.04)	0.3	(-0.01, 0.7)	0.06
Swing (s)	Gait	1 minute walk	Smartphone	home	PD	71	0.4 (0.03)	0.4 (0.04)	-0.02	(-0.4, 0.3)	0.93
Swing (s) asymmetry	Gait	1 minute walk	Smartphone	clinic	Controls	43	3.7 (1.7)	5.1 (2.8)	0.4	(-0.02, 0.8)	0.06
Swing (s) asymmetry	Gait	1 minute walk	Smartphone	clinic	PD	66	4.0 (1.4)	5.8 (2.5)	0.5	(0.09, 0.9)	0.02

Swing (s) asymmetry	Gait	1 minute walk	Smartphone	home	Controls	47	5.8 (2.6)	5.9 (3.4)	0.02	(-0.3, 0.3)	0.9
Swing (s) asymmetry	Gait	1 minute walk	Smartphone	home	PD	71	7.1 (2.5)	7.5 (3.0)	0.09	(-0.2, 0.3)	0.47
Terminal double support (%GCT) average of both legs	Gait	2 minute walk	Research accel.	clinic	Controls	48	10.7 (1.5)	10.6 (1.8)	-0.1	(-0.5, 0.3)	0.54
Terminal double support (%GCT) average of both legs	Gait	2 minute walk	Research accel.	clinic	PD	78	10.8 (1.4)	11.0 (1.5)	0.2	(-0.1, 0.6)	0.21
Terminal double support (%GCT) average of both legs	Gait	Dual task	Research accel.	clinic	Controls	48	11.3 (1.7)	11.2 (1.9)	-0.1	(-0.6, 0.3)	0.6
Terminal double support (%GCT) average of both legs	Gait	Dual task	Research accel.	clinic	PD	77	11.4 (1.7)	11.5 (1.8)	0.2	(-0.2, 0.5)	0.42
Terminal double support (s)	Gait	1 minute walk	Smartphone	clinic	Controls	44	0.1 (0.01)	0.1 (0.01)	-0.2	(-0.6, 0.1)	0.22
Terminal double support (s)	Gait	1 minute walk	Smartphone	clinic	PD	65	0.1 (0.01)	0.1 (0.01)	0.04	(-0.5, 0.6)	0.89
Terminal double support (s)	Gait	1 minute walk	Smartphone	home	Controls	47	0.1 (0.01)	0.1 (0.01)	0.2	(-0.2, 0.6)	0.26
Terminal double support (s)	Gait	1 minute walk	Smartphone	home	PD	71	0.1 (0.01)	0.1 (0.01)	0.09	(-0.2, 0.4)	0.55
Terminal double support (s) asymmetry	Gait	1 minute walk	Smartphone	clinic	Controls	43	5.2 (2.4)	5.2 (2.4)	-0.02	(-0.3, 0.3)	0.91
Terminal double support (s) asymmetry	Gait	1 minute walk	Smartphone	clinic	PD	65	5.7 (3.4)	8.3 (5.8)	0.8	(0.2, 1.4)	0.01
Terminal double support (s) asymmetry	Gait	1 minute walk	Smartphone	home	Controls	47	6.1 (1.7)	6.5 (2.6)	0.07	(-0.2, 0.4)	0.64
Terminal double support (s) asymmetry	Gait	1 minute walk	Smartphone	home	PD	68	8.2 (3.4)	8.3 (3.8)	0.03	(-0.2, 0.3)	0.84
Toe off angle (deg) average of both legs	Gait	2 minute walk	Research accel.	clinic	Controls	48	36.7 (2.7)	37.1 (3.1)	0.2	(-0.1, 0.5)	0.29
Toe off angle (deg) average of both legs	Gait	2 minute walk	Research accel.	clinic	PD	78	35.9 (3.6)	34.7 (4.1)	-0.6	(-1.0, -0.2)	0.004
Toe off angle (deg) average of both legs	Gait	Dual task	Research accel.	clinic	Controls	48	35.7 (2.9)	36.1 (3.3)	0.2	(-0.2, 0.5)	0.28
Toe off angle (deg) average of both legs	Gait	Dual task	Research accel.	clinic	PD	77	34.9 (3.8)	33.9 (4.1)	-0.5	(-0.9, -0.1)	0.01
Toe out angle (deg) average of both legs	Gait	2 minute walk	Research accel.	clinic	Controls	48	7.2 (5.0)	8.8 (5.4)	0.3	(-0.007, 0.6)	0.05
Toe out angle (deg) average of both legs	Gait	2 minute walk	Research accel.	clinic	PD	78	7.7 (5.9)	8.1 (6.1)	0.07	(-0.2, 0.4)	0.62
Toe out angle (deg) average of both legs	Gait	Dual task	Research accel.	clinic	Controls	48	7.3 (5.1)	9.0 (5.7)	0.3	(-0.001, 0.6)	0.05
Toe out angle (deg) average of both legs	Gait	Dual task	Research accel.	clinic	PD	77	8.1 (6.1)	8.6 (6.4)	0.09	(-0.2, 0.4)	0.52
Rater evaluation of speech	Speech	item 3.01	MDS - UPDRS	clinic	PD	78	0.5 (0.4)	0.8 (0.5)	0.7	(0.3, 1.0)	<0.001
Rater evaluation of speech	Speech	item 3.01	MDS - UPDRS	clinic	Controls	48	0 (0)	0 (0)			
Self-reported problem with speech	Speech	item 2.01	MDS - UPDRS	clinic	PD	78	0.4 (0.6)	0.6 (0.6)	0.3	(-0.005, 0.7)	0.05
Self-reported problem with speech	Speech	item 2.01	MDS - UPDRS	clinic	Controls	48	0.02 (0.07)	-0.004 (0.07)	-0.1	(-0.3, 0.1)	0.41
Speech composite	Speech	Phonation, reading	Smartphone	clinic	Controls	46	-0.1 (1.4)	-0.3 (1.4)	-0.08	(-0.3, 0.1)	0.49
Speech composite	Speech	Phonation, reading	Smartphone	clinic	PD	70	1.5 (2.0)	1.6 (2.2)	0.03	(-0.3, 0.3)	0.85
Speech composite	Speech	Phonation, reading	Smartphone	home	Controls	46	0.09 (1.3)	0.2 (1.4)	0.06	(-0.2, 0.3)	0.67
Speech composite	Speech	Phonation, reading	Smartphone	home	PD	75	1.2 (1.9)	1.7 (2.0)	0.3	(0.02, 0.5)	0.03
Log(pause time), zscored	Speech	Reading	Smartphone	clinic	Controls	46	-0.1 (0.6)	0.05 (0.7)	0.1	(-0.2, 0.4)	0.37
Log(pause time), zscored	Speech	Reading	Smartphone	clinic	PD	70	0.2 (0.8)	0.2 (0.8)	0.04	(-0.2, 0.3)	0.72
Log(pause time), zscored	Speech	Reading	Smartphone	home	Controls	46	-0.4 (0.5)	0.003 (0.8)	0.3	(-0.009, 0.6)	0.06
Log(pause time), zscored	Speech	Reading	Smartphone	home	PD	75	0.03 (0.7)	0.4 (0.7)	0.4	(0.2, 0.5)	<0.001
Monopitch (semitone range), zscored	Speech	Reading	Smartphone	clinic	Controls	46	-0.06 (0.8)	0.1 (0.8)	0.2	(-0.08, 0.4)	0.2
Monopitch (semitone range), zscored	Speech	Reading	Smartphone	clinic	PD	70	-0.7 (0.9)	-0.6 (0.9)	0.1	(-0.1, 0.4)	0.38
Monopitch (semitone range), zscored	Speech	Reading	Smartphone	home	Controls	46	-0.1 (0.7)	0.08 (0.8)	0.2	(-0.01, 0.5)	0.06
Monopitch (semitone range), zscored	Speech	Reading	Smartphone	home	PD	75	-0.7 (0.9)	-0.6 (1.0)	0.09	(-0.2, 0.4)	0.51
MFCC2, zscored	Speech	Phonation	Smartphone	clinic	Controls	46	0.2 (0.8)	0.1 (0.8)	-0.005	(-0.3, 0.3)	0.97
MFCC2, zscored	Speech	Phonation	Smartphone	clinic	PD	70	-0.3 (0.6)	-0.3 (0.9)	-0.07	(-0.3, 0.2)	0.62
MFCC2, zscored	Speech	Phonation	Smartphone	home	Controls	46	-0.1 (0.7)	-0.09 (0.8)	0.02	(-0.3, 0.3)	0.91
MFCC2, zscored	Speech	Phonation	Smartphone	home	PD	75	-0.09 (0.9)	-0.2 (1.1)	-0.1	(-0.4, 0.2)	0.36
Cepstral peak prominence, zscored	Speech	Phonation	Smartphone	clinic	Controls	46	-0.09 (0.7)	0.1 (0.8)	0.2	(-0.08, 0.5)	0.16
Cepstral peak prominence, zscored	Speech	Phonation	Smartphone	clinic	PD	70	-0.4 (1.0)	-0.4 (1.3)	-0.01	(-0.4, 0.3)	0.94
Cepstral peak prominence, zscored	Speech	Phonation	Smartphone	home	Controls	46	-0.1 (0.8)	-0.07 (0.9)	0.04	(-0.3, 0.4)	0.82
Cepstral peak prominence, zscored	Speech	Phonation	Smartphone	home	PD	75	-0.3 (0.9)	-0.5 (1.1)	-0.1	(-0.4, 0.1)	0.28
Turn duration (s)	Turns	1 minute walk	Smartphone	home	Controls	47	2.2 (0.3)	2.2 (0.5)	0.04	(-0.3, 0.4)	0.81
Turn duration (s)	Turns	1 minute walk	Smartphone	home	PD	77	2.3 (0.3)	2.4 (0.4)	0.09	(-0.2, 0.3)	0.48
Turn velocity (deg/s)	Turns	2 minute walk	Research accel.	clinic	Controls	48	201.3 (37.2)	206.5 (46.7)	0.2	(-0.2, 0.6)	0.29
Turn velocity (deg/s)	Turns	2 minute walk	Research accel.	clinic	PD	78	164.0 (26.4)	153.6 (31.4)	-0.6	(-1.0, -0.2)	0.004
Turn velocity (deg/s)	Turns	Dual task	Research accel.	clinic	Controls	48	193.0 (34.2)	197.7 (42.9)	0.2	(-0.2, 0.6)	0.36
Turn velocity (deg/s)	Turns	Dual task	Research accel.	clinic	PD	78	154.0 (27.8)	144.8 (29.7)	-0.5	(-0.9, -0.1)	0.008
Turns angle (deg)	Turns	2 minute walk	Research accel.	clinic	Controls	48	182.5 (3.1)	182.5 (3.1)	0.001	(-0.3, 0.3)	0.93
Turns angle (deg)	Turns	2 minute walk	Research accel.	clinic	PD	78	178.3 (4.6)	175.6 (6.2)	-0.5	(-0.8, -0.1)	0.005
Turns angle (deg)	Turns	Dual task	Research accel.	clinic	Controls	48	180.2 (3.8)	181.2 (4.5)	0.1	(-0.2, 0.5)	0.43
Turns angle (deg)	Turns	Dual task	Research accel.	clinic	PD	78	175.6 (8.4)	175.0 (8.4)	-0.07	(-0.3, 0.2)	0.54
Tremor (%)	Tremor	passive	Smartwatch	home	Controls	25	0.7 (0.6)	0.7 (0.5)	-0.1	(-0.4, 0.2)	0.57
Tremor (%)	Tremor	passive	Smartwatch	home	PD	57	19.3 (18.0)	25.6 (21.4)	0.7	(0.3, 1.0)	<0.001
Self-reported problems with tremor	Tremor	item 2.10	Self reported evalution	clinic	Controls	48	0 (0)	0 (0)			
Self-reported problems with tremor	Tremor	item 2.10	Self reported evalution	clinic	PD	78	1.2 (0.5)	1.4 (0.6)	0.4	(0.07, 0.7)	0.02
Rater evaluation of constancy of tremor	Tremor	item 3.18	Clinical rating	clinic	Controls	48	-0.001 (0)	0.01 (0.06)	0.2	(-0.2, 0.6)	0.26
Rater evaluation of constancy of tremor	Tremor	item 3.18	Clinical rating	clinic	PD	78	1.6 (1.4)	2.0 (1.5)	0.5	(0.2, 0.8)	0.001
MDS - UPDRS part I total			MDS - UPDRS	clinic	Controls	48	2.8 (2.5)	2.9 (2.8)	0.05	(-0.3, 0.4)	0.75
MDS - UPDRS part I total			MDS - UPDRS	clinic	PD	78	5.4 (2.7)	6.2 (3.4)	0.3	(-0.06, 0.7)	0.1
MDS - UPDRS part II total			MDS - UPDRS	clinic	Controls	48	0.4 (0.8)	0.4 (0.8)	-0.04	(-0.4, 0.3)	0.79
MDS - UPDRS part II total			MDS - UPDRS	clinic	PD	78	5.4 (3.5)	7.7 (4.3)	1.2	(0.7, 1.6)	<0.001
MDS - UPDRS part III total			MDS - UPDRS	clinic	Controls	48	2.7 (2.6)	2.9 (3.2)	0.1	(-0.3, 0.5)	0.57
MDS - UPDRS part III total			MDS - UPDRS	clinic	PD	78	23.6 (9.6)	29.7 (11.2)	1.2	(0.7, 1.6)	<0.001
MDS - UPDRS part I + II + III total			MDS - UPDRS	clinic	Controls	48	6.0 (4.1)	6.3 (4.6)	0.1	(-0.3, 0.5)	0.62
MDS - UPDRS part I + II + III total			MDS - UPDRS	clinic	PD	78	34.6 (11.5)	43.9 (14.4)	1.3	(0.8, 1.8)	<0.001

Supplementary Table 3: Empirical Values Analysis Results. Std. Diff., Standardized Difference; CI, Confidence Interval. MDS-UPDRS, the Movement Disorder Society-Sponsored Revision of the Unified Parkinson's Disease Rating Scale. SDMT, Symbol-Digit Modalities Test. VSWM, Visuospatial Working Memory Task. GCT, Gait Cycle Time. PD, Parkinson's Disease.

**Feature**	**Domain**	**Task**	**Device**	**Location**	**Cohort**	**n**	**Month 0 (Mean & SD)**	**Month 12 (Mean & SD)**	**Std. Diff**	**Std. Diff 95% CI**
Steps	Activity	Passive	Smartwatch	home	PD	10	3052.6 (1306.3)	2331.2 (2010.2)	-0.5	(-2.0, 0.2)
Steps per hour	Activity	Passive	Smartwatch	home	PD	10	197.9 (82.4)	159.6 (142.6)	-0.4	(-2.5, 0.2)
Finemotor dominant hand	Cognitive	Finemotor	Smartphone	clinic	Controls	42	5.0 (1.4)	6.0 (1.5)	0.8	(-1.1, -0.4)
Finemotor dominant hand	Cognitive	Finemotor	Smartphone	clinic	PD	44	4.0 (1.4)	4.5 (1.8)	0.3	(0.5, 1.2)
Finemotor dominant hand	Cognitive	Finemotor	Smartphone	home	Controls	34	5.4 (1.2)	6.1 (1.3)	0.5	(-0.8, -0.3)
Finemotor dominant hand	Cognitive	Finemotor	Smartphone	home	PD	33	3.9 (1.8)	4.5 (2.0)	0.4	(0.1, 0.9)
Finemotor less affected side	Cognitive	Finemotor	Smartphone	clinic	PD	44	4.0 (1.5)	4.8 (1.8)	0.6	(0.5, 1.2)
Finemotor less affected side	Cognitive	Finemotor	Smartphone	home	PD	33	4.2 (1.6)	4.8 (1.7)	0.5	(0.3, 1.0)
Finemotor more affected side	Cognitive	Finemotor	Smartphone	clinic	PD	44	3.7 (1.5)	3.8 (1.6)	0.02	(0.6, 1.1)
Finemotor more affected side	Cognitive	Finemotor	Smartphone	home	PD	33	3.7 (1.6)	3.5 (1.7)	-0.1	(0.1, 0.8)
Finemotor nondominant hand	Cognitive	Finemotor	Smartphone	clinic	Controls	43	4.4 (1.5)	5.6 (1.5)	0.9	(-1.3, -0.5)
Finemotor nondominant hand	Cognitive	Finemotor	Smartphone	clinic	PD	44	3.7 (1.5)	4.1 (1.7)	0.3	(0.6, 1.3)
Finemotor nondominant hand	Cognitive	Finemotor	Smartphone	home	Controls	34	4.5 (1.4)	5.2 (1.3)	0.6	(-0.6, -0.09)
Finemotor nondominant hand	Cognitive	Finemotor	Smartphone	home	PD	33	3.9 (1.4)	3.9 (1.6)	0	(0.3, 0.9)
Fingertapping dominant hand	Cognitive	Fingertapping	Smartphone	clinic	Controls	43	134.1 (42.6)	158.1 (35.2)	0.5	(-1.4, -0.5)
Fingertapping dominant hand	Cognitive	Fingertapping	Smartphone	clinic	PD	47	109.7 (41.5)	153.0 (50.8)	0.9	(0.2, 0.9)
Fingertapping dominant hand	Cognitive	Fingertapping	Smartphone	home	Controls	34	142.9 (40.0)	160.3 (35.1)	0.6	(-1.1, 0.006)
Fingertapping dominant hand	Cognitive	Fingertapping	Smartphone	home	PD	34	129.4 (45.9)	155.2 (45.8)	0.5	(0.3, 0.9)
Fingertapping less affected side	Cognitive	Fingertapping	Smartphone	clinic	PD	47	115.9 (43.7)	147.7 (51.6)	0.7	(0.2, 0.9)
Fingertapping less affected side	Cognitive	Fingertapping	Smartphone	home	PD	34	131.3 (48.6)	155.9 (44.9)	0.6	(0.7, 1.4)
Fingertapping more affected side	Cognitive	Fingertapping	Smartphone	clinic	PD	47	104.1 (38.1)	147.5 (48.9)	0.9	(0.2, 0.9)
Fingertapping more affected side	Cognitive	Fingertapping	Smartphone	home	PD	34	120.4 (50.3)	145.8 (48.7)	0.5	(0.3, 1.0)
Fingertapping nondominant hand	Cognitive	Fingertapping	Smartphone	clinic	Controls	43	127.0 (39.7)	149.6 (34.9)	0.5	(-1.7, -0.6)
Fingertapping nondominant hand	Cognitive	Fingertapping	Smartphone	clinic	PD	47	110.3 (41.4)	142.2 (49.1)	0.7	(0.2, 0.8)
Fingertapping nondominant hand	Cognitive	Fingertapping	Smartphone	home	Controls	34	128.7 (36.6)	154.0 (35.6)	0.9	(-1.8, -0.5)
Fingertapping nondominant hand	Cognitive	Fingertapping	Smartphone	home	PD	34	122.3 (53.1)	146.4 (47.9)	0.6	(0.7, 1.4)
Intertap intervals (ms) more affected side	Cognitive	Intertap intervals	Smartphone	clinic	PD	45	220.4 (94.8)	146.9 (99.7)	-0.5	(-0.5, 0.1)
Intertap intervals (ms) more affected side	Cognitive	Intertap intervals	Smartphone	home	PD	32	191.3 (95.6)	167.4 (93.4)	-0.2	(-0.8, -0.2)
Intertap intervals (ms) less affected side	Cognitive	Intertap intervals	Smartphone	clinic	PD	45	201.6 (91.5)	136.7 (96.6)	-0.5	(-0.6, 0.03)
Intertap intervals (ms) less affected side	Cognitive	Intertap intervals	Smartphone	home	PD	32	167.2 (95.3)	127.0 (98.7)	-0.4	(-0.7, 0.03)
Intertap intervals (ms) dominant hand	Cognitive	Intertap intervals	Smartphone	clinic	Controls	46	160.7 (82.9)	135.5 (71.0)	-0.2	(0.05, 0.7)
Intertap intervals (ms) dominant hand	Cognitive	Intertap intervals	Smartphone	clinic	PD	46	209.3 (98.3)	140.2 (98.7)	-0.5	(-0.6, 0.07)
Intertap intervals (ms) dominant hand	Cognitive	Intertap intervals	Smartphone	home	Controls	34	149.3 (72.0)	109.7 (58.9)	-0.5	(0.2, 0.7)
Intertap intervals (ms) dominant hand	Cognitive	Intertap intervals	Smartphone	home	PD	31	165.5 (83.6)	143.2 (90.5)	-0.2	(-0.9, -0.2)
Intertap intervals (ms) nondominant hand	Cognitive	Intertap intervals	Smartphone	clinic	Controls	44	149.5 (79.8)	130.2 (80.1)	-0.2	(-0.04, 0.6)
Intertap intervals (ms) nondominant hand	Cognitive	Intertap intervals	Smartphone	clinic	PD	47	227.5 (103.5)	152.1 (112.4)	-0.6	(-0.5, 0.08)
Intertap intervals (ms) nondominant hand	Cognitive	Intertap intervals	Smartphone	home	Controls	34	149.7 (62.1)	126.4 (74.8)	-0.3	(0.1, 0.7)
Intertap intervals (ms) nondominant hand	Cognitive	Intertap intervals	Smartphone	home	PD	33	192.6 (105.9)	156.9 (109.3)	-0.3	(-0.6, 0.03)
Percentage total correct from SDMT	Cognitive	DSST	Smartphone	clinic	Controls	43	21.5 (5.7)	25.7 (4.6)	0.7	(-1.1, -0.5)
Percentage total correct from SDMT	Cognitive	DSST	Smartphone	clinic	PD	48	19.5 (6.2)	21.2 (6.3)	0.3	(0.5, 1.0)
Percentage total correct from SDMT	Cognitive	DSST	Smartphone	home	Controls	34	21.8 (6.5)	27.0 (4.0)	0.7	(-1.2, -0.4)
Percentage total correct from SDMT	Cognitive	DSST	Smartphone	home	PD	36	21.5 (5.6)	22.9 (7.0)	0.3	(0.4, 1.1)
Percentage total correct from VSWM	Cognitive	VSWM	Smartphone	home	Controls	34	77.5 (13.2)	81.2 (13.0)	0.3	(-0.6, -0.09)
Percentage total correct from VSWM	Cognitive	VSWM	Smartphone	home	PD	35	74.7 (9.8)	80.2 (11.3)	0.4	(-0.07, 0.7)
Percentage total correct from VSWM	Cognitive	VSWM	Smartphone	clinic	Controls	43	74.1 (12.4)	81.7 (11.4)	0.6	(-0.9, -0.2)
Percentage total correct from VSWM	Cognitive	VSWM	Smartphone	clinic	PD	46	71.8 (10.0)	77.2 (10.2)	0.4	(0.3, 1.0)
Trail making task A time (ms)	Cognitive	Trails	Smartphone	clinic	Controls	40	23362.5 (10285.5)	18376.5 (6343.4)	-0.5	(0.4, 1.1)
Trail making task A time (ms)	Cognitive	Trails	Smartphone	clinic	PD	45	27672.4 (10021.3)	22592.3 (8320.6)	-0.5	(-0.8, -0.2)
Trail making task A time (ms)	Cognitive	Trails	Smartphone	home	Controls	32	21954.3 (8452.3)	17111.4 (5323.2)	-0.5	(0.4, 1.0)
Trail making task A time (ms)	Cognitive	Trails	Smartphone	home	PD	31	23426.8 (6735.1)	23110.8 (9428.3)	-0.04	(-0.9, -0.2)
Trail making task B time (ms)	Cognitive	Trails	Smartphone	clinic	Controls	39	24649.6 (7996.4)	18731.9 (4839.2)	-0.7	(0.3, 0.9)
Trail making task B time (ms)	Cognitive	Trails	Smartphone	clinic	PD	38	28894.2 (10020.4)	25006.5 (10811.4)	-0.3	(-1.1, -0.4)
Trail making task B time (ms)	Cognitive	Trails	Smartphone	home	Controls	34	22237.9 (7366.7)	18077.5 (4205.9)	-0.6	(0.3, 0.8)
Trail making task B time (ms)	Cognitive	Trails	Smartphone	home	PD	33	25400.5 (8707.2)	21997.9 (7854.5)	-0.5	(-0.9, -0.3)
Trail making task B minus trail making task A (ms)	Cognitive	Trails	Smartphone	clinic	Controls	32	77.1 (8758.5)	727.0 (4544.5)	0.07	(-0.3, 0.09)
Trail making task B minus trail making task A (ms)	Cognitive	Trails	Smartphone	clinic	PD	31	951.5 (8268.4)	-1255.0 (5812.5)	-0.2	(-0.3, 0.4)
Trail making task B minus trail making task A (ms)	Cognitive	Trails	Smartphone	home	Controls	32	77.1 (8758.5)	727.0 (4544.5)	0.07	(-0.3, 0.09)
Trail making task B minus trail making task A (ms)	Cognitive	Trails	Smartphone	home	PD	31	951.5 (8268.4)	-1255.0 (5812.5)	-0.2	(-0.3, 0.4)
Rater evaluation of gait	Gait	item 3.10	Clinical rating	clinic	Controls	45	0.04 (0.2)	0.07 (0.3)	0.1	(-1.0, 0.2)
Rater evaluation of gait	Gait	item 3.10	Clinical rating	clinic	PD	47	0.7 (0.5)	0.7 (0.5)	0.05	(0.1, 0.3)
Self-reported walking and balance	Gait	item 2.12	Clinical rating	clinic	Controls	46	0 (0)	0.04 (0.3)	0.1	(-0.3, 0.2)
Self-reported walking and balance	Gait	item 2.12	Clinical rating	clinic	PD	47	0.3 (0.5)	0.5 (0.6)	0.3	(0.1, 0.3)
Arm swing (degrees) [mean] average of both arms	Gait	2 minute walk	Research accel.	clinic	Controls	43	56.9 (15.4)	53.3 (16.8)	-0.4	(-0.3, 0.6)
Arm swing (degrees) [mean] average of both arms	Gait	Dual task	Research accel.	clinic	Controls	44	55.1 (17.5)	55.0 (18.5)	-0.01	(-0.6, 0.4)
Arm swing (degrees) [mean] average of both arms	Gait	2 minute walk	Research accel.	clinic	PD	46	36.8 (18.1)	31.3 (18.3)	-0.7	(-0.7, -0.06)
Arm swing (degrees) [mean] average of both arms	Gait	Dual task	Research accel.	clinic	PD	45	34.9 (17.9)	30.1 (17.7)	-0.8	(-0.4, 0.3)

Arm swing (degrees) [mean] less affected side	Gait	Dual task	Research accel.	clinic	Controls	44	56.3 (18.9)	56.7 (20.6)	0.03	(-0.6, 0.5)
Arm swing (degrees) [mean] less affected side	Gait	2 minute walk	Research accel.	clinic	PD	46	26.9 (18.4)	21.5 (16.0)	-0.5	(-0.7, -0.006)
Arm swing (degrees) [mean] less affected side	Gait	Dual task	Research accel.	clinic	PD	45	23.4 (16.2)	18.6 (13.7)	-0.5	(-0.4, 0.3)
Arm swing (degrees) [mean] more affected side	Gait	Dual task	Research accel.	clinic	Controls	44	54.0 (21.7)	53.3 (20.6)	-0.07	(-0.5, 0.3)
Arm swing (degrees) [mean] more affected side	Gait	2 minute walk	Research accel.	clinic	PD	46	46.6 (23.9)	41.1 (25.0)	-0.5	(-0.7, -0.03)
Arm swing (degrees) [mean] more affected side	Gait	Dual task	Research accel.	clinic	PD	45	46.4 (26.3)	41.5 (26.6)	-0.5	(-0.4, 0.2)
Arm swing velocity (degrees) (s) [mean] average of both arms	Gait	2 minute walk	Research accel.	clinic	Controls	43	241.2 (64.6)	226.6 (72.3)	-0.3	(-0.2, 0.5)
Arm swing velocity (degrees) (s) [mean] average of both arms	Gait	Dual task	Research accel.	clinic	Controls	44	241.4 (72.4)	234.5 (73.0)	-0.1	(-0.4, 0.5)
Arm swing velocity (degrees) (s) [mean] average of both arms	Gait	2 minute walk	Research accel.	clinic	PD	46	183.7 (88.5)	173.5 (92.5)	-0.3	(-0.7, -0.07)
Arm swing velocity (degrees) (s) [mean] average of both arms	Gait	Dual task	Research accel.	clinic	PD	45	185.6 (93.2)	175.3 (102.9)	-0.3	(-0.5, 0.1)
Arm swing velocity (degrees) (s) [mean] less affected side	Gait	2 minute walk	Research accel.	clinic	PD	46	169.3 (98.0)	160.8 (97.3)	-0.2	(-0.6, 0)
Arm swing velocity (degrees) (s) [mean] more affected side	Gait	2 minute walk	Research accel.	clinic	PD	46	198.2 (103.2)	186.1 (106.7)	-0.3	(-0.7, -0.07)
Arm swing velocity (degrees) (s) [mean] more affected side	Gait	Dual task	Research accel.	clinic	PD	45	203.8 (113.5)	185.7 (118.3)	-0.4	(-0.5, 0.08)
Arm swing velocity (degrees) (s) [mean] less affected side	Gait	Dual task	Research accel.	clinic	PD	45	167.4 (103.4)	164.9 (115.1)	-0.04	(-0.5, 0.2)
Cadence (steps/min) [mean] average of both legs	Gait	2 minute walk	Research accel.	clinic	Controls	43	114.6 (8.6)	114.1 (8.6)	-0.1	(-0.3, 0.5)
Cadence (steps/min) [mean] average of both legs	Gait	Dual task	Research accel.	clinic	Controls	44	109.5 (10.0)	110.1 (9.0)	0.1	(-0.5, 0.3)
Cadence (steps/min) [mean] average of both legs	Gait	2 minute walk	Research accel.	clinic	PD	46	109.9 (7.4)	108.4 (9.0)	-0.3	(-0.4, 0.2)
Cadence (steps/min) [mean] average of both legs	Gait	Dual task	Research accel.	clinic	PD	45	105.5 (9.0)	105.3 (9.7)	-0.04	(-0.2, 0.4)
Circumduction cm [mean] average of both legs	Gait	2 minute walk	Research accel.	clinic	Controls	43	3.2 (1.4)	3.4 (1.2)	0.2	(-0.4, 0.3)
Circumduction cm [mean] average of both legs	Gait	Dual task	Research accel.	clinic	Controls	44	3.3 (1.4)	3.4 (1.4)	0.08	(-0.3, 0.3)
Circumduction cm [mean] average of both legs	Gait	2 minute walk	Research accel.	clinic	PD	46	3.6 (1.2)	3.5 (1.3)	-0.2	(-0.1, 0.4)
Circumduction cm [mean] average of both legs	Gait	Dual task	Research accel.	clinic	PD	45	3.6 (1.2)	3.5 (1.4)	-0.2	(-0.3, 0.3)
Double support (%GCT) [mean] average of both legs	Gait	2 minute walk	Research accel.	clinic	Controls	43	21.2 (3.4)	21.2 (3.9)	0.02	(-0.3, 0.5)
Double support (%GCT) [mean] average of both legs	Gait	Dual task	Research accel.	clinic	Controls	44	22.4 (3.7)	22.4 (4.1)	0.008	(-0.3, 0.6)
Double support (%GCT) [mean] average of both legs	Gait	2 minute walk	Research accel.	clinic	PD	46	21.1 (2.8)	21.5 (2.9)	0.2	(-0.3, 0.3)
Double support (%GCT) [mean] average of both legs	Gait	Dual task	Research accel.	clinic	PD	45	22.7 (4.2)	22.5 (3.4)	-0.05	(-0.3, 0.3)
Elevation at midswing cm [mean] average of both legs	Gait	2 minute walk	Research accel.	clinic	Controls	42	1.5 (0.6)	1.4 (0.7)	-0.1	(-0.4, 0.4)
Elevation at midswing cm [mean] average of both legs	Gait	2 minute walk	Research accel.	clinic	PD	46	1.6 (0.6)	1.7 (0.6)	0.1	(-0.5, 0.2)
Elevation at midswing cm [mean] average of both legs	Gait	Dual task	Research accel.	clinic	Controls	43	1.3 (0.6)	1.2 (0.7)	-0.1	(-0.4, 0.5)
Elevation at midswing cm [mean] average of both legs	Gait	Dual task	Research accel.	clinic	PD	45	1.5 (0.6)	1.6 (0.6)	0.2	(-0.4, 0.2)
Foot strike angle (degrees) [mean] average of both legs	Gait	2 minute walk	Research accel.	clinic	Controls	43	25.7 (5.2)	25.2 (4.2)	-0.2	(-0.2, 0.5)
Foot strike angle (degrees) [mean] average of both legs	Gait	2 minute walk	Research accel.	clinic	PD	46	23.0 (4.3)	21.4 (5.1)	-0.6	(-0.5, 0.1)
Foot.strike.angle (degrees) [mean] average of both legs	Gait	Dual task	Research accel.	clinic	Controls	43	24.6 (5.5)	24.0 (4.6)	-0.2	(-0.08, 0.6)
Foot.strike.angle (degrees) [mean] average of both legs	Gait	Dual task	Research accel.	clinic	PD	44	21.5 (5.4)	19.9 (5.9)	-0.5	(-0.6, 0.1)
Gait cycle duration (s) [mean] average of both legs	Gait	2 minute walk	Research accel.	clinic	Controls	43	1.1 (0.08)	1.1 (0.08)	0.09	(-0.5, 0.5)
Gait cycle duration (s) [mean] average of both legs	Gait	2 minute walk	Research accel.	clinic	PD	46	1.1 (0.07)	1.1 (0.09)	0.3	(-0.2, 0.4)
Gait cycle duration (s) [mean] average of both legs	Gait	Dual task	Research accel.	clinic	Controls	44	1.1 (0.1)	1.1 (0.1)	-0.1	(-0.3, 0.5)
Gait cycle duration (s) [mean] average of both legs	Gait	Dual task	Research accel.	clinic	PD	45	1.1 (0.1)	1.1 (0.1)	0.04	(-0.4, 0.2)
Lumbar coronal range of motion (degrees) [mean]	Gait	2 minute walk	Research accel.	clinic	Controls	43	6.6 (1.9)	6.4 (2.5)	-0.08	(-0.2, 0.4)
Lumbar coronal range of motion (degrees) [mean]	Gait	Dual task	Research accel.	clinic	Controls	44	6.2 (1.9)	6.1 (2.3)	-0.04	(-0.2, 0.4)
Lumbar coronal range of motion (degrees) [mean]	Gait	2 minute walk	Research accel.	clinic	PD	46	6.2 (2.0)	6.4 (2.3)	0.1	(-0.4, 0.2)
Lumbar coronal range of motion (degrees) [mean]	Gait	Dual task	Research accel.	clinic	PD	45	6.0 (2.0)	6.2 (2.3)	0.2	(-0.3, 0.3)
Lumbar sagittal range of motion (degrees) [mean]	Gait	2 minute walk	Research accel.	clinic	Controls	43	6.4 (2.7)	6.1 (2.9)	-0.1	(-0.5, 0.1)
Lumbar sagittal range of motion (degrees) [mean]	Gait	Dual task	Research accel.	clinic	Controls	44	6.3 (3.3)	5.8 (2.6)	-0.2	(-0.4, 0.2)
Lumbar sagittal range of motion (degrees) [mean]	Gait	2 minute walk	Research accel.	clinic	PD	46	5.6 (2.3)	5.5 (2.0)	-0.02	(-0.5, 0.2)
Lumbar sagittal range of motion (degrees) [mean]	Gait	Dual task	Research accel.	clinic	PD	45	5.4 (2.2)	5.3 (2.0)	-0.06	(-0.5, 0.1)
Lumbar transverse range of motion (degrees) [mean]	Gait	2 minute walk	Research accel.	clinic	Controls	43	8.4 (2.7)	8.4 (3.0)	-0.002	(-0.4, 0.3)
Lumbar transverse range of motion (degrees) [mean]	Gait	Dual task	Research accel.	clinic	Controls	44	8.7 (2.9)	8.8 (3.0)	0.08	(-0.4, 0.3)
Lumbar transverse range of motion (degrees) [mean]	Gait	2 minute walk	Research accel.	clinic	PD	46	8.1 (3.0)	8.4 (3.1)	0.1	(-0.3, 0.3)
Lumbar transverse range of motion (degrees) [mean]	Gait	Dual task	Research accel.	clinic	PD	45	7.9 (2.6)	8.3 (3.2)	0.2	(-0.2, 0.4)
Singlelimb support (%GCT) [mean] average of both legs	Gait	2 minute walk	Research accel.	clinic	Controls	43	39.4 (1.8)	39.4 (2.0)	-0.01	(-0.5, 0.2)
Singlelimb support (%GCT) [mean] average of both legs	Gait	Dual task	Research accel.	clinic	Controls	44	38.8 (1.8)	38.8 (2.0)	-0.001	(-0.6, 0.3)
Singlelimb support (%GCT) [mean] average of both legs	Gait	2 minute walk	Research accel.	clinic	PD	46	39.5 (1.4)	39.2 (1.5)	-0.2	(-0.3, 0.3)
Singlelimb support (%GCT) [mean] average of both legs	Gait	Dual task	Research accel.	clinic	PD	45	38.7 (2.1)	38.7 (1.7)	0.04	(-0.3, 0.3)
Gait speed (m/s) [mean] average of both legs	Gait	2 minute walk	Research accel.	clinic	Controls	43	1.2 (0.2)	1.2 (0.2)	-0.2	(-0.3, 0.6)
Gait speed (m/s) [mean] average of both legs	Gait	Dual task	Research accel.	clinic	Controls	44	1.1 (0.2)	1.1 (0.2)	-0.02	(-0.4, 0.5)
Gait speed (m/s) [mean] average of both legs	Gait	2 minute walk	Research accel.	clinic	PD	46	1.1 (0.1)	1.1 (0.2)	-0.5	(-0.5, 0.09)
Gait speed (m/s) [mean] average of both legs	Gait	Dual task	Research accel.	clinic	PD	45	1.0 (0.2)	1.0 (0.2)	-0.2	(-0.4, 0.3)
Stance (%GCT) [mean] average of both legs	Gait	2 minute walk	Research accel.	clinic	Controls	43	60.6 (1.7)	60.6 (2.0)	0.02	(-0.3, 0.5)
Stance (%GCT) [mean] average of both legs	Gait	Dual task	Research accel.	clinic	Controls	44	61.2 (1.8)	61.2 (2.0)	0.01	(-0.4, 0.6)
Stance (%GCT) [mean] average of both legs	Gait	2 minute walk	Research accel.	clinic	PD	46	60.5 (1.4)	60.7 (1.5)	0.2	(-0.3, 0.3)
Stance (%GCT) [mean] average of both legs	Gait	Dual task	Research accel.	clinic	PD	45	61.4 (2.1)	61.3 (1.7)	-0.06	(-0.3, 0.3)
Step duration (s) [mean] average of both legs	Gait	2 minute walk	Research accel.	clinic	Controls	43	0.5 (0.04)	0.5 (0.04)	0.1	(-0.5, 0.5)
Step duration (s) [mean] average of both legs	Gait	Dual task	Research accel.	clinic	Controls	44	0.6 (0.05)	0.5 (0.05)	-0.1	(-0.3, 0.5)
Step duration (s) [mean] average of both legs	Gait	2 minute walk	Research accel.	clinic	PD	46	0.5 (0.04)	0.6 (0.04)	0.3	(-0.2, 0.4)
Step duration (s) [mean] average of both legs	Gait	Dual task	Research accel.	clinic	PD	45	0.6 (0.05)	0.6 (0.05)	0.04	(-0.4, 0.2)
Stride length (m) [mean] average of both legs	Gait	2 minute walk	Research accel.	clinic	Controls	43	1.3 (0.2)	1.2 (0.1)	-0.3	(-0.3, 0.5)
Stride length (m) [mean] average of both legs	Gait	Dual task	Research accel.	clinic	Controls	44	1.2 (0.2)	1.2 (0.1)	-0.08	(-0.4, 0.5)
Stride length (m) [mean] average of both legs	Gait	2 minute walk	Research accel.	clinic	PD	46	1.2 (0.1)	1.2 (0.2)	-0.5	(-0.6, 0.06)
Stride length (m) [mean] average of both legs	Gait	Dual task	Research accel.	clinic	PD	45	1.2 (0.2)	1.1 (0.2)	-0.3	(-0.4, 0.2)
Swing (%GCT) [mean] average of both legs	Gait	2 minute walk	Research accel.	clinic	Controls	43	39.4 (1.7)	39.4 (2.0)	-0.02	(-0.5, 0.3)
Swing (%GCT) [mean] average of both legs	Gait	Dual task	Research accel.	clinic	Controls	44	38.8 (1.8)	38.8 (2.0)	-0.01	(-0.6, 0.4)

Swing (%GCT) [mean] average of both legs	Gait	2 minute walk	Research accel.	clinic	PD	46	39.5 (1.4)	39.3 (1.5)	-0.2	(-0.3, 0.3)
Swing (%GCT) [mean] average of both legs	Gait	Dual task	Research accel.	clinic	PD	45	38.6 (2.1)	38.7 (1.7)	0.06	(-0.3, 0.3)
Terminal double support (%GCT) [mean] average of both legs	Gait	2 minute walk	Research accel.	clinic	Controls	43	10.6 (1.7)	10.6 (2.0)	0.02	(-0.3, 0.5)
Terminal double support (%GCT) [mean] average of both legs	Gait	Dual task	Research accel.	clinic	Controls	44	11.2 (1.8)	11.2 (2.0)	-0.002	(-0.3, 0.6)
Terminal double support (%GCT) [mean] average of both legs	Gait	2 minute walk	Research accel.	clinic	PD	46	10.6 (1.4)	10.8 (1.5)	0.2	(-0.3, 0.3)
Terminal double support (%GCT) [mean] average of both legs	Gait	Dual task	Research accel.	clinic	PD	44	11.1 (1.7)	11.3 (1.7)	0.1	(-0.3, 0.3)
Toe off angle (degrees) [mean] average of both legs	Gait	2 minute walk	Research accel.	clinic	Controls	43	36.9 (3.4)	37.1 (3.4)	0.09	(-0.5, 0.1)
Toe off angle (degrees) [mean] average of both legs	Gait	Dual task	Research accel.	clinic	Controls	44	35.8 (3.5)	36.0 (3.8)	0.06	(-0.5, 0.2)
Toe off angle (degrees) [mean] average of both legs	Gait	2 minute walk	Research accel.	clinic	PD	46	36.8 (3.8)	36.0 (4.0)	-0.3	(-0.2, 0.4)
Toe off angle (degrees) [mean] average of both legs	Gait	Dual task	Research accel.	clinic	PD	45	35.7 (4.2)	35.1 (4.1)	-0.2	(-0.3, 0.4)
Toe out angle (degrees) [mean] average of both legs	Gait	2 minute walk	Research accel.	clinic	Controls	43	6.6 (6.6)	9.0 (6.5)	0.4	(-0.6, 0.007)
Toe out angle (degrees) [mean] average of both legs	Gait	Dual task	Research accel.	clinic	Controls	44	6.5 (6.7)	9.1 (6.3)	0.5	(-0.6, 0.001)
Toe out angle (degrees) [mean] average of both legs	Gait	2 minute walk	Research accel.	clinic	PD	46	6.6 (7.0)	7.0 (7.6)	0.06	(0.2, 0.8)
Toe out angle (degrees) [mean] average of both legs	Gait	Dual task	Research accel.	clinic	PD	45	6.5 (7.2)	7.3 (7.8)	0.1	(0.2, 0.8)
Trunk coronal range of motion (degrees) [mean]	Gait	2 minute walk	Research accel.	clinic	Controls	42	6.2 (2.4)	6.0 (2.1)	-0.09	(-0.4, 0.3)
Trunk coronal range of motion (degrees) [mean]	Gait	Dual task	Research accel.	clinic	Controls	43	6.7 (2.5)	6.5 (2.2)	-0.2	(-0.3, 0.4)
Trunk coronal range of motion (degrees) [mean]	Gait	2 minute walk	Research accel.	clinic	PD	46	5.1 (2.3)	4.8 (2.5)	-0.2	(-0.4, 0.2)
Trunk coronal range of motion (degrees) [mean]	Gait	Dual task	Research accel.	clinic	PD	45	5.1 (2.3)	4.9 (2.6)	-0.2	(-0.6, 0.1)
Trunk sagittal range of motion (degrees) [mean]	Gait	2 minute walk	Research accel.	clinic	Controls	42	4.8 (1.5)	4.9 (1.2)	0.1	(-0.3, 0.4)
Trunk sagittal range of motion (degrees) [mean]	Gait	Dual task	Research accel.	clinic	Controls	43	4.9 (1.5)	5.0 (1.2)	0.03	(-0.3, 0.4)
Trunk sagittal range of motion (degrees) [mean]	Gait	2 minute walk	Research accel.	clinic	PD	46	4.4 (1.0)	4.3 (1.0)	-0.09	(-0.2, 0.5)
Trunk sagittal range of motion (degrees) [mean]	Gait	Dual task	Research accel.	clinic	PD	45	4.4 (0.9)	4.3 (1.1)	-0.1	(-0.2, 0.4)
Trunk transverse range of motion (degrees) [mean]	Gait	2 minute walk	Research accel.	clinic	Controls	42	8.6 (2.5)	9.6 (4.8)	0.2	(-0.5, 0.1)
Trunk transverse range of motion (degrees) [mean]	Gait	Dual task	Research accel.	clinic	Controls	43	9.5 (3.3)	9.7 (3.7)	0.07	(-0.3, 0.2)
Trunk transverse range of motion (degrees) [mean]	Gait	2 minute walk	Research accel.	clinic	PD	46	7.8 (2.4)	7.6 (2.3)	-0.2	(0.03, 0.4)
Trunk transverse range of motion (degrees) [mean]	Gait	Dual task	Research accel.	clinic	PD	45	7.9 (2.4)	7.7 (2.6)	-0.2	(-0.3, 0.3)
Cadence (steps/min) [mean]	Gait	1 minute walk	Smartphone	clinic	Controls	34	113.4 (9.4)	113.5 (8.6)	0.02	(-0.3, 0.3)
Cadence (steps/min) [mean]	Gait	1 minute walk	Smartphone	clinic	PD	27	108.2 (10.5)	109.2 (10.2)	0.2	(-0.3, 0.4)
Cadence (steps/min) [mean]	Gait	1 minute walk	Smartphone	home	Controls	19	110.2 (14.2)	108.7 (12.9)	-0.2	(-0.2, 0.5)
Cadence (steps/min) [mean]	Gait	1 minute walk	Smartphone	home	PD	19	106.8 (11.4)	106.3 (11.8)	-0.1	(-1.0, 0.3)
Double support (s) [mean]	Gait	1 minute walk	Smartphone	clinic	Controls	34	0.3 (0.02)	0.3 (0.02)	-0.09	(-0.1, 0.6)
Double support (s) [mean]	Gait	1 minute walk	Smartphone	clinic	PD	27	0.3 (0.03)	0.3 (0.03)	-0.2	(-0.5, 0.2)
Double support (s) [mean]	Gait	1 minute walk	Smartphone	home	Controls	18	0.3 (0.03)	0.3 (0.02)	0.07	(-0.7, 0.2)
Double support (s) [mean]	Gait	1 minute walk	Smartphone	home	PD	19	0.3 (0.03)	0.3 (0.03)	-0.03	(-0.3, 0.8)
Double support (s) asymmetry	Gait	1 minute walk	Smartphone	clinic	Controls	29	1.0 (0.6)	0.9 (0.6)	-0.09	(-0.1, 0.5)
Double support (s) asymmetry	Gait	1 minute walk	Smartphone	clinic	PD	25	0.8 (0.5)	1.0 (0.7)	0.3	(-0.4, 0.3)
Double support (s) asymmetry	Gait	1 minute walk	Smartphone	home	Controls	16	1.2 (0.9)	1.0 (0.6)	-0.2	(0.007, 0.5)
Double support (s) asymmetry	Gait	1 minute walk	Smartphone	home	PD	17	1.5 (0.9)	1.5 (1.3)	-0.02	(-0.8, 0.3)
Double support (%GCT) [mean]	Gait	1 minute walk	Smartphone	clinic	Controls	34	0.3 (0.004)	0.3 (0.004)	-0.08	(-0.1, 0.6)
Double support (%GCT) [mean]	Gait	1 minute walk	Smartphone	clinic	PD	27	0.3 (0.005)	0.3 (0.004)	-0.2	(-0.5, 0.3)
Double support (%GCT) [mean]	Gait	1 minute walk	Smartphone	home	Controls	19	0.3 (0.004)	0.3 (0.004)	-0.3	(-0.1, 0.4)
Double support (%GCT) [mean]	Gait	1 minute walk	Smartphone	home	PD	17	0.3 (0.004)	0.3 (0.005)	-0.1	(-0.7, 0.2)
Double support (%GCT) asymmetry	Gait	1 minute walk	Smartphone	clinic	Controls	30	0.9 (0.5)	0.9 (0.4)	-0.1	(-0.2, 0.5)
Double support (%GCT) asymmetry	Gait	1 minute walk	Smartphone	clinic	PD	26	0.9 (0.8)	1.0 (0.5)	0.07	(-0.5, 0.3)
Double support (%GCT) asymmetry	Gait	1 minute walk	Smartphone	home	Controls	17	1.2 (0.8)	1.1 (0.5)	-0.1	(-0.1, 0.5)
Double support (%GCT) asymmetry	Gait	1 minute walk	Smartphone	home	PD	19	1.2 (0.9)	1.6 (1.4)	0.2	(-0.6, 0.4)
Gait speed (m/s) [mean]	Gait	1 minute walk	Smartphone	clinic	Controls	33	1.1 (0.2)	1.1 (0.2)	0.1	(-0.4, 0.2)
Gait speed (m/s) [mean]	Gait	1 minute walk	Smartphone	clinic	PD	27	1.0 (0.2)	1.0 (0.2)	-0.2	(-0.2, 0.6)
Gait speed (m/s) [mean]	Gait	1 minute walk	Smartphone	home	Controls	18	0.9 (0.2)	0.9 (0.2)	0.1	(-0.2, 0.5)
Gait speed (m/s) [mean]	Gait	1 minute walk	Smartphone	home	PD	19	0.9 (0.2)	0.9 (0.2)	0.4	(-0.6, 0.5)
Initial double support (s) [mean]	Gait	1 minute walk	Smartphone	clinic	Controls	34	0.1 (0.01)	0.1 (0.01)	-0.04	(-0.1, 0.5)
Initial double support (s) [mean]	Gait	1 minute walk	Smartphone	clinic	PD	27	0.1 (0.01)	0.1 (0.01)	-0.2	(-0.4, 0.3)
Initial double support (s) [mean]	Gait	1 minute walk	Smartphone	home	Controls	18	0.1 (0.02)	0.1 (0.01)	-0.03	(-0.6, 0.3)
Initial double support (s) [mean]	Gait	1 minute walk	Smartphone	home	PD	19	0.1 (0.01)	0.1 (0.02)	-0.002	(-0.4, 0.7)
Initial double support (s) asymmetry	Gait	1 minute walk	Smartphone	clinic	Controls	33	5.6 (4.3)	5.1 (3.7)	-0.1	(-0.3, 0.4)
Initial double support (s) asymmetry	Gait	1 minute walk	Smartphone	clinic	PD	25	5.6 (5.2)	6.1 (4.9)	0.1	(-0.4, 0.3)
Initial double support (s) asymmetry	Gait	1 minute walk	Smartphone	home	Controls	19	7.6 (6.2)	6.7 (5.3)	-0.1	(-0.3, 0.3)
Initial double support (s) asymmetry	Gait	1 minute walk	Smartphone	home	PD	14	7.4 (5.0)	7.4 (5.4)	-0.01	(-0.6, 0.4)
Single limb support (s) [mean]	Gait	1 minute walk	Smartphone	clinic	Controls	34	0.4 (0.04)	0.4 (0.03)	0.004	(-0.3, 0.5)
Single limb support (s) [mean]	Gait	1 minute walk	Smartphone	clinic	PD	27	0.4 (0.04)	0.4 (0.04)	-0.1	(-0.4, 0.3)
Single limb support (s) [mean]	Gait	1 minute walk	Smartphone	home	Controls	18	0.4 (0.04)	0.4 (0.04)	0.09	(-0.6, 0.2)
Single limb support (s) [mean]	Gait	1 minute walk	Smartphone	home	PD	19	0.4 (0.04)	0.4 (0.05)	0.1	(-0.3, 1.0)
Single limb support (s) asymmetry	Gait	1 minute walk	Smartphone	clinic	Controls	32	3.4 (2.3)	5.3 (3.8)	0.5	(-0.8, 0.05)
Single limb support (s) asymmetry	Gait	1 minute walk	Smartphone	clinic	PD	25	4.5 (2.5)	5.2 (3.5)	0.2	(0.2, 0.9)
Single limb support (s) asymmetry	Gait	1 minute walk	Smartphone	home	Controls	18	4.9 (2.8)	6.3 (5.4)	0.3	(-0.4, 0.3)
Single limb support (s) asymmetry	Gait	1 minute walk	Smartphone	home	PD	18	5.1 (2.6)	5.1 (3.6)	-0.003	(-0.2, 0.7)
Stance (s) [mean]	Gait	1 minute walk	Smartphone	clinic	Controls	34	0.7 (0.06)	0.7 (0.05)	-0.1	(-0.2, 0.6)
Stance (s) [mean]	Gait	1 minute walk	Smartphone	clinic	PD	27	0.7 (0.07)	0.7 (0.06)	-0.1	(-0.5, 0.2)
Stance (s) [mean]	Gait	1 minute walk	Smartphone	home	Controls	18	0.7 (0.07)	0.7 (0.06)	0.06	(-0.6, 0.2)
Stance (s) [mean]	Gait	1 minute walk	Smartphone	home	PD	19	0.7 (0.07)	0.7 (0.08)	0.07	(-0.3, 0.8)
Stance (s) asymmetry	Gait	1 minute walk	Smartphone	clinic	Controls	33	2.3 (1.6)	3.3 (2.3)	0.4	(-0.6, 0.1)
Stance (s) asymmetry	Gait	1 minute walk	Smartphone	clinic	PD	25	2.8 (1.5)	3.2 (2.1)	0.2	(0.1, 0.8)

Stance (s) asymmetry	Gait	1 minute walk	Smartphone	home	Controls	18	3.4 (2.4)	3.7 (3.3)	0.1	(-0.3, 0.4)
Stance (s) asymmetry	Gait	1 minute walk	Smartphone	home	PD	18	3.5 (2.3)	3.5 (2.4)	0.02	(-0.4, 0.6)
Stance (%GCT) [mean]	Gait	1 minute walk	Smartphone	clinic	Controls	34	0.6 (0.003)	0.6 (0.002)	-0.2	(0.07, 0.7)
Stance (%GCT) [mean]	Gait	1 minute walk	Smartphone	clinic	PD	27	0.6 (0.002)	0.6 (0.003)	-0.1	(-0.8, 0.08)
Stance (%GCT) [mean]	Gait	1 minute walk	Smartphone	home	Controls	17	0.6 (0.002)	0.6 (0.003)	-0.4	(-0.3, 0.2)
Stance (%GCT) [mean]	Gait	1 minute walk	Smartphone	home	PD	14	0.6 (0.002)	0.6 (0.004)	-0.8	(-1.0, 0.09)
Stance (%GCT) asymmetry	Gait	1 minute walk	Smartphone	clinic	Controls	34	2.2 (2.2)	3.2 (2.3)	0.3	(-0.7, 0.08)
Stance (%GCT) asymmetry	Gait	1 minute walk	Smartphone	clinic	PD	25	2.5 (1.4)	3.0 (2.1)	0.3	(0.005, 0.8)
Stance (%GCT) asymmetry	Gait	1 minute walk	Smartphone	home	Controls	18	2.7 (1.6)	3.7 (3.3)	0.3	(-0.4, 0.3)
Stance (%GCT) asymmetry	Gait	1 minute walk	Smartphone	home	PD	16	2.8 (1.4)	3.0 (2.2)	0.1	(-0.1, 0.8)
Step duration (s) [mean]	Gait	1 minute walk	Smartphone	clinic	Controls	34	0.5 (0.04)	0.5 (0.04)	-0.08	(-0.2, 0.5)
Step duration (s) [mean]	Gait	1 minute walk	Smartphone	clinic	PD	27	0.6 (0.06)	0.6 (0.05)	-0.1	(-0.5, 0.2)
Step duration (s) [mean]	Gait	1 minute walk	Smartphone	home	Controls	18	0.5 (0.06)	0.5 (0.05)	0.04	(-0.6, 0.2)
Step duration (s) [mean]	Gait	1 minute walk	Smartphone	home	PD	19	0.6 (0.06)	0.6 (0.07)	0.1	(-0.4, 0.8)
Step duration (s) asymmetry	Gait	1 minute walk	Smartphone	clinic	Controls	34	3.8 (2.8)	4.7 (3.1)	0.2	(-0.6, 0.2)
Step duration (s) asymmetry	Gait	1 minute walk	Smartphone	clinic	PD	25	4.1 (2.5)	4.8 (3.7)	0.2	(-0.08, 0.6)
Step duration (s) asymmetry	Gait	1 minute walk	Smartphone	home	Controls	18	3.9 (2.3)	5.3 (4.9)	0.3	(-0.2, 0.4)
Step duration (s) asymmetry	Gait	1 minute walk	Smartphone	home	PD	18	5.1 (3.3)	5.3 (3.8)	0.06	(-0.3, 0.6)
Step length (m) [mean]	Gait	1 minute walk	Smartphone	clinic	Controls	34	0.6 (0.09)	0.6 (0.08)	0.2	(-0.5, 0.08)
Step length (m) [mean]	Gait	1 minute walk	Smartphone	clinic	PD	27	0.6 (0.07)	0.6 (0.09)	-0.3	(-0.2, 0.6)
Step length (m) [mean]	Gait	1 minute walk	Smartphone	home	Controls	18	0.5 (0.08)	0.5 (0.08)	0.4	(-0.3, 0.4)
Step length (m) [mean]	Gait	1 minute walk	Smartphone	home	PD	19	0.5 (0.08)	0.5 (0.08)	0.6	(0.05, 0.7)
Step length (m) asymmetry	Gait	1 minute walk	Smartphone	clinic	Controls	34	5.0 (3.1)	5.6 (4.3)	0.1	(-0.3, 0.4)
Step length (m) asymmetry	Gait	1 minute walk	Smartphone	clinic	PD	27	7.3 (5.6)	7.9 (5.8)	0.09	(-0.2, 0.4)
Step length (m) asymmetry	Gait	1 minute walk	Smartphone	home	Controls	17	6.9 (3.2)	6.3 (4.6)	-0.09	(-0.3, 0.4)
Step length (m) asymmetry	Gait	1 minute walk	Smartphone	home	PD	19	7.7 (4.7)	9.0 (6.2)	0.2	(-0.8, 0.3)
Stride duration (s) [mean]	Gait	1 minute walk	Smartphone	clinic	Controls	34	1.1 (0.09)	1.1 (0.08)	-0.08	(-0.2, 0.4)
Stride duration (s) [mean]	Gait	1 minute walk	Smartphone	clinic	PD	27	1.1 (0.1)	1.1 (0.1)	-0.1	(-0.5, 0.2)
Stride duration (s) [mean]	Gait	1 minute walk	Smartphone	home	Controls	18	1.1 (0.1)	1.1 (0.09)	0.1	(-0.5, 0.2)
Stride duration (s) [mean]	Gait	1 minute walk	Smartphone	home	PD	19	1.1 (0.1)	1.1 (0.1)	0.09	(-0.3, 0.8)
Stride duration (s) asymmetry	Gait	1 minute walk	Smartphone	clinic	Controls	33	0.7 (0.4)	0.7 (0.5)	-0.09	(-0.2, 0.5)
Stride duration (s) asymmetry	Gait	1 minute walk	Smartphone	clinic	PD	26	0.8 (0.5)	0.8 (0.5)	0.1	(-0.4, 0.3)
Stride duration (s) asymmetry	Gait	1 minute walk	Smartphone	home	Controls	16	0.7 (0.5)	0.9 (0.9)	0.2	(-0.1, 0.5)
Stride duration (s) asymmetry	Gait	1 minute walk	Smartphone	home	PD	17	1.2 (0.9)	1.0 (0.8)	-0.1	(-0.4, 0.7)
Stride length (m) [mean]	Gait	1 minute walk	Smartphone	clinic	Controls	34	1.2 (0.2)	1.2 (0.2)	0.2	(-0.5, 0.09)
Stride length (m) [mean]	Gait	1 minute walk	Smartphone	clinic	PD	27	1.1 (0.1)	1.1 (0.2)	-0.4	(-0.1, 0.6)
Stride length (m) [mean]	Gait	1 minute walk	Smartphone	home	Controls	18	1.0 (0.2)	1.0 (0.2)	0.3	(-0.3, 0.4)
Stride length (m) [mean]	Gait	1 minute walk	Smartphone	home	PD	19	1.0 (0.2)	1.0 (0.2)	0.5	(-0.1, 0.6)
Stride length (m) asymmetry	Gait	1 minute walk	Smartphone	clinic	Controls	34	1.2 (0.9)	0.9 (0.5)	-0.3	(-0.03, 0.5)
Stride length (m) asymmetry	Gait	1 minute walk	Smartphone	clinic	PD	26	1.1 (0.6)	1.1 (0.6)	0.04	(-0.6, 0.08)
Stride length (m) asymmetry	Gait	1 minute walk	Smartphone	home	Controls	17	1.5 (1.2)	1.2 (1.1)	-0.2	(-0.1, 0.5)
Stride length (m) asymmetry	Gait	1 minute walk	Smartphone	home	PD	15	1.5 (0.9)	1.5 (1.4)	-0.01	(-0.7, 0.3)
Swing (s) [mean]	Gait	1 minute walk	Smartphone	clinic	Controls	34	0.4 (0.04)	0.4 (0.03)	-0.07	(-0.3, 0.4)
Swing (s) [mean]	Gait	1 minute walk	Smartphone	clinic	PD	27	0.4 (0.04)	0.4 (0.04)	-0.1	(-0.5, 0.3)
Swing (s) [mean]	Gait	1 minute walk	Smartphone	home	Controls	18	0.4 (0.04)	0.4 (0.03)	0.2	(-0.7, 0.01)
Swing (s) [mean]	Gait	1 minute walk	Smartphone	home	PD	19	0.4 (0.04)	0.4 (0.05)	0.1	(-0.2, 0.9)
Swing (s) asymmetry	Gait	1 minute walk	Smartphone	clinic	Controls	33	3.5 (2.9)	5.4 (4.1)	0.4	(-0.8, 0.02)
Swing (s) asymmetry	Gait	1 minute walk	Smartphone	clinic	PD	25	4.1 (2.5)	5.0 (3.6)	0.4	(0.04, 0.8)
Swing (s) asymmetry	Gait	1 minute walk	Smartphone	home	Controls	18	5.3 (4.0)	6.1 (5.6)	0.2	(-0.3, 0.3)
Swing (s) asymmetry	Gait	1 minute walk	Smartphone	home	PD	17	5.0 (2.4)	4.8 (3.6)	-0.05	(-0.4, 0.6)
Terminal double support (s) [mean]	Gait	1 minute walk	Smartphone	clinic	Controls	34	0.1 (0.01)	0.1 (0.01)	-0.1	(-0.1, 0.6)
Terminal double support (s) [mean]	Gait	1 minute walk	Smartphone	clinic	PD	27	0.1 (0.01)	0.1 (0.01)	-0.08	(-0.5, 0.2)
Terminal double support (s) [mean]	Gait	1 minute walk	Smartphone	home	Controls	18	0.1 (0.01)	0.1 (0.01)	0.1	(-0.6, 0.2)
Terminal double support (s) [mean]	Gait	1 minute walk	Smartphone	home	PD	19	0.1 (0.01)	0.1 (0.01)	-0.09	(-0.3, 0.9)
Terminal double support (s) asymmetry	Gait	1 minute walk	Smartphone	clinic	Controls	32	4.7 (2.6)	5.1 (3.7)	0.1	(-0.3, 0.3)
Terminal double support (s) asymmetry	Gait	1 minute walk	Smartphone	clinic	PD	25	5.3 (5.4)	6.3 (5.1)	0.3	(-0.2, 0.5)
Terminal double support (s) asymmetry	Gait	1 minute walk	Smartphone	home	Controls	17	6.3 (4.3)	6.4 (5.3)	0.01	(-0.4, 0.2)
Terminal double support (s) asymmetry	Gait	1 minute walk	Smartphone	home	PD	16	6.0 (3.7)	6.8 (3.9)	0.2	(-0.6, 0.5)
Arm swing (degrees) [mean]	Gait	1 minute walk	Smartwatch	clinic	Controls	35	47.8 (19.2)	48.4 (20.8)	0.06	(-0.3, 0.3)
Arm swing (degrees) [mean]	Gait	1 minute walk	Smartwatch	clinic	PD	27	25.1 (13.7)	21.7 (14.6)	-0.3	(-0.3, 0.4)
Arm swing (degrees) [mean]	Gait	1 minute walk	Smartwatch	home	Controls	19	44.6 (14.7)	43.0 (18.5)	-0.1	(-0.5, 0.5)
Arm swing (degrees) [mean]	Gait	1 minute walk	Smartwatch	home	PD	19	21.7 (12.1)	20.3 (13.9)	-0.2	(-0.6, 0.3)
MDS - UPDRS part I total	MDS - UPDRS		Clinical rating	clinic	Controls	45	3.0 (2.6)	3.1 (3.6)	0.04	(-0.4, 0.3)
MDS - UPDRS part I total	MDS - UPDRS		Clinical rating	clinic	PD	47	5.6 (3.8)	5.9 (4.2)	0.08	(-0.3, 0.3)
MDS - UPDRS part I + II + III total	MDS - UPDRS		Clinical rating	clinic	Controls	45	6.1 (5.4)	6.5 (5.1)	0.09	(-0.5, 0.3)
MDS - UPDRS part I + II + III total	MDS - UPDRS		Clinical rating	clinic	PD	47	33.7 (12.6)	40.6 (14.3)	0.6	(-0.2, 0.4)
MDS - UPDRS part II total	MDS - UPDRS		Clinical rating	clinic	Controls	45	0.5 (1.1)	0.4 (1.1)	-0.03	(-0.3, 0.4)
MDS - UPDRS part II total	MDS - UPDRS		Clinical rating	clinic	PD	47	5.4 (3.8)	7.4 (4.8)	0.6	(-0.4, 0.2)
MDS - UPDRS part III total	MDS - UPDRS		Clinical rating	clinic	Controls	45	2.6 (3.6)	3.0 (3.3)	0.1	(-0.5, 0.3)
MDS - UPDRS part III total	MDS - UPDRS		Clinical rating	clinic	PD	47	22.7 (10.7)	27.3 (11.4)	0.6	(-0.2, 0.4)
Rater evaluation of speech	Speech	item 3.01	Clinical rating	clinic	Controls	45	0.07 (0.3)	0.09 (0.3)	0.09	(-0.3, 0.3)
Rater evaluation of speech	Speech	item 3.01	Clinical rating	clinic	PD	47	0.4 (0.5)	0.7 (0.5)	0.6	(-0.2, 0.3)

Self-reported problem with speech	Speech	item 2.01	Self reported evalution	clinic	Controls	46	0 (0)	0 (0)		
Self-reported problem with speech	Speech	item 2.01	Self reported evalution	clinic	PD	47	0.4 (0.7)	0.5 (0.7)	0.2	(0.1, 0.4)
Cepstral peak prominence, zscored	Speech	Phonation	Smartphone	clinic	Controls	25	-0.009 (1.0)	0.05 (1.0)	0.07	(-0.08, 0.5)
Cepstral peak prominence, zscored	Speech	Phonation	Smartphone	clinic	PD	34	-0.1 (1.2)	-0.3 (1.4)	-0.1	(-0.4, 0.4)
Cepstral peak prominence, zscored	Speech	Phonation	Smartphone	home	Controls	26	0.09 (0.9)	0.3 (0.9)	0.2	(-0.3, 0.4)
Cepstral peak prominence, zscored	Speech	phonation	Smartphone	home	PD	31	-0.2 (1.1)	-0.4 (1.0)	-0.2	(-0.2, 0.8)
Log(pause time), zscored	Speech	Reading	Smartphone	clinic	Controls	25	0.02 (1.0)	0.1 (1.0)	0.1	(-0.2, 0.4)
Log(pause time), zscored	Speech	Reading	Smartphone	clinic	PD	34	0.3 (1.2)	0.5 (1.1)	0.2	(-0.2, 0.7)
Log(pause time), zscored	Speech	Reading	Smartphone	home	Controls	26	-0.3 (0.9)	0.04 (0.8)	0.4	(-0.009, 0.6)
Log(pause time), zscored	Speech	Reading	Smartphone	home	PD	31	-0.1 (1.0)	0.2 (1.2)	0.3	(-0.06, 1.0)
MFCC2, zscored	Speech	Phonation	Smartphone	clinic	Controls	25	0.2 (0.9)	0.04 (0.8)	-0.2	(-0.3, 0.3)
MFCC2, zscored	Speech	Phonation	Smartphone	clinic	PD	34	-0.3 (1.0)	-0.2 (1.1)	0.04	(-0.6, 0.2)
MFCC2, zscored	Speech	Phonation	Smartphone	home	Controls	26	0.07 (1.3)	-0.2 (1.1)	-0.2	(-0.3, 0.3)
MFCC2, zscored	Speech	Phonation	Smartphone	home	PD	31	-0.3 (1.0)	-0.4 (1.6)	-0.09	(-0.6, 0.3)
Monopitch (semitone range), zscored	Speech	Reading	Smartphone	clinic	Controls	25	-0.2 (1.0)	0.2 (1.0)	0.5	(-0.08, 0.4)
Monopitch (semitone range), zscored	Speech	Reading	Smartphone	clinic	PD	34	-0.8 (0.9)	-0.6 (0.9)	0.3	(0.1, 0.9)
Monopitch (semitone range), zscored	Speech	Reading	Smartphone	home	Controls	26	0.06 (1.1)	0.2 (0.9)	0.1	(-0.01, 0.5)
Monopitch (semitone range), zscored	Speech	Reading	Smartphone	home	PD	31	-0.7 (0.9)	-0.2 (1.1)	0.5	(-0.3, 0.5)
Speech composite	Speech	Reading, phonation	Smartphone	clinic	Controls	25	0.03 (2.0)	-0.1 (1.8)	-0.09	(-0.3, 0.1)
Speech composite	Speech	Reading, phonation	Smartphone	clinic	PD	34	1.5 (2.5)	1.7 (2.0)	0.05	(-0.5, 0.3)
Speech composite	Speech	Reading, phonation	Smartphone	home	Controls	26	-0.6 (2.0)	-0.2 (1.5)	0.1	(-0.2, 0.3)
Speech composite	Speech	Reading, phonation	Smartphone	home	PD	31	1.1 (1.8)	1.1 (2.0)	-0.004	(-0.2, 0.6)
Rater evaluation of constancy of tremor	Tremor	item 3.18	Clinical rating	clinic	Controls	45	0 (0)	0.02 (0.1)	0.1	(-0.6, 0.2)
Rater evaluation of constancy of tremor	Tremor	item 3.18	Clinical rating	clinic	PD	47	1.7 (1.4)	2.1 (1.6)	0.5	(0.1, 0.3)
Self-reported problems with tremor	Tremor	item 2.10	Self reported evalution	clinic	Controls	48	0 (0)	0 (0)		
Self-reported problems with tremor	Tremor	item 2.10	Self reported evalution	clinic	PD	47	1.2 (0.7)	1.4 (0.8)	0.4	(0.2, 0.5)
Tremor (%)	Tremor	Passive	Smartwatch	home	Controls	10	0.8 (0.6)	1.1 (1.3)	0.3	(-0.2, 0.4)
Tremor (%)	Tremor	Passive	Smartwatch	home	PD	34	23.5 (18.2)	30.2 (21.3)	0.4	(-0.4, 0.8)
Turns angle (degrees) [mean]	Turns	2 minute walk	Research accel.	clinic	Controls	45	182.5 (7.8)	182.5 (4.6)	0.002	(-0.3, 0.3)
Turns angle (degrees) [mean]	Turns	2 minute walk	Research accel.	clinic	PD	47	178.2 (6.4)	176.6 (8.2)	-0.2	(-0.2, 0.4)
Turns duration (s) [mean]	Turns	2 minute walk	Research accel.	clinic	Controls	45	2.1 (0.4)	2.1 (0.4)	-0.07	(-0.3, 0.6)
Turns duration (s) [mean]	Turns	2 minute walk	Research accel.	clinic	PD	47	2.3 (0.3)	2.4 (0.4)	0.4	(-0.4, 0.2)
Turns steps in.turn_ [mean]	Turns	2 minute walk	Research accel.	clinic	Controls	44	3.7 (0.8)	3.6 (0.7)	-0.2	(-0.1, 0.6)
Turns steps in.turn_ [mean]	Turns	2 minute walk	Research accel.	clinic	PD	47	3.9 (0.6)	4.0 (0.7)	0.4	(-0.5, 0.1)
Turns turn velocity (degrees) (s) [mean]	Turns	2 minute walk	Research accel.	clinic	Controls	45	202.3 (37.4)	206.5 (47.1)	0.1	(-0.6, 0.2)
Turns turn velocity (degrees) (s) [mean]	Turns	2 minute walk	Research accel.	clinic	PD	47	166.4 (30.0)	157.8 (31.7)	-0.3	(-0.2, 0.4)
Turns angle (degrees) [mean]	Turns	Dual task	Research accel.	clinic	Controls	46	179.6 (6.3)	181.3 (6.9)	0.2	(-0.5, 0.2)
Turns angle (degrees) [mean]	Turns	Dual task	Research accel.	clinic	PD	46	175.9 (9.7)	176.5 (7.6)	0.08	(-0.08, 0.6)
Turns duration (s) [mean]	Turns	Dual task	Research accel.	clinic	Controls	46	2.1 (0.3)	2.2 (0.3)	0.1	(-0.4, 0.3)
Turns duration (s) [mean]	Turns	Dual task	Research accel.	clinic	PD	46	2.4 (0.4)	2.5 (0.4)	0.2	(-0.2, 0.4)
Turns steps in turn [mean]	Turns	Dual task	Research accel.	clinic	Controls	44	3.6 (0.7)	3.5 (0.8)	-0.07	(-0.3, 0.5)
Turns steps in turn [mean]	Turns	Dual task	Research accel.	clinic	PD	46	3.9 (0.7)	4.1 (0.7)	0.3	(-0.3, 0.2)
Turns turn velocity (degrees) (s) [mean]	Turns	Dual task	Research accel.	clinic	Controls	46	192.8 (35.6)	197.9 (41.7)	0.2	(-0.6, 0.2)
Turns turn velocity (degrees) (s) [mean]	Turns	Dual task	Research accel.	clinic	PD	46	158.5 (31.0)	150.6 (30.0)	-0.3	(-0.2, 0.4)
Turn duration (s) [mean]	Turns	1 minute walk	Smartphone	clinic	Controls	41	2.1 (0.5)	2.1 (0.4)	0.01	(-0.3, 0.4)
Turn duration (s) [mean]	Turns	1 minute walk	Smartphone	clinic	PD	38	2.3 (0.5)	2.4 (0.5)	0.2	(-0.3, 0.3)
Turn duration (s) [mean]	Turns	1 minute walk	Smartphone	home	Controls	30	2.3 (0.5)	2.1 (0.6)	-0.3	(-0.4, 0.3)
Turn duration (s) [mean]	Turns	1 minute walk	Smartphone	home	PD	27	2.2 (0.5)	2.4 (0.5)	0.4	(-0.7, 0.06)

Supplementary Table 4: Sample Size per arm by treatment effect and standardized change (assuming power = 0.8 and 1-sided $\alpha = 0.025$)

	Treatment Effect			
Standardized Change	20%	30%	40%	50%
0.1	39246	17443	9813	6281
0.2	9813	4362	2454	1571
0.3	4362	1939	1092	699
0.4	2454	1092	615	394
0.5	1571	699	394	253
0.6	1092	486	274	176
0.7	802	357	202	130
0.8	615	274	155	100
0.9	486	217	123	79
1.0	394	176	100	64
1.1	326	146	83	53
1.2	274	123	70	45
1.3	234	105	60	39
1.4	202	90	52	34
1.5	176	79	45	29
1.6	155	70	40	26
1.7	137	62	35	23
1.8	123	55	32	21
1.9	110	50	29	19
2.0	100	45	26	17
