# Supplementary material for: Using a smartwatch and smartphone to assess early Parkinson’s disease in the WATCH-PD study over 12 months
Source: NPJ Parkinsons Dis. 2024 Jun 12;10:112. doi: 10.1038/s41531-024-00721-2 (PMC11169239; doi:10.1038/s41531-024-00721-2)
Supplement: Supplementary file 2 — Reporting Summary [file 41531_2024_721_MOESM2_ESM.pdf]

nature portfolio

Corresponding author(s): Jamie Adams, MD

Last updated by author(s): 5/4/2024

Reporting Summary

Nature Portfolio wishes to improve the reproducibility of the work that we publish. This form provides structure for consistency and transparency in reporting. For further information on Nature Portfolio policies, see our Editorial Policies and the Editorial Policy Checklist.

Statistics

For all statistical analyses, confirm that the following items are present in the figure legend, table legend, main text, or Methods section.

n/a	Confirmed	
☐	☒	The exact sample size (*n*) for each experimental group/condition, given as a discrete number and unit of measurement
☐	☒	A statement on whether measurements were taken from distinct samples or whether the same sample was measured repeatedly
☐	☒	The statistical test(s) used AND whether they are one- or two-sided<br>*Only common tests should be described solely by name; describe more complex techniques in the Methods section.*
☐	☒	A description of all covariates tested
☐	☒	A description of any assumptions or corrections, such as tests of normality and adjustment for multiple comparisons
☐	☒	A full description of the statistical parameters including central tendency (e.g. means) or other basic estimates (e.g. regression coefficient) AND variation (e.g. standard deviation) or associated estimates of uncertainty (e.g. confidence intervals)
☐	☒	For null hypothesis testing, the test statistic (e.g. *F*, *t*, *r*) with confidence intervals, effect sizes, degrees of freedom and *P* value noted<br>*Give P values as exact values whenever suitable.*
☒	☐	For Bayesian analysis, information on the choice of priors and Markov chain Monte Carlo settings
☐	☒	For hierarchical and complex designs, identification of the appropriate level for tests and full reporting of outcomes
☐	☒	Estimates of effect sizes (e.g. Cohen's *d*, Pearson's *r*), indicating how they were calculated

Our web collection on statistics for biologists contains articles on many of the points above.

Software and code

Policy information about availability of computer code

Data collection

This study used three devices (to collect data: research-grade wearable "Opal" sensors (APDM Wearable Technologies, a Clario Company), an Apple Watch 4 or 5, and an iPhone 10 or 11 (Apple, Inc.) running a smartphone application specifically for PD (BrainBaseline™). Raw mobility and speech signals were recorded from Apple's native accelerometer (100 Hz sampling rate) and microphone (32 kHz sampling rate) hardware configurations.

Data analysis

Accelerometry data and tremor scores were collected from the smartwatch via Apple's Movement Disorders Application Programming Interface (developer.apple.com/documentation/coremotion/getting_movement_disorder_symptom_data). The Movement Disorders API (open source code available at https://github.com/ResearchKit/mPower) generates tremor classification scores (none, slight, mild, moderate, strong, or unknown) for each 1-minute period, and the fraction of time spent in each category was calculated for each participant.

Gait features were extracted from the smartwatch and smartphone using software developed in-house. Gait bouts were identified after turns, and gait features during each bout were extracted using open-source GaitPy. Arm swing features were calculated using rotational velocity from the smartwatch. Movement data was collected from the wearable sensors using Mobility Lab software (APDM Wearable Technologies, a Clario Company), and measures were extracted using custom algorithms written in Python (Wilmington, DE).

For speech analysis, phonation and reading files were processed using custom Python code with features computed using the Parselmouth interface to Praat and the Librosa library.

For manuscripts utilizing custom algorithms or software that are central to the research but not yet described in published literature, software must be made available to editors and reviewers. We strongly encourage code deposition in a community repository (e.g. GitHub). See the Nature Portfolio guidelines for submitting code & software for further information.

Data

Policy information about availability of data

All manuscripts must include a data availability statement. This statement should provide the following information, where applicable:

- Accession codes, unique identifiers, or web links for publicly available datasets
- A description of any restrictions on data availability
- For clinical datasets or third party data, please ensure that the statement adheres to our policy

The data are available to members of the Critical Path for Parkinson's Consortium 3DT Initiative Stage 2. For those who are not a part of 3DT Stage 2, a proposal may be made to the WATCH-PD Steering Committee (via the corresponding author) for de-identified datasets.

Human research participants

Policy information about studies involving human research participants and Sex and Gender in Research.

Reporting on sex and gender	Like other PD studies, the PD sample was predominantly male (46 males out of 82 people at baseline, 32 males out 57 for people who completed the 12 month visit). The control sample was predominantly female (18 males out of 50 people at baseline, 18 males out 49 for people who completed the 12 month visit).
Population characteristics	Eighty-two individuals with early, untreated Parkinson's disease and 50 age-matched controls were enrolled. The PD population was 56% male and disease duration was 10 months on average at enrollment with Hoehn and Yahr scores <=2. Controls were predominantly female and without significant medical or neurologic co-morbidities as determined by site investigators. Like many other PD studies, the study population was predominantly white (95% at baseline, 96% at month 12) and have > 12 years of education (95% at both baseline and month 12).
Recruitment	Participants were recruited from 17 Parkinson Study Group sites in the United States. Some sites used social media and research registries to help with recruitment.
Ethics oversight	The WCG Institutional Review Board approved the procedures used in the study, and there was full compliance with human experimentation guidelines.

Note that full information on the approval of the study protocol must also be provided in the manuscript.

Field-specific reporting

Please select the one below that is the best fit for your research. If you are not sure, read the appropriate sections before making your selection.

☒ Life sciences ☐ Behavioural & social sciences ☐ Ecological, evolutionary & environmental sciences

For a reference copy of the document with all sections, see nature.com/documents/nr-reporting-summary-flat.pdf

Life sciences study design

All studies must disclose on these points even when the disclosure is negative.

Sample size	The study was powered to detect a mean change over 12 months for a digital endpoint with superior responsiveness to MDS-UPDRS Part III. The mean change in part III from baseline to year one in individuals with early, untreated PD in the PPMI study was 6.9 with a standard deviation of 7.0.55 Allowing for up to half of participants to begin dopaminergic therapy over 12 months and 15% drop out, the study aimed to recruit at least 75 participants with PD to yield 30 participants completing the study off medication. The study had more than 95% power to detect a true change of 6.9 units using a one-sample t-test and a two-tailed 5% significance.
Data exclusions	If a participant had missing data for an outcome or as part of a necessary algorithm, that person was excluded for that analysis. Values of zero (i.e., did not attempt the task) were also excluded. Participants were dropped from analysis after they started symptomatic treatment.
Replication	Test-retest reliability was performed
Randomization	Observational study, no randomization
Blinding	Observational study, no blinding

Reporting for specific materials, systems and methods

We require information from authors about some types of materials, experimental systems and methods used in many studies. Here, indicate whether each material, system or method listed is relevant to your study. If you are not sure if a list item applies to your research, read the appropriate section before selecting a response.

Materials & experimental systems

n/a	Involved in the study
☒	☐ Antibodies
☒	☐ Eukaryotic cell lines
☒	☐ Palaeontology and archaeology
☒	☐ Animals and other organisms
☒	☐ Clinical data
☒	☐ Dual use research of concern

Methods

n/a	Involved in the study
☒	☐ ChIP-seq
☒	☐ Flow cytometry
☒	☐ MRI-based neuroimaging
